# A global review of the state of the evidence of household air pollution’s contribution to ambient fine particulate matter and their related health impacts

**DOI:** 10.1016/j.envint.2023.107835

**Published:** 2023-02-18

**Authors:** Sourangsu Chowdhury, Ajay Pillarisetti, Alicia Oberholzer, James Jetter, John Mitchell, Eva Cappuccilli, Borgar Aamaas, Kristin Aunan, Andrea Pozzer, Donee Alexander

**Affiliations:** a CICERO Center for International Climate Research, Oslo, Norway; b University of California, Berkeley, Berkeley, USA; c Clean Cooking Alliance, Washington, D.C., USA; d United States Environmental Protection Agency, Washington, D.C., USA; e Max Planck Institute for Chemistry, Mainz, Germany

## Abstract

Direct exposure to household fine particulate air pollution (HAP) associated with inefficient combustion of fuels (wood, charcoal, coal, crop residues, kerosene, etc.) for cooking, space-heating, and lighting is estimated to result in 2.3 (1.6–3.1) million premature yearly deaths globally. HAP emitted indoors escapes outdoors and is a leading source of outdoor ambient fine particulate air pollution (AAP) in low- and middle-income countries, often being a larger contributor than well-recognized sources including road transport, industry, coal-fired power plants, brick kilns, and construction dust. We review published scientific studies that model the contribution of HAP to AAP at global and major sub-regional scales. We describe strengths and limitations of the current state of knowledge on HAP’s contribution to AAP and the related impact on public health and provide recommendations to improve these estimates. We find that HAP is a dominant source of ambient fine particulate matter (PM2.5) globally — regardless of variations in model types, configurations, and emission inventories used — that contributes approximately 20 % of total global PM2.5 exposure. There are large regional variations: in South Asia, HAP contributes ~ 30 % of ambient PM2.5, while in high-income North America the fraction is ~ 7 %. The median estimate indicates that the household contribution to ambient air pollution results in a substantial premature mortality burden globally of about 0.77(0.54–1) million excess deaths, in addition to the 2.3 (1.6–3.1) million deaths from direct HAP exposure. Coordinated global action is required to avert this burden.

## 1. Introduction

In the twenty-first century, exposure to air pollution is ubiquitous; the resulting impacts on health, the environment, and welfare are well-described: 6.4 (95 % uncertainty bounds: 5.7–7.3) million premature deaths and 209 (95 % UB: 185–235) million disability adjusted life years (DALYs) globally (Murray et al., 2020). Most governments acknowledge the health effects of air pollution exposure and have mitigation plans in place, though they are implemented and enforced at varying scales and efficacies. These mitigation plans must cope with many competing interests: industrial and economic motivations, the need for inexpensive energy, environmental concerns, and human welfare. And, fundamentally, mitigation planners must understand what sources emit what pollutants; how those emissions impact people and the environment; and the health, environmental, and economic benefits and impacts of control measures deemed effective.

Despite our modern era of development, broad global industrialization, and increasing rates of electrification, one of the earliest anthropogenic sources of air pollution – the use of solid fuels (like firewood) in homes for cooking, heating, and other energy services – remains one of the largest sources of air pollution globally (Chowdhury et al., 2022; Lelieveld et al., 2015), with a significant footprint in low- and middle-income countries (LMICs). Primary emissions, like soot and organic carbon from solid fuel combustion, are so high that every 1 million households using solid fuels release emissions approximately equivalent to 2.3 million diesel trucks (meeting 2010 Euro 4 standards) (Smith and Pillarisetti, 2017). The resulting ‘household air pollution’ (HAP) contributes to ~ 36 % of premature deaths and 44 % of DALYs of the total burden from air pollution among the population who uses these primitive fuels to meet basic energy needs (Murray et al., 2020). As of 2016, approximately 3.8 billion people use solid fuels for cooking and other household activities like heating and lighting, despite progress to address global poverty and development in LMICs (HEI, 2018; Pillarisetti et al., 2022).

In households with limited ventilation (as is common in LMICs), exposures to HAP, particularly those of women and young children, have been measured to be many times higher than World Health Organization (WHO) guidelines and national air quality standards (Balakrishnan et al., 2013; Pillarisetti et al., 2022; Smith, 1993; Smith et al., 2014). Ambient air pollution (AAP) from a variety of sources, including agricultural waste burning, industries, power generation, vehicular emissions, and road and natural dust also infiltrates households and contributes to in-home exposure (Leung, 2015; Raunemaa et al., 1989). Likewise, activities involving combustion in households also impact ambient air quality (Chafe et al., 2014; Chowdhury et al., 2019b). Until recently, the scale and magnitude of HAP’s contribution to AAP was under-recognized and poorly characterized (Chowdhury et al., 2019a; Pillarisetti et al., 2022; Smith et al., 2014). Basics of HAP and AAP, their measurement methods and related health outcomes are discussed in Supplementary Information (SI Text 1) and elsewhere (Pillarisetti et al., 2022).

While common sense tells us that some fraction of what is emitted indoors must go outdoors, estimates of the proportion of AAP attributable to household sources vary widely (Chowdhury et al., 2019a). Take, for example, three estimates spread over the past decade. An influential early study described in Chafe et al., (2014) estimated that cooking-related HAP contributes 12 % of the total, population- weighted ambient PM2.5 exposure globally. A subsequent study for the year 2010, (Lelieveld et al., 2015) modeled that 31 % of ambient PM2.5 may be associated with household solid fuel use. Finally, a more recent study (McDuffie et al., 2021) using updated emissions data found 19 % of ambient PM2.5 attributable to HAP.

Though the contribution of HAP to AAP is expected to decline over time in most LMICs as solid fuel use decreases, the total exposed population may increase due to population growth (HEI, 2018; Pillarisetti et al., 2022). Additionally, many current estimates do not account for non-cooking activities, like heating, lighting, heating water, and animal fodder preparation, and thus may underestimate the true contribution. Finally, in Africa and South Asia, the contribution of HAP to AAP has been estimated to be very high, (e.g., up to ~ 50 % in India) (Chowdhury et al., 2022, 2019b; Conibear et al., 2018; Upadhyay et al., 2018). In these regions, ambient air quality standards may not be attainable without a transition to clean household fuels (e.g. LPG, ethanol, electric cooking) (Chowdhury et al., 2022; McDuffie et al., 2021).

As energy demands increase in LMICs, the choice of cooking fuel may have major implications for management of ambient air quality and public health. To achieve improvements in ambient air quality and facilitate mitigation efforts, countries need an understanding of HAP’s contribution to AAP. The goal of this review is (i) to describe the current state of knowledge on HAP’s contribution to AAP and its impact on public health; (ii) to discuss the modeling and measurement techniques used to generate these estimates and associated uncertainties; (iii) to make recommendations to improve the estimates of HAP’s impact on AAP and health; (iv) to discuss regional policies that may impact HAP and its contribution to AAP; and (v) to discuss next steps for improving these estimates.

## 2. Modeling contributions of HAP to ambient PM2.5

PM2.5 pollution varies in spatial distribution, composition, and sources (Chowdhury et al., 2022; Hammer et al., 2020; Lelieveld et al., 2015; McDuffie et al., 2021; Snider et al., 2016). In general, local sources, such as transportation, dominate in urban areas, while in the background and rural areas, ‘imported’ pollution (transported pollution) dominates (Donnelly et al., 2015; Junker et al., 2009; National Research Council, 2010). However, in regions where solid fuel use is dominant, a significant portion of ambient PM2.5 pollution in rural and background regions is associated with household emissions that escape outdoors (Chowdhury et al., 2022; Conibear et al., 2018; HEI, 2019; Herich et al., 2014; Puxbaum et al., 2007; Upadhyay et al., 2018).

Understanding the drivers of degraded ambient air quality requires understanding sources of air pollution and the magnitude of their emissions. Source apportionment refers to the practice of obtaining information about source sectors (e.g., transportation, power, industry, residential, commercial, agricultural, construction, and natural) that contribute emissions of the pollutant of interest (Liao et al., 2017; Mircea et al., 2020; Thunis et al., 2018). Knowing what sector contributes what fraction of a given contaminant enables control prioritization strategies. Source apportionment methodologies are complex and thus some uncertainty persists.

There are two methods for assessing sources of air pollutants, discussed below. Both approaches reconstruct the atmospheric concentration of pollutants associated with sector-specific emission sources. These distinct methods when operated in tandem serve as an efficient tool for identifying major sources of PM2.5 and supporting air quality management.

### 2.1. Top-down approach

In a top-down approach, ambient samples are collected in locations of interest, analyzed for their chemical composition (Abulude, 2017; Guttikunda, 2009; Johnson et al., 2011), and then related to specific emission sources from that location using models. Emission sources have largely distinct chemical fingerprints, enabling this approach. For example, universal markers of biomass burning in households are anhydro-saccharides (mannosan, galactosan and levoglucosan), methoxyphenols, and potassium. Similarly, iron and steel plants emit aerosols rich in iron. More details can be found in (Banerjee et al., 2015; Hopke, 2016; Hopke et al., 2020). Receptor modeling methodologies like chemical mass balance (CMB) models or positive factorization (PMF) models (Hopke, 2016; Hopke et al., 2020) are then used to estimate the relative contributions of different sources to the total particulate matter measured in the first step, based on source-specific chemical fingerprints.

### 2.2. Bottom-up approach

Bottom-up modeling approaches utilize sector-specific emissions information, derived from emission inventories, fed into complex earth system models, chemical general circulation models, or chemical transport models (Brasseur and Jacob, 2017) that predict pollutant concentrations for a given location and time (Bey et al., 2001; Clappier et al., 2017; Joeckel et al., 2010; Johnson et al., 2011; Thunis et al., 2019). These models use a set of numerical equations and incorporate physical and chemical parameters such as precipitation microphysics, longwave and shortwave radiation, land surface classification, convective parameterization, gas-phase chemistry, photolysis, aerosols, natural dust, initial and boundary conditions for chemistry, and meteorology.

**Fig. 1.**
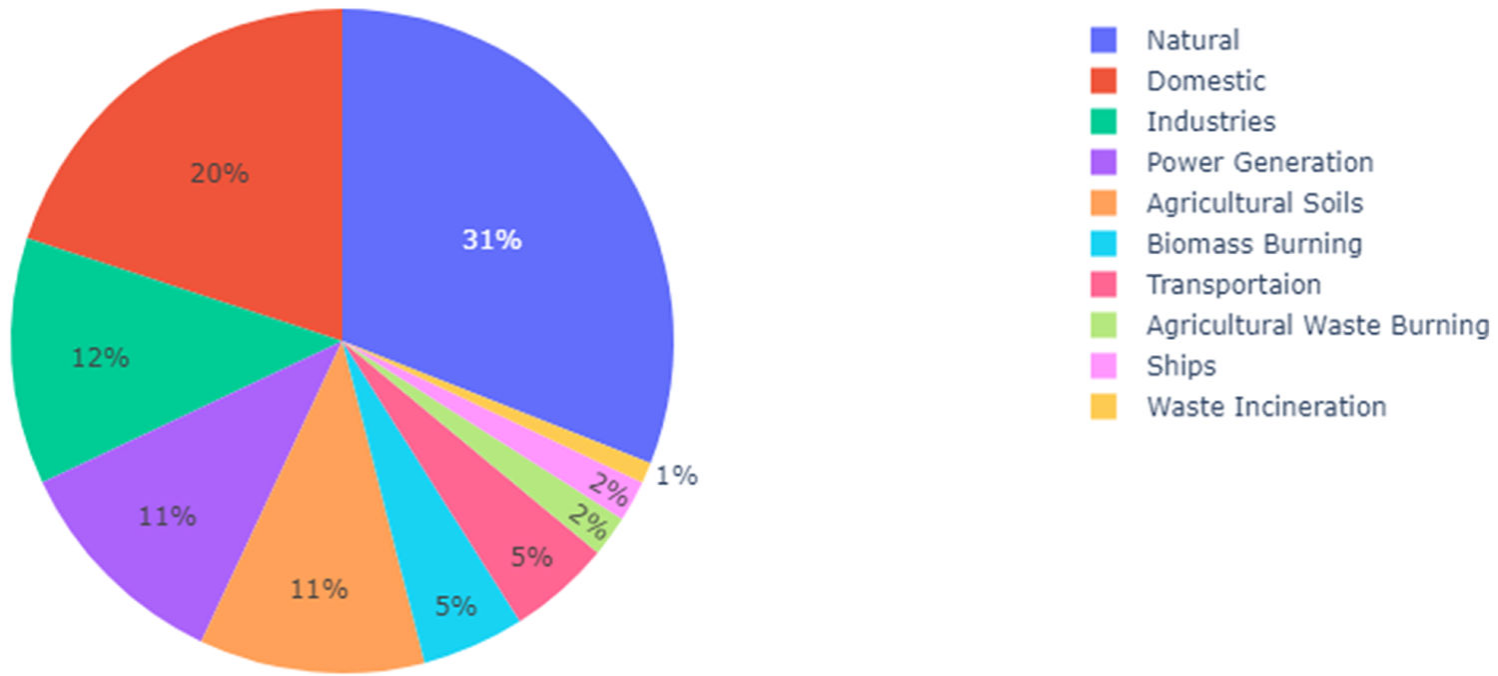
Contribution of major emission sources to ambient air pollution (PM2.5) globally. (Reproduced with data from Chowdhury et al.,(2022)).

These complex atmospheric chemistry models can be characterized as global, regional, or local based on their extent. It should be noted that simulating chemical transport models is computationally expensive; therefore, global models generally have coarse spatial resolution and are often not able to account for changes in inputs like meteorology and emissions at a local scale. Regional chemical transport models, however, are simulated over a more specific geographic domain, often at very fine spatial resolution (i.e. 1 km2). As an example, the contribution of different sources to global population weighted PM2.5 from a recent study (Chowdhury et al., 2022) is depicted in Fig. 1.

Bottom-up approaches are not all identical. Many use different inputs, emissions inventories, model configurations, and meteorological datasets, leading to varied results between studies. This is neither unusual nor surprising as these models are developed by distinct groups to answer unique questions. Quantifying the magnitude of the change between estimates is challenging unless a detailed model intercomparison study is carried out – for instance, evaluating different possible combinations of meteorological datasets and emissions inventories. This variability is distinct from model error due to propagation of internal uncertainties in model equations, in the meteorological data, and in formulation of emission inventories. Here, we focus on bottom-up studies of the contribution of HAP to AAP and discuss the major factors that may influence estimates from such efforts. We discuss the major factors (choice of emission inventories, model types and configurations and health impact assessment methodology) that may influence a bottom-up source contribution study with a focus on the contribution of HAP to AAP and related health impacts in the SI Text 2.

## 3. Methodology

**Fig. 2.**
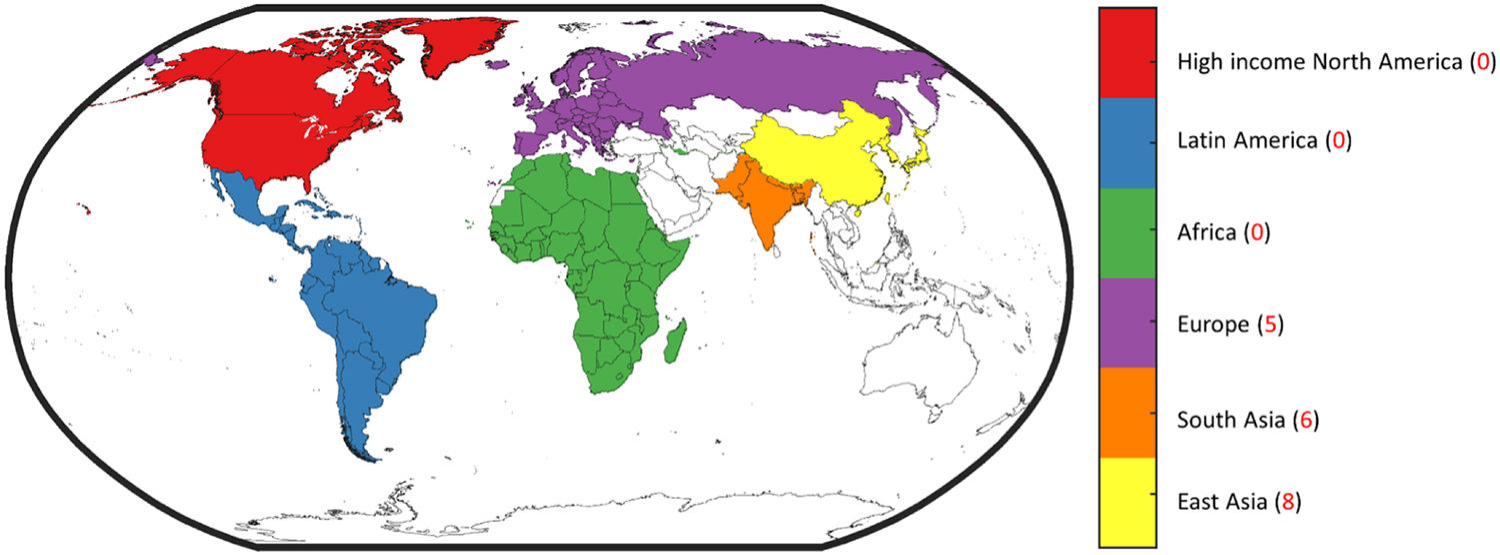
The regions considered for the review. The digits in parenthesis indicate the number of regional studies in addition to information from 9 global studies considered for each region.

We conducted a literature search using PubMed (https://pubmed.ncbi.nlm.nih.gov/). We limited our search to journal articles published in English from January 2001 to January 2022 focusing on all studies that report the contribution of specific sources to ambient air pollution exposure and health impacts. We used the following search terms: ((((((((“ambient air pollution”[All Fields]) AND (household air pollution)) OR (indoor air pollution)) OR (residential)) AND (global)) AND (global model)) AND (sources)) AND (health burden))” on PubMed. The search terms returned 25 results, of which 20 were rejected based on unavailability of full text and for not qualifying as bottom-up modeling studies reporting contribution of HAP to ambient PM2.5. The relevant studies underwent full text review. In addition, an independent search was performed and the references of identified studies were checked to find additional studies by subjective judging, which returned a further 4 studies. The studies were then tabularized (Table 1). A flowchart outline the search strategies is depicted in Fig. S1. Similar searches were performed for the following geographies: ‘Global’, ‘East Asia’, ‘South Asia’, ‘Europe’, ‘sub-Saharan Africa’, ‘Latin America’, and ‘High income North America’ and are listed in (S1-S6) by their study extent. Fig. 2 depicts the location of the regions and the respective number of regional and global studies considered in this review.

## 4. A global perspective and recent regional advances

This section explains the similarities and differences between modeled estimates of the contribution of HAP to AAP at global and regional scales.

### 4.1. Global

We examined nine bottom-up modeling studies that conducted global analyses of source attribution: Chafe 2014 (Chafe et al., 2014), Lelieveld 2015 (Lelieveld et al., 2015), Butt2016 (Butt et al., 2016), Silva 2016 (Silva et al., 2016), Karagulian 2017 (Karagulian et al., 2017), Weagle 2018 (Weagle et al., 2018), Crippa 2019 (Crippa et al., 2019), McDuffie 2021 (McDuffie et al., 2021) and Chowdhury 2022 (Chowdhury et al., 2022). The studies and their features are listed in Table 1. Overall, results from these nine studies suggest that HAP globally contributes 12–30 % of ambient PM2.5 concentrations (Fig. 1).

Comparing estimates from these studies is challenging, as each has unique model configurations and inputs. The estimates presented by each study would likely vary if the input data were changed relative to the configuration used originally. These uncertainties are not presented to undermine the utility or value of these models or their findings. Rather, we emphasize the relative consistency of estimates given the heterogeneity in emissions inventories, time scales, and geographic resolutions evaluated.

Differences between the studies are substantial. The simulation year (the base year for which the estimations were made) spans the last two decades. Models vary widely in complexity (i.e. Chafe2014 used a reduced complexity air pollution model, the TM5-FASST while Lelieveld 2015 used a detailed atmospheric chemistry model). Different emissions inventories were also used, with different characterizations of what falls into ‘residential’ emissions. Additionally, some studies included natural and anthropogenic emissions. These include biogenic emissions from vegetation and forests, and natural emissions like volcanic SO2, wind-driven dust, and sea salt.

Across the studies reviewed here, 0.5–1.35 million deaths yearly are associated with HAP’s contribution to AAP. Even the lowest estimate of the contribution of HAP to AAP (i.e., Chafe2014) indicates that household sources contribute a significant portion of the large mortality burden due to ambient PM2.5 exposure. However, attributing mortality to HAP’s contribution to AAP depends on multiple factors, including the exposure response function used, baseline mortality rates, and the fraction population exposed for the simulation year. Chowdhury2022 performed a sensitivity study by adjusting PM by its toxicity and assigned higher toxicity to anthropogenic secondary organic carbon, primary organic carbon, and black carbon emissions and found that HAP’s contribution to excess deaths from AAP exposure increased by 60 % as use of solid fuels for household activities is a major emitter of these organic compounds. The latest GBD study (Murray et al., 2020) estimated 3.83 (2.72–4.97) million deaths from exposure to ambient PM2.5. Taking the median estimate of 20 % from the nine studies assessed here, 0.77(0.54–1) million premature deaths annually may be averted if HAP is completely mitigated (Fig. 2).

### 4.2. Regions

Regional models and local emission inventories are often optimized to suit local conditions better. Below, the impact of HAP on ambient PM2.5 is discussed for six major regions: East Asia, South Asia, Europe, Africa, Latin America, and North America. We compare global model estimates with those from each respective region and discuss strengths and weaknesses of both approaches.

#### 4.2.1. East Asia

In China, fossil fuel combustion from industries and power generation is the largest source of PM2.5 exposure (Pui et al., 2014; Zhang et al., 2019; Zheng et al., 2021). Although a series of effective actions have been taken in China to mitigate air pollution since 2014 (Lu et al., 2020; Silver et al., 2018; Zhang et al., 2019), mediating residential emissions was not targeted until recently (Lu et al., 2020; Meng et al., 2019). Over the last five years, many households in China have switched from use of coal in households for cooking to cleaner fuels (e.g., natural gas, liquified petroleum gas, biogas, and electricity). A recent study found that the consumption of wood and crop residues in rural China decreased by 63 % and 51 %, respectively from 1992 to 2012 but solid fuels remain a dominant energy source for heating, especially in Northern China (Tao et al., 2018).

We identified nine bottom-up global modeling studies (Table S1) and eight (Aunan et al., 2018; GBD MAPS working group, 2016; Liu et al., 2016; Reddington et al., 2019; Shen et al., 2019; Timmermans et al., 2017; Yun et al., 2020; Zhao et al., 2018) regional modeling studies (Table S1) that report the contribution of HAP to ambient PM2.5 over China. Regional studies are mostly lacking over the rest of East Asia (e.g., Japan, South Korea, and Taiwan). Due to the large volume of pollutants transported from the Chinese mainland to these countries (Kim, 2019; Yim et al., 2019), it is challenging to separate signals from transboundary pollution originating in China with those emitted regionally within these countries. Global modeling studies (e.g. Chowdhury2022) find that power generation and industrial emissions contribute ~ 50 % of total PM2.5 in South Korea and Japan with relatively low contribution from HAP (less than10 %). In South Korea and China, residential emissions increased from 2005 to 2010 followed by decreases after 2014, whereas in Japan there has been a continued decrease in residential emissions since the early 2000 s (Li et al., 2017).

**Fig. 3.**
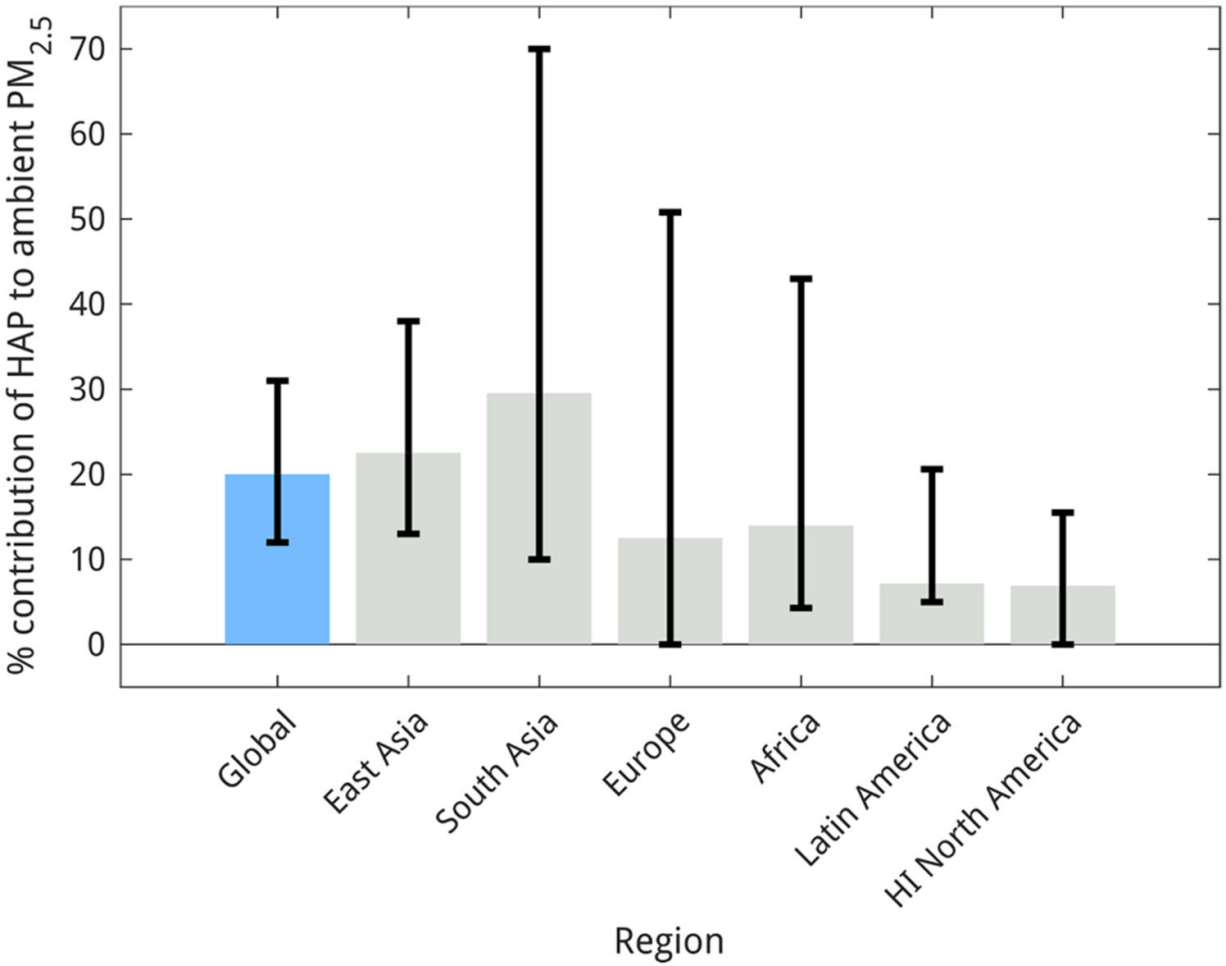
The percentage of population-weighted exposure to ambient PM2.5 due to solid fuel use in households. The bars indicate the median from the studies reviewed here, the whiskers range across the highest and lowest estimates.

Among the eight regional studies, three studies (Aunan et al., 2018; Yun et al., 2020; Zhao et al., 2018) reported the contribution of HAP to total PM2.5 exposure with the formulation of integrated population-weighted exposure (IPWE) to PM2.5 (Aunan et al., 2018). IPWE is defined as the weighted sum of PM2.5 concentrations in all microenvironments where people spend time, including the living room, bedroom, kitchen, and outdoors; it represents the total population-weighted exposure to PM2.5 through both ambient PM2.5 and HAP. Reasonably, the contribution of HAP to total IPWE was found to be considerably higher (Table S1) than the contribution of HAP to ambient PM2.5, though (Zhao et al., 2018) found that the contribution of HAP to IPWE decreased by 50 % from 2005 to 2015, presumably because the population-weighted HAP exposure decreased by ~ 56 % (56 % in urban and 45 % in rural areas) during the same time period. Results from the regional studies suggest a median contribution of 20.5 % (10–38 %), while the results from the seven global studies suggest a median contribution of 23 (13–32)% from HAP to ambient PM2.5 in East Asia (Fig. 3). These findings overlap with results from the top-down studies (Hopke et al., 2020; Lin et al., 2018). About 246 top-down studies on average report a 10.3 % contribution from biomass burning (agricultural and residential) to ambient PM2.5 in China, while 27 studies performed in the rest of East Asia report an average contribution of 10 % (Hopke et al., 2020).

The GBD (Murray et al., 2020) estimated 1.4 (1.1–1.7) and 0.36 (0.18–0.69) million premature deaths in East Asia from exposure to ambient PM2.5 and HAP respectively. Household solid fuel combustion, of both coal and biomass, is an important source of disease burden in China. Yun et al., (2020) quantified that 7.5 % of energy use in the residential sector is responsible for about 67 % of PM2.5 related premature deaths and approximately 80 % of these premature deaths occur among rural residents (Zhao et al., 2018). Coal burning in industries, power plants, and for domestic purposes contribute to more than 40 % of the total PM2.5 in China (Ma et al., 2017). GBDMAPS China (GBD MAPS working group, 2016) found that, in 2013, domestic biomass and coal combustion were responsible for 0.17 million deaths in China, 15 % larger than that of industrial coal, 30 % higher than transportation, and double that from coal fired power plants. Although biomass fuels are extensively used in the rural residential sector, adverse impacts on air quality and health associated with biomass use have historically been overlooked in comparison with coal (Meng et al., 2019; Yun et al., 2020) due to its association with severe pollution episodes in northern China. Shen et al., (2019) found that between 2005 and 2015, when biomass consumption and urban coal consumption dropped by 58 % and coal combustion decreased by 5 % overall, premature death from the residential sector’s contribution to ambient PM2.5 decreased by more than 60 %. Rapid urbanization and population migration, resulting in better access to cleaner fuels, improved income, and which makes cleaner fuels more affordable are the major reasons behind the decrease (Aunan and Wang, 2014). Despite recent trends of decreasing solid fuel use in households in China, the most recent study reviewed here, (McDuffie 2021), estimates 0.18 million premature deaths from the contribution of HAP to ambient PM2.5 in China, a majority of which may be avoided by an accelerated and complete transition to the use of cleaner fuels in households.

#### 4.2.2. South Asia

South Asian countries have some of the highest levels of ambient PM2.5 exposure in the world; about 97 % of the South Asian population is estimated to live in areas where the previous World Health Organization (WHO) Air Quality Guideline of 10 μg/m3 is exceeded (Chowdhury and Dey, 2016; David et al., 2019; Ravishankara et al., 2020). The leading contributors to ambient PM2.5 exposure in South Asia are sources associated with combustion of biomass and coal and other human activities that generate dust (MAPS Working Group, 2018). In addition, a large fraction of ambient PM2.5 in South Asia may be attributed to natural and aeolian dust (Chowdhury et al., 2022; MAPS Working Group, 2018). It should be noted that like in East Asia, there are large spatial heterogeneities in sources of PM2.5 in South Asia; however, residential use of solid fuel has been identified as the largest contributor in the most populous stretches of South Asia (Chowdhury et al., 2019b; Upadhyay et al., 2018). Though wood is the most prevalently used solid fuel in South Asia, dung cakes, crop residues and charcoal are also used (Abbas et al., 2020). The regional authorities have initiated policies to replace these solid fuels in households with cleaner fuels (e.g. the Indian government launched Pradhan Mantri Ujjwala Yojana (PMUY) to provide subsidized LPG connections to women in poor households). However as of 2019, in India, Pakistan, and Bangladesh, the three most populous countries of South Asia, about 61 %, 53 % and 76 % of the population still cook with solid fuels in spite of widespread efforts to promote cleaner fuels (Jeuland et al., 2015; Kar et al., 2019; Mani et al., 2020; Pillarisetti et al., 2022; Smith, 2018).

Several studies have established household use of solid fuels as the largest source of ambient PM2.5 in South Asia. Nine of the global bottom-up studies also reported the contribution of HAP to ambient PM2.5 exposure in South Asia. We also found six (Chowdhury et al., 2019b; Conibear et al., 2018; Guo et al., 2017; MAPS Working Group, 2018; Sharma et al., 2018; Upadhyay et al., 2018) regional modeling studies for India (see Table S2 for a detailed overview). From the global studies, we find that HAP causes at least 18 % and as much as 50 % of ambient PM2.5 in South Asia (Table S2). The median contribution of HAP emissions to AAP is 29.5 %. Our estimates coincide with a previous study that reviewed seven global and regional modeling studies (Chowdhury et al., 2019a). We find HAP’s contribution to ambient PM2.5 exposure in South Asia to be about 60 % higher than emissions from industries and coal-fed power plants, at least four times higher than open burning, and over 10 times higher than from transportation. 46 top-down studies carried out in South Asian countries report an average 15 % contribution of biomass burning (agricultural and residential) to ambient PM2.5 (Hopke et al., 2020).

Unlike in high-income western countries, PM2.5 pollution is not only an urban problem in India. Urban and nonurban areas have similar PM2.5 levels and health impacts (Chowdhury et al., 2019b; Ravishankara et al., 2020). Rather, the urban agglomerations may be impacted by transport of PM2.5 pollution from surrounding rural areas. It should be noted that the emissions in rural areas of northern India are at least a factor of 2 higher than in other regions in India, which may be attributed to the high prevalence of solid fuel use for household activities (Ravishankara et al., 2020; Venkataraman et al., 2020). Regional studies are yet to be performed in other countries of South Asia; however, global modeling studies (Lelieveld2015, Chowdhury2022, McDuffie2021) suggest that HAP may be responsible for 40–55 % and 25–31 % of ambient PM2.5 in Bangladesh and Pakistan, respectively.

**Fig. 4.**
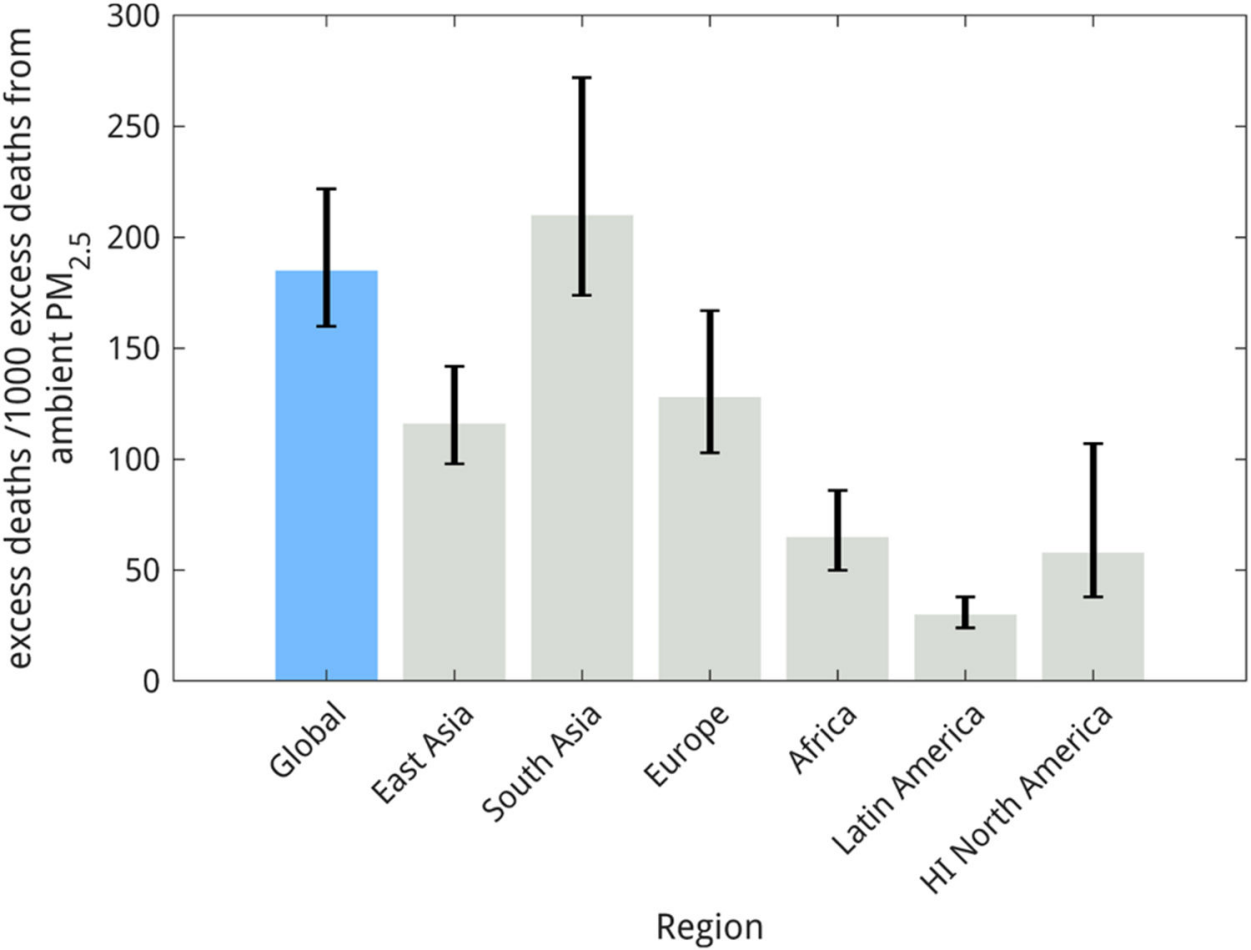
Excess deaths from contribution of household air pollution to ambient PM2.5 per 1000 excess deaths from ambient PM2.5 exposure, globally and in 6 major super regions.The bars indicate the median derived from the studies reviewed here, the whiskers range across the highest and lowest estimates.

The GBD (Murray et al., 2020) estimated 1.18 (0.91–1.43) and 0.84 (0.55–1.16) million premature deaths in South Asia from exposure to ambient PM2.5 and HAP respectively. The large contribution from residential solid fuel combustion to AAP exposure makes these fuels the dominant contributor to all air pollution related premature mortality in South Asia, except in mega-cities where emissions from fossil fuel burning sources mostly dominate. Across studies, a median estimate of 0.25 million excess deaths may be averted in the region if cleaner alternatives are used for household activities (Fig. 4). These findings emphasize the urgency of formulating extensive policies to reduce HAP in South Asia. Starting in 2016, the Government of India (Ministry of Petroleum and Natural Gas Government of India, 2016; Ministry of Power Government of India, 2014) embarked upon an ambitious program to tackle HAP, promoting use of liquefied petroleum gas (LPG) for cooking, but additional policies to ensure fuels are accessible and affordable and to account for other household activities, like heating needs, are also essential. In Nepal, where 69 % of the population still use solid fuels, the government, local bodies and stakeholders are embarking upon multiple efforts to help households transition to clean fuels with a target to completely shift to clean energy by 2030 (Das et al., 2021; Paudel et al., 2021). More than 90 % of households in most districts of Bangladesh use solid fuels, except around Dhaka, where less than a quarter of the households in three districts used solid fuel for cooking. Though the Improved Cookstove Programme in Bangladesh has helped to install 1.6 million modern, cleaner, and more efficient cookstoves, tackling the HAP problem in Bangladesh demands further interventions (Bangladesh Country Action Plan, 2013; Khan et al., 2017). Apart from supplying improved stoves and clean fuels, additional effort is also required in the South Asian countries to promote their sustained and near-exclusive use.

#### 4.2.3. Europe

Fossil fuel use in transport, power plants, and industries is often perceived as the largest sources of ambient PM2.5 and greenhouse gasses in European countries, especially in high-income Western Europe (Lelieveld et al., 2019; Thunis et al., 2019, 2018). However, due to very strict regulations, efficient end of the pipe controls, efficient combustion technologies in vehicles, power plants, and industries are applied (“EU Clean Air Policy, 2011,”; “Health aspects of air pollution and review of EU policies,”; WHO Regional Office for Europe, 2013). About 65 % of the total energy used by European households is required for space heating; given the ambitious European Union targets for replacing coal for heating, there has been a resurgence of wood as a heating fuel (Capros et al., 2016, 2013; Clean Heat, 2016). More than 70 million solid fuel appliances in Europe are technologically outdated, especially stoves and other single-room appliances that use wood inefficiently and contribute disproportionately to the overall emissions (Clean Heat, 2016). Despite the small share of total energy consumption, residential biomass and coal burning caused 46 % of total primary emissions of PM2.5 in Europe (biomass burning 36 % and coal burning 10 %), which is about 2 times higher than emissions from the transportation sector (Capros et al., 2016; Crippa et al., 2018; Denier van der Gon et al., 2015; Hendriks et al., 2015; Kranenburg et al., 2013; Kuenen et al., 2014).

Multiple studies have established that burning of solid fuel in European households is a big source of ambient PM2.5; it is primarily used for heating (Clean Heat, 2016; Denier van der Gon et al., 2015). As such, there is strong seasonality and regional heterogeneity depending on fuel used for heating and available economic resources to use cleaner fuels or to combust biomass more cleanly. We identified eight global studies (mentioned in Section 4.2) and five regional modeling studies (Hendriks et al., 2015; Karamchandani et al., 2017; Kukkonen et al., 2020; Pirovano et al., 2015; Thunis et al., 2018) that estimated the contribution of HAP to ambient PM2.5 in Europe (see Table S3 for a detailed overview).

From the global studies, we find that HAP may contribute to at least 5 % (in Western Europe) and as much as 40 % (in Central and Eastern Europe) of ambient PM2.5 (see Table S4). We estimated a median contribution of 15 % to ambient PM2.5 from solid fuel use in Europe (Fig. 3). Chafe2014 estimated zero contribution from cooking with solid fuels to ambient PM2.5 in Europe. Of the five regional studies, two (Karamchandani et al., 2017; Thunis et al., 2018) focused on major cities in Europe. Studying 150 major urban areas in Europe, Thunis et al., (2018) found that the average contribution from the residential sector in these urban areas to be 13 %. The largest contributions were estimated in urban areas of Central and Eastern Europe (e.g., in the Polish cities of Warsaw, Krakow and Katowice) where 48, 41 and 40 %, respectively, of ambient PM2.5 was estimated to come from use of solid fuels in households. In Western European cities (e.g., cities in Germany, UK, Belgium, and Scandinavian countries), HAP is a minor (less than5%) contributor. Karamchandani et al., (2017) studied 16 major cities from different geographies in Europe and found that, in winter, biomass use is the largest contributor to ambient PM2.5 in eleven of the sixteen cities. In summer, the study found a minimal contribution (less than5%) from biomass use to ambient PM2.5 in all cities except Oslo (11 %). (Pirovano et al., 2015) also found that the winter time contribution of HAP to ambient PM2.5 is at least 10 times higher than during summer in the Lombardy region of Italy. Wood burning for household heating was also found to be a large contributor to ambient PM2.5 in Scandinavia (Kukkonen et al., 2020; Orru et al., 2022; Savolahti et al., 2019). However, if annual averaged numbers are considered, residential solid fuel use is the smallest source identified in Europe, the contribution to ambient PM2.5 being lower by 43 %, 35 %, 32 % and 7 % compared to agricultural emissions, industry, natural sources, and transportation respectively. There have been 20, 8, 86 and 60 top-down studies which reported on average a 17.8 %, 15.4 %, 10.7 % and 12 % contribution of biomass burning (agricultural and residential) to overall particulate air pollution in Eastern, Northern, Southern, and Western Europe respectively (Hopke et al., 2020).

The GBD (Murray et al., 2020) estimated 0.39 (0.3–0.48) and 0.02 (0.007–0.05) million premature deaths in Europe from exposure to ambient PM2.5 and HAP respectively, out of which 56 % and 81 % of deaths from ambient PM2.5 and HAP respectively occur in Eastern and Central European countries. Across studies, a median estimate of 0.05 million premature deaths may be averted if cleaner alternatives are used for household heating in Europe, which translates to 128 (103–167) deaths per 1000 excess deaths from ambient air pollution (Fig. 4). Multiple country governments have initiated policies to implement strict emission standards for household combustion appliances. For example, since October 2010, the Aachen fuel ordinance in Germany (Amann et al., 2018) sets stringent emission limits for solid fuel space heaters with a nominal heat output from 4 to 15 kW, with new stoves limited to 40 mg/m^3^ PM and 1,250 mg/m^3^ CO, while old existing stoves have a limit of 75 mg/m^3^ PM and 2,000 mg/m^3^ CO. All existing stoves exceeding the emission limits had to be replaced or retrofitted by 2014. Some country governments also offer financial benefits, e.g. The Czech Ministry of the Environment (Amann et al., 2018; Czech Ministry of Environment, 2019) offers approximately 340 million euro to subsidize households and encourage them to replace old solid fuel boilers with environmentally friendly equipment. Austria also offers subsidies for installation of energy-efficient, climate- and environmental-friendly technology as well as energy-efficient buildings (Amann et al., 2018). In Norway, certain municipalities have subsidized the replacement of old stoves for new, clean-burning appliances (Lopez-Aparicio and Grythe, 2020). Restriction on solid fuel use has also been implemented in Lombardy, Italy where during the winter months the use of wood-burning fireplaces and stoves with less than 63 % approved efficiency is prohibited and is under the vigilance of local police (Amann et al., 2018). Ireland and Poland have also prohibited sale and use of low quality bituminous coal for household heating (Amann et al., 2018; Cofala and Klimont, 2012; Pietrzak et al., 2021). As stringent policies that restrict the use of solid fuels for household heating are imperative, current progress in Europe on tackling HAP with the development of innovative and low-emission wood burning appliances and strict emission standards is exemplary and the contribution of HAP to ambient PM2.5 is expected to decrease in next decades.

#### 4.2.4. Africa

Air pollution is a growing challenge for Africa (Abera et al., 2021; Makoni, 2020; Schwela, 2012). Economic advancement and increases in population and consumption have potentially resulted in an increase of anthropogenic pollution over sub-Saharan Africa (Abera et al., 2021; Hammer et al., 2020; Shaddick et al., 2020, 2018). Further, Africa’s population is expected to double by 2050; a large fraction of this increase is expected in major cities, potentially resulting in more traffic, expanded industry, and growth in other polluting sectors (KC and Lutz, 2014; Lutz et al., 2016). The rates of economic growth in some African countries (e.g., Nigeria) is similar to that of China and India in the last decade, when air pollution problems emerged in these two countries (Rees et al., 2019). Major anthropogenic sources of PM2.5 in sub-Saharan Africa include crop burning, road transportation, power production (mostly gasoline and diesel generators, and others), industry, waste burning, and road dust in addition to burning of solid fuels in households (Chowdhury et al., 2022; HEI, 2019). Natural and wind-blown dust dominates in Northern Africa, while biogenic secondary organic aerosols (SOA) and forest fires are dominant in sub-Saharan Africa. Within the African continent, meteorological factors also significantly influence air quality: dust from the Sahara is transported south during Harmattan (November end to mid-March) and heat waves that result in forest fires during August and September impact sub-Saharan Africa (De Longueville et al., 2010; Okeahialam, 2016). Further compounding the problem is the high fraction of the population using solid fuels for household activities in most of Africa (Bonjour et al., 2013). More than 90 % of households in sub-Saharan countries depend on solid fuels for cooking and other household activities. This activity is responsible for more than 70 % of the total primary anthropogenic PM2.5 emissions in Africa (Crippa et al., 2018; McDuffie et al., 2020).

We did not find any bottom-up regional modeling studies over the region beyond the nine global studies identified in Section 4.2 and Table S4. The nine global studies report a median contribution of 15 % from HAP to ambient PM2.5 (Fig. 3). All studies agree that HAP is the largest contributor to anthropogenic ambient PM2.5 in Africa contributing at least twice as much as industry, power generation, and transportation combined. Additionally, the production of charcoal, which is widely used for household activities contributes substantially to AAP (Bockarie et al., 2020). However, natural and wind-blown dust and forest fires are the largest contributors to ambient PM2.5 in Africa. The average contribution of biomass burning (agricultural, wildfire and residential) to ambient PM2.5, from 10 top-down modeling studies mostly carried out in Western sub-Saharan Africa, was estimated to be 3 %, which is considerably lower than the findings from the bottom-up studies (Hopke et al., 2020).

The GBD (Murray et al., 2020) estimated 383 (288–419) and 696 (525–878) thousand premature deaths in Africa from exposure to ambient PM2.5 and HAP, respectively. Across the nine global bottom-up studies, a median estimate of 25,000 excess deaths, or 65 (50–86) deaths per 1000 excess deaths from AAP were attributed to HAP (Fig. 4). While most sub-Saharan African countries acknowledge air pollution related to the unsustainable use of solid fuel in households, implementation of approaches to address clean cooking vary.

While LPG has a big market share in North African countries (greater than80 % of the population use LPG), in sub-Saharan Africa, its penetration is typically low (less than10 %), with some exceptions. In Senegal, subsidies on LPG and availability in small affordable units enabled accessibility among lower and middle class households (Ekouevi and Tuntivate, 2012). The LPG promotion program resulted in 500-fold growth in LPG consumption from 1974 to 2012, though the subsidies were withdrawn in 2000 (Laan et al., 2010). The National LPG Policy in Ghana of 2017 established a roadmap towards achieving at least a 50 % penetration of LPG by 2030. As of November 2017, this led to the dissemination of LPG cookstoves to 149,500 rural households (HEI, 2019; Multiconsult, 2020).The Government of Tanzania also targets clean cooking solutions to rural households, with a goal of 75 % of the population using either biogas, LPG, ethanol, natural gas, or charcoal in improved cookstoves by 2030 (Multiconsult, 2020). This led to a 20 % increase of LPG imports in 2019 compared to the previous year (Doggart et al., 2020). Likewise, the government of Cameroon in 2016 announced a Master Plan for an energy transition in the household sector. The target is that by 2030, 58 % of the population will use LPG as cooking fuel, compared with less than 20 % in 2014 (Kypridemos et al., 2020).

Africa is projected to be home to at least 40 % of the global population of children less than 5 years of age by 2050 (KC and Lutz, 2014; Riahi et al., 2017). Currently 30 % of the total mortality in Africa associated with PM2.5 occurs among children; the rate has been increasing over the last decade(Murray et al., 2020). Extensive action is needed to tackle solid fuel use in households.

#### 4.2.5. Latin America

Exposure to AAP and HAP is one of the most persistent environmental risk factors in Latin America (Goldemberg et al., 2004; Romieu et al., 1990). Steady urbanization, growing industrialization, vehicular emissions, and power plants are the primary sources of air pollution in urban areas, where more than 80 % of the population resides (Molina et al., 2015; Pauchard and Barbosa, 2013). Frequent forest fires during the dry season of July-October also contribute largely to air pollution problems in the region (Ballesteros-González et al., 2020; Mendez-Espinosa et al., 2019). In addition, about 80–160 million people in Latin America are exposed to pollution from household solid fuel use. In Central American countries like Nicaragua, Guatemala, and Haiti, between 50 and 90 % of the population use solid fuels for cooking. In Brazil, Argentina, Chile, Ecuador, and Guyana, there has been a notable shift towards clean fuels over the last few decades - around 90 % of households in these countries now have access to non-solid fuels (Coelho et al., 2018; Goldemberg et al., 2018). Nonetheless, the problem of HAP still persists in the region, with a significant contribution to ambient PM2.5 exposure (Johnson et al., 2020, 2020; Pillarisetti et al., 2022, 2019).

Aside from the nine global modeling studies, we did not find any bottom-up regional modeling studies for the region (see Table S5 for a detailed overview). All nine studies agree that HAP is a moderate contributor to ambient PM2.5 in Latin America, being at least 60 %, 40 % and 10 % lower than the contributions from forest fires, industries, and traffic, respectively, but twice the contribution from power plants. The average contribution of biomass burning (agricultural, wildfire and residential) to ambient PM2.5, from 7 and 4 top-down modeling studies carried out in Brazil and the rest of Latin America were estimated to be 22.4 and 12.2 % respectively (Hopke et al., 2020).

About 0.15 (0.11–0.19) and 0.06 (0.03–0.08) million annual premature deaths were attributed to exposure to ambient PM2.5 and HAP respectively in 2019 (Murray et al., 2020). Across the nine global bottom-up studies, a median estimate of 4500 excess deaths could be attributed to the contribution of HAP to ambient PM2.5 in Latin America (Fig. 4). Over the last 3 decades, solid fuel use has decreased significantly in Latin America, resulting in a 56 % decrease in excess deaths from HAP exposure, while the population increased by at least 50 % during the same period. Multiple successful mitigation policies and campaigns that introduced efficient woodfuel cookstoves and clean fuels may be credited for the benefit. Several organizations designed and distributed improved cookstoves in Latin America, some of which were successfully adopted by targeted communities (Coelho et al., 2018; Tagle et al., 2019). With much of the population migrating to live in urban areas and governmental interventions to regulate price and use of LPG, along with fuel subsidy provision, there has been an expansion in LPG usage (more than 70 % of the Latin American population already uses LPG for cooking). For example, in Brazil in the 1940 s, only a few thousand households had access to clean fuels, while in 2019, ~90 % of the population has access to clean fuels, which was possible due to subsidies from the government (also note ~ 90 % of Brazilians live in cities now compared to 25 % in the 1940 s, which led to increased access to clean fuels (Goldemberg et al., 2004). However, it must be noted that excess mortality from ambient PM2.5 exposure increased by 70 % from 1990 to 2019, indicating the need for implementation of mitigation policies, including completely displacing solid fuel usage.

#### 4.2.6. High-income North America

In the 1950 s and 1960 s, the United States of America (USA) experienced heavy pollution episodes originating from rapidly growing industries, unclean fuel used in vehicles, coal-fed power plants, and use of coal and wood in households for heating (Fowler et al., 2020; Haagen-Smit, 1952). Over the last few decades, several approaches were taken to improve air quality. The Clean Air Act of 1970 resulted in comprehensive air quality management plans (Ross et al., 2012; US EPA, 2013), leading to visible improvement of air quality across the country, with pollution levels decreasing significantly over the last decades. The government of Canada has also been taking action to reduce levels of air pollution through the Canadian Environmental Protection Act of 1999, which regulates the amount of pollutants released into the air each year (Basu and Lanphear, 2019; Institute of Medicine (US) et al., 2007). As of 2019, less than 0.2 % of the households in North America use solid fuel for cooking; however, ~5% and 1.9 % of households in Canada and USA, respectively, depend on burning wood for indoor heating.

In addition to the eight global modeling studies (listed in Section 4.2 and Table S6), we did not find any bottom-up regional modeling studies for North America. The median contribution of HAP to ambient PM2.5 from the global studies was estimated to be 6.9 % (Fig. 3). Chafe2014 found zero contribution from cooking with solid fuels to ambient PM2.5 in high income North America, which corroborates the propensity of use of solid fuels for heating in North America as compared to cooking, though a majority of the population (greater than95 %) uses oil and electricity for household heating. These studies agree that HAP is a minor contributor to ambient PM2.5 in high-income North America, being at least 70 %, 80 % and 85 % lower than the contributions from land traffic, agriculture and power generation respectively. The 23 and 148 top-down modeling studies (Hopke et al., 2020) in Canada and the United States of America found an average contribution of 13.8 % and 4.4 % respectively from biomass burning (agricultural, wildfire and residential), of which a major fraction may be attributed to the frequent wildfires (Aguilera et al., 2021; Chen et al., 2017).

The GBD (Murray et al., 2020) estimated 55 (27–77) and 0.2 (0.02–0.48) thousand annual premature deaths in high-income North America from exposure to ambient PM2.5 and HAP, respectively. Across the nine global bottom-up studies, a median estimate of 3,000 annual excess deaths were attributed to the contribution of HAP to ambient PM2.5. Nonetheless, there are ongoing efforts to curb emissions from burning of wood and coal for household heating because in certain areas, ambient air quality standards are exceeded mainly due to emissions from heating stoves. In March 2020, the United States Environmental Protection Agency (US EPA) issued final amendments to the New Source Performance Standards (NSPS) for residential wood heaters, residential hydronic heaters, and forced-air furnaces to further control emissions (US EPA, 2020). This rule amends the 2015 NSPS by removing certain minimum requirements for pellet fuels and clarifying a requirement regarding the use of unseasoned wood in pellet fuel production (US EPA, 2018). Considering the high prevalence of use of solid fuels for heating (~15 % and 30 % of population in Canada and USA respectively) in the 1950 s and 1960 s, the successful implementation of the Clean Air Act in the USA and the Environmental Protection Act in Canada might be an example to follow for some developing countries of South Asia and Africa.

## 5. Uncertainties and limitations.

The wide range of estimates (indicated by the length of the whiskers in Fig. 3) of the contribution of HAP to ambient PM2.5 both globally and regionally may be attributed to three major factors that also contribute to uncertainties in the studies discussed here: a) choice of emission inventories, b) bottom-up model configuration and c) health impact assessment methodology. We acknowledge that all the studies considered in this review have inherent uncertainties, which have been explicitly discussed in the original studies.

The selection of an emissions inventory plays a major role in determining how accurately models simulate ambient pollutant concentrations. The emission inventories used by the studies reviewed here are tabularized in Table 1 and Tables S1-S6. Use of inventories which fail to incorporate fine details (e.g. type and amount of solid fuel used in each household by region and end use, for instance wood use in water heating stoves among rural households in a state of India) results in uncertainty. Potential uncertainties in emission factors, unexpected sources (such as trash burning), inclusion of evolving emission standards, and knowledge of task-specific fuel type, among others, makes formulating an accurate emission inventory a daunting task. For a specific region of interest, it is expected that emission inventories designed locally (Sadavarte and Venkataraman, 2014; Tong et al., 2020; Venkataraman et al., 2020; Zheng et al., 2021) incorporate finer detail than global emission inventories. For example, recent Indian inventories (Sadavarte and Venkataraman, 2014; Venkataraman et al., 2020) take into account space and water heating behaviors observed in parts of India where it has been assumed non-existent in other inventories (e.g., Emissions Database for Global Atmospheric Research or EDGAR). A recent study (Saikawa et al., 2017) found that large disagreements exist among the five inventories used for China at disaggregated levels. The study found that for the residential sector, estimates from local emission inventories were always higher than those from the global inventory. Different emission inventories also have distinct sector classification. For example, EDGAR and the Community Emissions Data System (CEDS) classify household solid fuel use under the umbrella of the ‘residential and commercial sector,’ which includes emissions from households and the commercial sector. These emission databases assume that such activities consume similar fuels for similar purposes and use an Intergovernmental Panel on Climate Change code (https://www.ipcc-nggip.iges.or.jp/public/gl/invs1.html) for grouping the sectors. However, these inclusions may add ~ 5 % more emissions on top of those actually from households, more so in regions where diesel generator use is prevalent. Ideally, disaggregated emission data (i.e. household cooking and heating, diesel generators etc.) should be made available, though such is not the case currently. In contrast, emissions inventories which include only household activities, like cooking, water heating, space heating, and lighting are expected to provide a more accurate picture of the contribution of HAP to AAP (Chowdhury et al., 2019b). A detailed inter-emission inventory comparison may help better understand how inclusion of these additional sources (besides the conventional household sources) impact estimates of the contribution of household emissions to ambient air pollution.

Choices made in bottom-up models about spatial and temporal resolution, initial and boundary conditions for chemistry and aerosols if required (as in regional air pollution models), photo-chemical reactions included in the chemical mechanism, both for the gas and the aerosols phase, deposition processes; and reproduction of observed meteorology also plays a significant role in contributing to the considerable range in contribution of HAP to ambient PM2.5 among studies. Coarsely resolved global models (with grid boxes representing large areas), underestimate variability and concentrations of pollutants in the most polluted regions of the globe, which are also regions with prevalent household solid fuel use (Chowdhury et al., 2022; Lelieveld et al., 2015; McDuffie et al., 2021; Silva et al., 2016). Regional models (like used in Chowdhury et al., (2019b); Karamchandani et al., (2017) and Thunis et al., (2019)) meanwhile, are often better-suited to local conditions not captured by global models and thus provide more accurate estimates of exposures and sources (Feser et al., 2011). A regional model inter-comparison study for Europe (Prank et al., 2016) found notable differences between predictions of seasonal variations of particulate matter attributable to different emission inventories and aerosol processes. Conversely, global models capture long-range aerosol transport across regions better than regional models by virtue of their broad geographic extent. Models capable of performing simulations at finer resolutions in higher detail are computationally intense. Global models generally used to simulate at coarse resolutions are typically incapable of identifying local pollution hotspots (Butt et al., 2016; Silva et al., 2016). However, some studies (Chowdhury et al., 2022; Lelieveld et al., 2013) indicate that the choice of model resolution may not necessarily be the leading cause of uncertainty, especially in Europe and North America. Large model intercomparison initiatives are needed to detect the sensitivity of model inputs as in Prank et al., (2016). An alternative approach is generation of cumulative ensemble models leveraging the variability in estimated model outputs (Meehl et al., 2000).

Additional uncertainties may be associated with estimating excess deaths attributable to HAPs contribution to ambient PM2.5. Estimating excess deaths from ambient PM2.5 requires four major inputs: (1) the distribution of population-weighted exposures; (2) specification of a level of exposure below which no increased risk of mortality is assumed to exist (the counterfactual concentration); (3) estimates of the relative risk, obtained through exposure–response functions (ERFs); (4) estimates of baseline mortality rates and detailed demographic information (Burnett and Cohen, 2020; Chowdhury and Dey, 2016; Murray et al., 2020). Inherent uncertainties in each of these inputs may contribute to the range in estimates of excess deaths among studies. Uncertainties that may stem from the use of different inputs for estimating excess mortality is described elsewhere (Pozzer et al., 2023) and in SI Text 2.

It should be noted that we compiled data reported by studies performed in specific countries to produce regional estimates of contributions of HAP to ambient PM2.5 (Fig. 3), which might result in considerable bias for certain regions. However, considering that China and India houses 82 % and 70 % of the population of East Asia and South Asia respectively, using the contributions of HAP to ambient PM2.5 in China and India to estimate the median contribution of HAP to ambient PM2.5 in East and South Asia (Aunan et al., 2018; MAPS Working Group, 2018; Upadhyay et al., 2018; Venkataraman et al., 2018; Zhao et al., 2018) respectively may result in a lower bias than using the results from studies executed in few European cities for estimating the median contribution of HAP to ambient PM2.5 in Europe (Karamchandani et al., 2017; Thunis et al., 2018). Though, we acknowledge that fuel use patterns vary significantly also within South Asia and East Asia. For example, 36 % of the households in China use solid fuels while less than 1 % of the population in South Korea and Japan depend on solid fuels. However, a large fraction of air pollution over South Korea and Japan are transported from mainland China (Kim, 2019; Xie and Liao, 2022).

## 6. Conclusions and future directions

Twenty-two years into the twenty-first century, the oldest source of air pollution still prevails. In spite of the many policies implemented by national governments and promoted by global bodies, solid fuel remains by far the dominant fuel used for cooking in most LMICs of South Asia and Africa, while solid fuel (both biomass and coal) is used for heating in much of East and Central Europe and Northern China. Around 3 billion people world-wide still lack access to clean fuels to meet their household energy needs, and this group disproportionately suffers from ill health due to direct exposure to both HAP and ambient PM2.5.

In this report, we review bottom-up modeling studies and find that HAP is the dominant source of ambient PM2.5 globally regardless of variations in model types, configurations, and emission inventories used in the studies. All of them identify residential emissions as one of the leading contributors to ambient PM2.5 globally, contributing to about 20 (12–31)% of global PM2.5 exposure (Fig. 3). We find that this fraction is responsible for 0.77(0.54–1)million premature deaths from exposure to ambient PM2.5, which is in addition to 2.3 (1.6–3.1) million deaths from direct exposure to HAP (Murray et al., 2020).

Coordinated actions are required to avert this burden. One way is to promote campaigns encouraging the use of cleaner biomass cookstoves; however, solid biomass is a difficult fuel to burn efficiently. Reducing or replacing household solid fuel use with cleaner energy sources, like liquified petroleum gas (LPG), ethanol, or electricity, should be a high priority. Some countries like Brazil and India have substantially expanded access to LPG for cooking in their households. Expanding access to LPG to wider geographies in Africa, Latin America, and across South Asia should be coupled with efforts to identify other contributors to HAP, including household activities like water and space heating, kerosene lighting, and animal fodder preparation. In most of Europe, where there is a rise in the use of wood for heating (https://unece.org/climate-change/press/wood-energy-rise-europe), stricter measures should be enforced to replace outdated heating appliances with efficient and low emitting devices.

We suggest consistent and coordinated efforts in quantifying emissions from multiple household activities and integrating modeling techniques as a significant step towards building efficient mitigation policies to reduce and replace solid fuel use in the households. Given the heterogeneity in findings for the literature reviewed in this report, there is a strong need for future work along a range of axes, each of which are discussed in the section below.

### (a) Improving and integrating emission inventories

The studies reviewed here used a variety of emissions inventories with different classifications of the residential sector. Some inventories, like those used in Lelieveld2015, Butt2016, Silva2016, McDuffie2021, use the term ‘residential and commercial sector,’ which defines HAP emissions to be originating both from households as well as from the commercial sector (Crippa et al., 2018; Hoesly et al., 2018; McDuffie et al., 2020). Such emission inventories consider that both of these types of activities use similar fuels for similar purposes. These additional activities may add up to 5 % on top of the emissions from household use of solid fuels, depending on the geography (Chowdhury et al., 2019a). In such instances, it is difficult to differentiate these sources from HAP (Chowdhury et al., 2019a). This calls for dissemination of comprehensive emissions data that separates household emissions from commercial emissions, to the extent possible.

Global inventories are often not representative of regions for which there is limited energy end-use activity data. In fact, many sources dominant in LMICs (e.g., motorcycles, kerosene use, open waste burning, ad hoc oil refining, animal fodder preparation (Gerber et al., 2013)) are missing from the global emission inventories (Crippa et al., 2018; HEI, 2019; Marais and Wiedinmyer, 2016; Saikawa et al., 2017; Solazzo et al., 2021). There are also large disagreements between these inventories. The emission factors used to translate the amount of fuel burnt to pollution released are likely not representative of the inefficient and vastly heterogeneous combustion conditions in developing countries.

In Africa and South Asia, where currently there are large uncertainties in emission factors and multiple missing sources, future work should focus on utilizing satellite retrievals to build high temporal resolution emissions databases for gaseous species such as SO2, NO2 along with quantifying the primary emissions from missing sources. Considering that accurate emission inventories encompassing detailed sources are imperative for bottom-up modeling studies, we call for leveraging regional efforts to formulate emission inventories in data-sparse regions (in Africa and South America) and encourage a consortium of country specific efforts like the REAS/MEIC emission inventory of China, the National Emission Inventory for USA, Toegepast Natuurwetenschappelijk Onderzoek -MAAC (TNO-MAAC) for Europe, and the Speciated Multipollutant Generator (SMoG) for India. Constructing such emission inventories would provide opportunities for more regional modeling studies, particularly in regions where no regional bottom-up studies have been carried out till date to understand the impact of HAP to AAP in data-sparse regions. Efforts should be made to reconcile these regional inventories so that they can be better integrated with global inventories.

Inventories may also be improved with use of better data on primary and secondary fuel consumption, through the use of nationally representative surveys. These activities are being promoted globally through WHO’s efforts to harmonize household energy surveys. These modifications and detailed emission characterizations are expected to aid future bottom-up modeling studies to further classify the contributions of emissions from different household activities (e.g., heating, cooking, kerosene lighting, animal fodder preparation, other commercial activities) to ambient PM2.5 exposure and quantify their related health impacts.

We also suggest a detailed inter-emission inventory comparison study to understand the regional comparison (with observation and satellite information) and applicability of different emission inventories in different geographies, particularly in regions where there are no regional emission inventories available. However, it should be noted that emissions from simple household stoves are inherently more diverse and difficult to estimate due to the importance of e.g. fuel and stove characteristics, hence likely can not achieve as high a precision as emission factors for other sources such as power plants and industries (Shen et al., 2021).

### (b) Integrating bottom-up and top-down techniques

The contribution of HAP to ambient PM2.5, as discussed in previous sections, can be performed using both top-down (receptor-oriented) and bottom-up models (source-oriented). The outputs from top-down models usually reconstruct the measured PM concentrations through a number of source categories based on distinct chemical fingerprints, while atmospheric chemistry models calculate the PM concentration based on known source categories derived from the emission inventories. However, the comparison is not straightforward, e.g., the chemical fingerprints for household solid fuel burning are similar to forest fires, which may be concerning for regions where both are dominant, like in sub-Saharan Africa. Conversely, the bottom-up modeling studies cannot capture contributions from minor sources like kerosene lighting and motorbikes, if they are not specifically characterized in the emission inventories. The validation of any source-apportionment results is also critical as the true contribution of sources to the levels of one pollutant cannot be measured directly by either of these techniques.

Therefore we call for consolidation of top-down and bottom-up methodologies, such that they complement each other, which essentially requires simultaneous application of both top-down and bottom-up techniques, extensive availability of tools, data, expertise, substantial financial support and time. Targeted investments in enhanced air pollution monitoring and emissions measurements, coupled with refined bottom-up analyses, may capture the benefits of policy interventions to improve clean household energy and thus motivate sustained action. In data and monitoring limited regions, we suggest investments to enhance monitoring and top-down techniques which can inform and build indigenous emissions data, therefore refining bottom-up analyses. This will potentially generate the capacity to capture emissions from use of solid fuels in different household activities and identify any benefit of interventions. The evidence we present also creates an opportunity to strengthen policy measures taken across many low-and-middle income countries to mitigate HAP, such strategies are expected to benefit households relying on solid fuels and broader populations who would benefit from improved ambient air quality.

### (c) Assimilating the toxicity of species emitted from HAP

Although the biological mechanisms are not yet fully elucidated, evidence suggests that certain pro-oxidant chemical components of PM_2.5_, e.g. anthropogenic secondary organic aerosols (aSOA), black carbon (BC) and primary organic aerosols (POA) induce oxidative stress and inflammation, leading to respiratory and cardiovascular diseases (Bates et al., 2019; Daellenbach et al., 2020; Huang et al., 2012; Liu et al., 2014; Park et al., 2018). A recent study showed that aSOA has a significantly higher oxidative potential than biogenic SOA and secondary inorganic aerosols (Daellenbach et al., 2020), and other studies showed similar effects for BC and POA, which involve primary ultrafine combustion particles that can carry noxious species like polycyclic aromatic hydrocarbons (Bates et al., 2019; Niranjan and Thakur, 2017). Transition metals like soluble copper and iron, while generally present at much lower concentrations, may have relatively high oxidative potential compared to the organic species (Kajino et al., 2021; Park et al., 2018). While clinical studies on mechanisms and impacts of chronic exposure to components of PM_2.5_ are still sparse, toxicological and epidemiological findings suggest that associations between adverse health effects and exposure to PM_2.5_ are significantly stronger for anthropogenic carbonaceous components than total PM_2.5_ mass (Grahame et al., 2014; Janssen et al., 2011; Lippmann et al., 2013; Magalhaes et al., 2018). Household fuel combustion is a major source of carbonaceous aerosols including black carbon and organic compounds (precursors for secondary organic aerosols) which are much more hazardous to human health compared to secondary inorganic aerosols. If HAP emissions are indeed more toxic per unit mass inhaled than PM2.5 emissions from other sources, the health effects of air pollution originating in solid fuel using households may be even greater than those estimated in the studies discussed here. Chowdhury2022 (Chowdhury et al., 2022) indicated that if the anthropogenic organic aerosols are twice as toxic compared to other aerosols, the contribution of HAP emissions to AAP increases by 60 %. Therefore, to strengthen health impact-based policies, we recommend studies that may enhance the understanding of the mechanisms, magnitude of increased risks to health posed by black carbon and anthropogenic organic aerosols, their major sources (which might be drastically different from major sources of PM2.5) together with regulatory policies that aim at reducing the disease burden of ambient PM2.5.

## Supplementary Material

SI

## Figures and Tables

**Table 1:** Information about the global studies on contribution of HAP to ambient PM2.5 reviewed. Please see SI Text 2 to interpret how model configurations, choice of emission inventories and exposure response functions (ERFs) may influence the variability between the studies reviewed here.

Study name	Study year	Model Used	Meteorology	Horizontal resolution	Secondary particle formation	Emission Inventory	Anthropogenic Emission Sources	Residential Emission	% of ambient PM2.5 from HAP (Global)	Associated premature mortality (in million)	ERF used
Chafe 2014 (Chafe et al., 2014)	2010	TM5-FASST	ECMWF,2010	TM5-FASST regions	Yes	GAINS	industry, land transport, residential and commercial energy, power generation, biomass burning, agriculture	Cooking	12	0.37	IER
Lelieveld2015 (Lelieveld et al., 2015)	2010	ECHAM5/MESSy	ECMWF, 2010	1.1° x 1.1°	Yes	EDGAR,2010	industry, land transport, residential and commercial energy, power generation, biomass burning, agriculture	Space heating, cooking, emission from local and commercial energy use from small combustion sources, diesel generator sets	31	1	IER
Butt 2016 (Butt et al., 2016)	2000	GLOMAP	ECMWF, 2000	2.8° x 2.8°	Yes	Various sources	Energy sources and distribution, industry, land transport, maritime transport, residential and commercial, agricultural waste burning	Space heating, cooking, emission from local and commercial energy use from small combustion sources	~15	0.3	log-linear
Silva 2016 (Silva et al., 2016)	2005	MOZART-4	GEOS,2005	0.5° x 0.67°	Yes	RCP,2005	Residential and commercial, energy, industry, land transport, shipping and aviation	Space heating, cooking, emission from local and commercial energy use from small combustion sources	30	0.67	IER
Karagulian 2017 (Karagulian et al., 2017)	2010	TM5-FASST	TM5,2001	1°x1°	Yes	EDGAR-HTAP, 2010	Agricultural, power generation, industrial non power, residential energy use and land transport.	Small scale supplemental engines for residential, commercial, agricultural, solid waste and wastewater treatment plants, Cooking, space heating water heating, lighting	~20	NA	NA
Weagle 2018 ( Weagle et al., 2018)	2014	SPARTAN observations + GEOSChem	GEOS MERRA-2, 2014	2°×2.5°	Yes	EDGARv4.4, 2010	industry, land transport, residential and commercial energy, power generation, biomass burning, agriculture	Space heating, cooking, emission from local and commercial energy use from small combustion sources, diesel generator sets	21	NA	NA
Crippa 2019 (Crippa et al., 2019)	2010	TM5-FASST	TM5,2001	1°x1°	Yes	EDGAR-HTAP, 2010	Agricultural, power generation, industrial non power, residential energy use and land transport.	Small scale supplemental engines for residential, commercial, agricultural, solid waste and wastewater treatment plants, Cooking, space heating water heating, lighting	~20	0.42	IER
McDuffie2021 (McDuffie et al., 2021)	2017	GEOSChem, satellite	GEOS,2017	2° × 2.5° globally, 0.5° × 0.625° over North America, Europe and Asia.	Yes	CEDS,2017	Agricultural soils, agricultural waste burning, residential, industry,power generation, ships, transportation	residential heating and cooking,commercial and institutional combustion, combustion from agriculture, forestry, and fishing	19.2	0.74	MR-BRT
Chowdhury 2022 (Chowdhury et al., 2022)	2015	ECHAM5/MESSy	ERA5	1.1° x 1.1°	Yes	CEDS,2014	Agricultural soils, agricultural waste burning, residential, industry,power generation, ships, transportation	residential heating and cooking,commercial and institutional combustion, combustion from agriculture, forestry, and fishing	20(32)^1^	0.84(1.35)	MR-BRT

1 The number in parenthesis indicates the contribution of HAP emissions to AAP considering the anthropogenic organic aerosols to be twice more toxic compared to the other aerosols.

## Data Availability

Data will be made available on request.

## References

[R1] Abbas K, Li S, Xu D, Baz K, Rakhmetova A, 2020. Do socioeconomic factors determine household multidimensional energy poverty? Empirical evidence from South Asia. Energy Policy 146, 111754. 10.1016/j.enpol.2020.111754.

[R2] Abera A, Friberg J, Isaxon C, Jerrett M, Malmqvist E, Sjöström C, Taj T, Vargas AM, 2021. Air Quality in Africa: Public Health Implications. Annu. Rev. Public Health 42, 193–210. 10.1146/annurev-publhealth-100119-113802. 33348996

[R3] Abulude F, B M, A AE, A OS, 2017. A Review on Top-Down and Bottom-Up Approaches for Air Pollution Studies. 10.20944/preprints201703.0014.v1.

[R4] Aguilera R, Corringham T, Gershunov A, Benmarhnia T, 2021. Wildfire smoke impacts respiratory health more than fine particles from other sources: observational evidence from Southern California. Nat. Commun 12, 1493. 10.1038/s41467-021-21708-0. 33674571 PMC7935892

[R5] Amann M, Cofala J, Klimont Z, Christian N, Wolfgang S, 2018. Measures to address air pollution from small combustion sources. IIASA.

[R6] Aunan K, Ma Q, Lund MT, Wang S, 2018. Population-weighted exposure to PM2.5 pollution in China: An integrated approach. Environ. Int 120, 111–120. 10.1016/j.envint.2018.07.042. 30077943

[R7] Aunan K, Wang S, 2014. Internal migration and urbanization in China: impacts on population exposure to household air pollution (2000–2010). Sci. Total Environ 481, 186–195. 10.1016/j.scitotenv.2014.02.073. 24598149

[R8] Balakrishnan K, Ghosh S, Ganguli B, Sambandam S, Bruce N, Barnes DF, Smith KR, 2013. State and national household concentrations of PM2.5 from solid cookfuel use: results from measurements and modeling in India for estimation of the global burden of disease. Environ. Health Glob. Access Sci. Source 12, 77. 10.1186/1476-069X-12-77. PMC3851863 24020494

[R9] Ballesteros-González K, Sullivan AP, Morales-Betancourt R, 2020. Estimating the air quality and health impacts of biomass burning in northern South America using a chemical transport model. Sci. Total Environ 739, 139755 10.1016/j.scitotenv.2020.139755. 32758934

[R10] Banerjee T, Murari V, Kumar M, Raju MP, 2015. Source apportionment of airborne particulates through receptor modeling: Indian scenario. Atmospheric Res. 164–165, 167–187. 10.1016/j.atmosres.2015.04.017.

[R11] Bangladesh Country Action Plan, 2013.

[R12] Basu N, Lanphear BP, 2019. The challenge of pollution and health in Canada. Can. J. Public Health Rev. Can. Santé Publique 110, 159–164. 10.17269/s41997-019-00175-7. PMC6964550 30719696

[R13] Bates J, Fang T, Verma V, Zeng L, Weber RJ, Tolbert PE, Abrams JY, Sarnat SE, Klein M, Mulholland JA, Russell AG, 2019. Review of Acellular Assays of Ambient Particulate Matter Oxidative Potential: Methods and Relationships with Composition, Sources, and Health Effects. Env. Sci Technol 53, 4003–4019. 10.1021/acs.est.8b03430. 30830764

[R14] Bey I, Jacob DJ, Yantosca RM, Logan JA, Field BD, Fiore AM, Li Q, Liu HY, Mickley LJ, Schultz MG, 2001. Global modeling of tropospheric chemistry with assimilated meteorology: Model description and evaluation. J. Geophys. Res. Atmospheres 106, 23073–23095. 10.1029/2001JD000807.

[R15] Bockarie AS, Marais EA, MacKenzie AR, 2020. Air Pollution and Climate Forcing of the Charcoal Industry in Africa. Environ. Sci. Technol 54, 13429–13438. 10.1021/acs.est.0c03754. 33086012

[R16] Bonjour S, Adair-Rohani H, Wolf J, Bruce NG, Mehta S, Prüss-Ustün A, Lahiff M, Rehfuess E. a., Mishra V, Smith KR, 2013. Solid fuel use for household cooking: Country and regional estimates for 1980-2010. Environ. Health Perspect 121, 784–790. 10.1289/ehp.1205987. 23674502 PMC3701999

[R17] Brasseur GP, Jacob DJ, 2017. Modeling of Atmospheric Chemistry. Cambridge University Press, Cambridge. 10.1017/9781316544754.

[R18] Burnett R, Cohen A, 2020. Relative Risk Functions for Estimating Excess Mortality Attributable to Outdoor PM2.5 Air Pollution: Evolution and State-of-the-Art. Atmosphere 11, 589. 10.3390/atmos11060589.

[R19] Butt EW, Rap A, Schmidt A, Scott CE, Pringle KJ, Reddington CL, Richards NAD, Woodhouse MT, Ramirez-Villegas J, Yang H, Vakkari V, Stone EA, Rupakheti M, Praveen PS, Van Zyl PG, Beukes JP, Josipovic M, Mitchell EJS, Sallu SM, Forster PM, Spracklen DV, 2016. The impact of residential combustion emissions on atmospheric aerosol, human health, and climate. Atmospheric Chem. Phys 16, 873–905. 10.5194/acp-16-873-2016.

[R20] Capros P, De Vita A, Tasios N, Papadopoulos D, Siskos P, Apostolaki E, Zampara M, Höglund-Isaksson L, Winiwarter W, Purohit P, Böttcher H, Frank S, Havlík P, Gusti M, Witzke H, Fragkiadakis K, 2013. EU Energy, Transport and GHG emissions -Trends to 2050, Reference scenario 2013. 10.2833/17897.

[R21] Capros P, De Vita A, Tasios N, Siskos P, Kannavou M, Petropoulos A, Evangelopoulou S, Zampara M, Papadopoulos D, Paroussos L, Fragiadakis K, Tsani S, Fragkos P, Kouvaritakis N, Höglund-Isaksson L, Winiwarter W, Purohit P, Gomez-Sanabria A, Frank S, Forsell N, Gusti M, Havlík P, Obersteiner M, Witzke HP, Kesting M, 2016. EU ENERGY, TRANSPORT AND GHG EMISSIONS - TRENDS TO 2050- reference scenario-2016.

[R22] Chafe Z. a, Brauer M, Klimont Z, Van Dingenen R, Mehta S, Rao S, Riahi K, Dentener F, Smith KR, 2014. Household cooking with solid fuels contributes to ambient PM2.5 air pollution and the burden of disease. Environ. Health Perspect 122, 1314–20. 10.1289/ehp.1206340. 25192243 PMC4256045

[R23] Chen J, Li C, Ristovski Z, Milic A, Gu Y, Islam MS, Wang S, Hao J, Zhang H, He C, Guo H, Fu H, Miljevic B, Morawska L, Thai P, Lam YF, Pereira G, Ding A, Huang X, Dumka UC, 2017. A review of biomass burning: Emissions and impacts on air quality, health and climate in China. Sci. Total Environ 579, 1000–1034. 10.1016/j.scitotenv.2016.11.025. 27908624

[R24] Chowdhury S, Dey S, 2016. Cause-speci fi c premature death from ambient PM 2. 5 exposure in India : Estimate adjusted for baseline mortality. Environ. Int 91, 283–290. 10.1016/j.envint.2016.03.004. 27063285

[R25] Chowdhury S, Dey S, Guttikunda S, Pillarisetti A, Smith KR, Girolamo LD, 2019b. Indian annual ambient air quality standard is achievable by completely mitigating emissions from household sources. Proc. Natl. Acad. Sci. U. S. A 166, 10711–10716. 10.1073/pnas.1900888116. PMC6561163 30988190

[R26] Chowdhury S, Chafe Z, Pillarisetti A, Lelieveld J, Guttikunda SK, Dey S, 2019a. The Contribution of Household Fuels to Ambient Air Pollution in India – A Comparison of Recent Estimates [WWW Document]. Collab. Clean Air Policy Cent. URL https://ccapc.org.in/policy-briefs/2019/5/30/the-contribution-of-household-fuels-to-ambient-air-pollution-in-india-a-comparison-of-recent-estimates (accessed 8.26.21).

[R27] Chowdhury S, Pozzer A, Haines A, Klingmüller K, Münzel T, Paasonen P, Sharma A, Venkataraman C, Lelieveld J, 2022. Global health burden of ambient PM2.5 and the contribution of anthropogenic black carbon and organic aerosols. Environ. Int 159, 107020 10.1016/j.envint.2021.107020. 34894485

[R28] Clappier A, Belis CA, Pernigotti D, Thunis P, 2017. Source apportionment and sensitivity analysis: two methodologies with two different purposes. Geosci. Model Dev 10, 4245–4256. 10.5194/gmd-10-4245-2017.

[R29] Clean Heat, 2016. Residential Wood Burning: Environmental Impact and Sustainable solutions. Deutsche Umwelthilfe and The Danish Ecological Council.

[R30] Coelho ST, Sanches-Pereira A, Tudeschini LG, Goldemberg J, 2018. The energy transition history of fuelwood replacement for liquefied petroleum gas in Brazilian households from 1920 to 2016. Energy Policy 123, 41–52. 10.1016/j.enpol.2018.08.041.

[R31] Cofala J, Klimont Z, 2012. Emissions from households and other small combustion sources and their reduction potential. IIASA.

[R32] Conibear L, Butt EW, Knote C, Arnold SR, Spracklen DV, 2018. Residential energy use emissions dominate health impacts from exposure to ambient particulate matter in India. Nat. Commun 9, 1–9. 10.1038/s41467-018-02986-7. 29434294 PMC5809377

[R33] Crippa M, Guizzardi D, Muntean M, Schaaf E, Dentener F, van Aardenne JA, Monni S, Doering U, Olivier JGJ, Pagliari V, Janssens-Maenhout G, 2018. Gridded emissions of air pollutants for the period 1970–2012 within EDGAR v4.3.2. Earth Syst Sci Data 10, 1987–2013. 10.5194/essd-10-1987-2018.

[R34] Crippa M, Janssens-Maenhout G, Guizzardi D, Van Dingenen R, Dentener F, 2019. Contribution and uncertainty of sectorial and regional emissions to regional and global PM2.5 health impacts. Atmospheric Chem. Phys 19, 5165–5186. 10.5194/acp-19-5165-2019.

[R35] Czech Ministry of Environment, 2019. National Energy Climate Plan of the Czech Republic.

[R36] Daellenbach KR, Uzu G, Jiang J, Cassagnes L-E, Leni Z, Vlachou A, Stefenelli G, Canonaco F, Weber S, Segers A, Kuenen JJP, Schaap M, Favez O, Albinet A, Aksoyoglu S, Dommen J, Baltensperger U, Geiser M, Haddad IE, Jaffrezo J-L, Prıfmmode\acutee\elseé\fivıfmmode\hato\elseô\fit, A.S.H., 2020. Sources of particulate-matter air pollution and its oxidative potential in Europe. Nature 587, 414–419. 10.1038/s41586-020-2902-8. 33208962

[R37] Das I, Lewis JJ, Ludolph R, Bertram M, Adair-Rohani H, Jeuland M, 2021. The benefits of action to reduce household air pollution (BAR-HAP) model: A new decision support tool. PLOS ONE 16, e0245729. 33481916 10.1371/journal.pone.0245729 PMC7822293

[R38] David LM, Ravishankara AR, Kodros JK, Pierce JR, Venkataraman C, Sadavarte P, 2019. Premature Mortality Due to PM2.5 Over India: Effect of Atmospheric Transport and Anthropogenic Emissions. GeoHealth 3, 2–10. 10.1029/2018GH000169. 32159019 PMC7007096

[R39] De Longueville F, Hountondji Y-C, Henry S, Ozer P, 2010. What do we know about effects of desert dust on air quality and human health in West Africa compared to other regions? Sci. Total Environ 409, 1–8. 10.1016/j.scitotenv.2010.09.025. 20934742

[R40] Denier van der Gon H. a. C., Bergström R, Fountoukis C, Johansson C, Pandis SN, Simpson D, Visschedijk AJH, 2015. Particulate emissions from residential wood combustion in Europe – revised estimates and an evaluation. Atmospheric Chem. Phys 15, 6503–6519. 10.5194/acp-15-6503-2015.

[R41] Doggart N, Ruhinduka R, Meshack CK, Ishengoma RC, Morgan-Brown T, Abdallah JM, Spracklen DV, Sallu SM, 2020. The influence of energy policy on charcoal consumption in urban households in Tanzania. Energy Sustain. Dev 57, 200–213. 10.1016/j.esd.2020.06.002.

[R42] Donnelly AA, Broderick BM, Misstear BD, 2015. The effect of long-range air mass transport pathways on PM10 and NO2 concentrations at urban and rural background sites in Ireland: Quantification using clustering techniques. J. Environ. Sci. Health Part A Tox. Hazard. Subst. Environ. Eng 50, 647–658. 10.1080/10934529.2015.1011955. 25901845

[R43] Ekouevi K, Tuntivate V, 2012. Household Energy Access for Cooking and Heating: Lessons Learned and the Way Forward (World Bank Publications). The World Bank.

[R44] EU Clean Air Policy, 2011 [WWW Document], n.d. URL https://ec.europa.eu/environment/air/clean_air/review.htm (accessed 8.29.21).

[R45] Feser F, Rockel B, von Storch H, Winterfeldt J, Zahn M, 2011. Regional Climate Models Add Value to Global Model Data: A Review and Selected Examples. Bull. Am. Meteorol. Soc 92, 1181–1192. 10.1175/2011BAMS3061.1.

[R46] Fowler D, Brimblecombe P, Burrows J, Heal MR, Grennfelt P, Stevenson DS, Jowett A, Nemitz E, Coyle M, Liu X, Chang Y, Fuller GW, Sutton MA, Klimont Z, Unsworth MH, Vieno M, 2020. A chronology of global air quality. Philos. Trans. R. Soc. Math. Phys. Eng. Sci 378, 20190314. 10.1098/rsta.2019.0314. PMC7536029 32981430

[R47] GBD MAPS working group, 2016. Burden of Disease Attributable to Coal-Burning and Other Air Pollution Sources in China.

[R48] Gerber PJ, Steinfeld H, Henderson B, Organization, (FAO) Food and Agriculture, Gerber PJ, 2013. Tackling climate change through livestock: A global assessment of emissions and mitigation opportunities. FAO, Rome.

[R49] Goldemberg J, Johansson TB, Reddy AKN, Williams RH, 2004. A global clean cooking fuel initiative. Energy Sustain. Dev 8, 5–12. 10.1016/S0973-0826(08)60462-7.

[R50] Goldemberg J, Martinez-Gomez J, Sagar A, Smith KR, 2018. Household air pollution, health, and climate change: cleaning the air. Environ. Res. Lett 13, 030201 10.1088/1748-9326/aaa49d.

[R51] Grahame TJ, Klemm R, Schlesinger RB, 2014. Public health and components of particulate matter: The changing assessment of black carbon. J Air Waste Manage Assoc 64, 620–660. 10.1080/10962247.2014.912692. 25039199

[R52] Guo H, Kota SH, Sahu SK, Hu J, Ying Q, Gao A, Zhang H, 2017. Source apportionment of PM2.5 in North India using source-oriented air quality models. Environ. Pollut 231, 426–436. 10.1016/j.envpol.2017.08.016. 28830016

[R53] Guttikunda S, 2009. Urban particulate pollution source apportionment: definition, methodology, and resources. www.sim-air.org.

[R54] Haagen-Smit AJ, 1952. Chemistry and Physiology of Los Angeles Smog. Ind. Eng. Chem 44, 1342–1346. 10.1021/ie50510a045.

[R55] Hammer MS, van Donkelaar A, Li C, Lyapustin A, Sayer AM, Hsu NC, Levy RC, Garay MJ, Kalashnikova OV, Kahn RA, Brauer M, Apte JS, Henze DK, Zhang L, Zhang Q, Ford B, Pierce JR, Martin RV, 2020. Global Estimates and Long-Term Trends of Fine Particulate Matter Concentrations (1998–2018). Env. Sci Technol 54, 7879–7890. 10.1021/acs.est.0c01764. 32491847

[R56] Health aspects of air pollution and review of EU policies: the REVIHAAP and HRAPIE projects [WWW Document], n.d. URL https://www.euro.who.int/en/health-topics/environment-and-health/air-quality/activities/health-aspects-of-air-pollution-and-review-of-eu-policies-the-revihaap-and-hrapie-projects (accessed 8.29.21).

[R57] HEI, 2018. State of Global Air Boston, MA.

[R58] HEI, 2019. Contribution of Household Air Pollution to Ambient Air Pollution in Ghana: Using Available Evidence to Prioritize Future Action.

[R59] Hendriks C, Kuenen J, Kranenburg R, Scholz Y, Schaap M, 2015. A shift in emission time profiles of fossil fuel combustion due to energy transitions impacts source receptor matrices for air quality. Environ. Sci. Process. Impacts 17, 510–524. 10.1039/C4EM00444B. 25594282

[R60] Herich H, Gianini MFD, Piot C, Močnik G, Jaffrezo J-L, Besombes J-L, Prévôt ASH, Hueglin C, 2014. Overview of the impact of wood burning emissions on carbonaceous aerosols and PM in large parts of the Alpine region. Atmos. Environ 89, 64–75. 10.1016/j.atmosenv.2014.02.008.

[R61] Hoesly RM, Smith SJ, Feng L, Klimont Z, Janssens-Maenhout G, Pitkanen T, Seibert JJ, Vu L, Andres RJ, Bolt RM, Bond TC, Dawidowski L, Kholod N, Kurokawa J, Li M, Liu L, Lu Z, Moura MCP, O’Rourke PR, Zhang Q, 2018. Historical (1750–2014) anthropogenic emissions of reactive gases and aerosols from the Community Emissions Data System (CEDS). Geosci Model Dev 11, 369–408. 10.5194/gmd-11-369-2018.

[R62] Hopke PK, 2016. Review of receptor modeling methods for source apportionment. J. Air Waste Manag. Assoc 66, 237–259. 10.1080/10962247.2016.1140693. 26756961

[R63] Hopke PK, Dai Q, Li L, Feng Y, 2020. Global review of recent source apportionments for airborne particulate matter. Sci. Total Environ 740, 140091 10.1016/j.scitotenv.2020.140091. 32559544 PMC7456793

[R64] Huang W, Zhu T, Pan X, Hu M, Lu S-E, Lin Y, Wang T, Zhang Y, Tang X, 2012. Air pollution and autonomic and vascular dysfunction in patients with cardiovascular disease: interactions of systemic inflammation, overweight, and gender. Am. J. Epidemiol 176, 117–126. 10.1093/aje/kwr511. 22763390 PMC3493195

[R65] Institute of Medicine (US), Sciences, R. on E.H., Research, Medicine, A., 2007. Global Environmental Health in the 21st Century: From Governmental Regulation to Corporate Social Responsibility: Workshop Summary., Global Environmental Health in the 21st Century: From Governmental Regulation to Corporate Social Responsibility: Workshop Summary. National Academies Press (US). 21595107

[R66] Janssen NAH, Hoek G, Simic-Lawson M, Fischer P, van Bree L, ten Brink H, Keuken M, Atkinson RW, Anderson HR, Brunekreef B, Cassee FR, 2011. Black carbon as an additional indicator of the adverse health effects of airborne particles compared with PM10 and PM2.5. Env. Health Perspect 119, 1691–1699. 10.1289/ehp.1003369. 21810552 PMC3261976

[R67] Jeuland MA, Bhojvaid V, Kar A, Lewis JJ, Patange O, Pattanayak SK, Ramanathan N, Rehman IH, Tan Soo JS, Ramanathan V, 2015. Preferences for improved cook stoves: Evidence from rural villages in north India. Energy Econ. 52, 287–298. 10.1016/j.eneco.2015.11.010.

[R68] Joeckel P, Kerkweg A, Pozzer A, Sander R, Tost H, Riede H, Baumgaertner A, Gromov S, Kern B, 2010. Development cycle 2 of the Modular Earth Submodel System (MESSy2). Geosci Model Dev 3, 717–752. 10.5194/gmd-3-717-2010.

[R69] Johnson MA, Steenland K, Piedrahita R, Clark ML, Pillarisetti A, Balakrishnan K, Peel JL, Naeher LP, Liao J, Wilson D, Sarnat J, Underhill LJ, Burrowes V, McCracken JP, Rosa G, Rosenthal J, Sambandam S, de LO, Kirby MA, Kearns K, Checkley W, Clasen T, null, null, 2020. Air Pollutant Exposure and Stove Use Assessment Methods for the Household Air Pollution Intervention Network (HAPIN) Trial. Environ. Health Perspect 128, 047009. 10.1289/EHP6422. 32347764 PMC7228125

[R70] Johnson TM, Guttikunda S, Wells GJ, Artaxo P, Bond TC, Russell AG, Watson JG, West J, 2011. Tools for Improving Air Quality Management : A Review of Top-down Source Apportionment Techniques and Their Application in Developing Countries. World Bank, Washington, DC.

[R71] Junker C, Wang J-L, Lee C-T, 2009. Evaluation of the effect of long-range transport of air pollutants on coastal atmospheric monitoring sites in and around Taiwan. Atmos. Environ 43, 3374–3384. 10.1016/j.atmosenv.2009.03.035.

[R72] Kajino M, Hagino H, Fujitani Y, Morikawa T, Fukui T, Onishi K, Okuda T, Igarashi Y, 2021. Simulation of the transition metal-based cumulative oxidative potential in East Asia and its emission sources in Japan. Sci. Rep 11, 6550. 10.1038/s41598-021-85894-z. 33753804 PMC7985388

[R73] Kar A, Pachauri S, Bailis R, Zerriffi H, 2019. Using sales data to assess cooking gas adoption and the impact of India’s Ujjwala programme in rural Karnataka. Nat. Energy 4, 806–814. 10.1038/s41560-019-0429-8.

[R74] Karagulian F, Van Dingenen R, Belis C, Janssens-Maenhout G, Crippa M, Guizzardi D, Dentener F, 2017. Attribution of anthropogenic PM_2.5_ to emission sources. European Commission. Joint Research Centre. Publications Office of the European Union, LU.

[R75] Karamchandani P, Long Y, Pirovano G, Balzarini A, Yarwood G, 2017. Source-sector contributions to European ozone and fine PM in 2010 using AQMEII modeling data. Atmospheric Chem. Phys 17, 5643–5664. 10.5194/acp-17-5643-2017.

[R76] KC S, Lutz W, 2014. The human core of the shared socioeconomic pathways: Population scenarios by age, sex and level of education for all countries to 2100. Glob. Environ. Change 42, 181–192. 10.1016/j.gloenvcha.2014.06.004. PMC5310112 28239237

[R77] Khan MN, Nurs B, C.Z., Mofizul Islam M, Islam MR, Rahman MM, 2017. Household air pollution from cooking and risk of adverse health and birth outcomes in Bangladesh: a nationwide population-based study. Environ. Health 16, 57. 10.1186/s12940-017-0272-y. 28610581 PMC5470285

[R78] Kim MJ, 2019. The effects of transboundary air pollution from China on ambient air quality in South Korea. Heliyon 5, e02953. 31890944 10.1016/j.heliyon.2019.e02953 PMC6926254

[R79] Kranenburg R, Segers AJ, Hendriks C, Schaap M, 2013. Source apportionment using LOTOS-EUROS: module description and evaluation. Geosci. Model Dev 6, 721–733. 10.5194/gmd-6-721-2013.

[R80] Kuenen JJP, Visschedijk AJH, Jozwicka M, Denier van der Gon H. a. C., 2014. TNO-MACC_II emission inventory; a multi-year (2003&ndash;2009) consistent high-resolution European emission inventory for air quality modelling. Atmospheric Chem. Phys 14, 10963–10976. 10.5194/acp-14-10963-2014.

[R81] Kukkonen J, López-Aparicio S, Segersson D, Geels C, Kangas L, Kauhaniemi M, Maragkidou A, Jensen A, Assmuth T, Karppinen A, Sofiev M, Hellén H, Riikonen K, Nikmo J, Kousa A, Niemi JV, Karvosenoja N, Santos GS, Sundvor I, Im U, Christensen JH, Nielsen O-K, Plejdrup MS, Nøjgaard JK, Omstedt G, Andersson C, Forsberg B, Brandt J, 2020. The influence of residential wood combustion on the concentrations of PM_2.5_ in four Nordic cities. Atmospheric Chem. Phys 20, 4333–4365. 10.5194/acp-20-4333-2020.

[R82] Kypridemos C, Puzzolo E, Aamaas B, Hyseni L, Shupler M, Aunan K, Pope D, 2020. Health and Climate Impacts of Scaling Adoption of Liquefied Petroleum Gas (LPG) for Clean Household Cooking in Cameroon: A Modeling Study. Environ. Health Perspect 128, 047001 10.1289/EHP4899. 32233878 PMC7228103

[R83] Laan T, Beaton C, Presta B, 2010. Strategies for Reforming Fossil-Fuel Subsidies: Practical lessons from Ghana, France and Senegal.

[R84] Lelieveld J, Barlas C, Giannadaki D, Pozzer A, 2013. Model calculated global, regional and megacity premature mortality due to air pollution. Atmos Chem Phys 13, 7023–7037. 10.5194/acp-13-7023-2013.

[R85] Lelieveld J, Evans JS, Fnais M, Giannadaki D, Pozzer A, 2015. The contribution of outdoor air pollution sources to premature mortality on a global scale. Nature 525, 367–371. 10.1038/nature15371. 26381985

[R86] Lelieveld J, Klingmueller K, Pozzer A, Poeschl U, Fnais M, Daiber A, Muenzel T, 2019. Cardiovascular disease burden from ambient air pollution in Europe reassessed using novel hazard ratio functions. Eur Heart J 40, 1590–1596. 10.1093/eurheartj/ehz135. 30860255 PMC6528157

[R87] Leung DYC, 2015. Outdoor-indoor air pollution in urban environment: challenges and opportunity. Front. Environ. Sci 2, 69. 10.3389/fenvs.2014.00069.

[R88] Li M, Zhang Q, Kurokawa J, Woo J-H, He K, Lu Z, Ohara T, Song Y, Streets DG, Carmichael GR, Cheng Y, Hong C, Huo H, Jiang X, Kang S, Liu F, Su H, Zheng B, 2017. MIX: a mosaic Asian anthropogenic emission inventory under the international collaboration framework of the MICS-Asia and HTAP. Atmospheric Chem. Phys 17, 935–963. 10.5194/acp-17-935-2017.

[R89] Liao H-T, Yau Y-C, Huang C-S, Chen N, Chow JC, Watson JG, Tsai S-W, Chou C-C-K, Wu C-F, 2017. Source apportionment of urban air pollutants using constrained receptor models with a priori profile information. Environ. Pollut 227, 323–333. 10.1016/j.envpol.2017.04.071. 28478370

[R90] Lin Y, Zou J, Yang W, Li C-Q, 2018. A Review of Recent Advances in Research on PM2.5 in China. Int. J. Environ. Res. Public. Health 15, E438. 10.3390/ijerph15030438. PMC5876983 29498704

[R91] Lippmann M, Chen L-C, Gordon T, Ito K, Thurston GD, 2013. National Particle Component Toxicity (NPACT) Initiative: integrated epidemiologic and toxicologic studies of the health effects of particulate matter components. Res Rep Health Eff Inst 177, 5–13. 24377209

[R92] Liu Q, Baumgartner J, Zhang Y, Liu Y, Sun Y, Zhang M, 2014. Oxidative Potential and Inflammatory Impacts of Source Apportioned Ambient Air Pollution in Beijing. Env. Sci Technol 48, 12920–12929. 10.1021/es5029876. 25279798

[R93] Liu J, Mauzerall DL, Chen Q, Zhang Q, Song Y, Peng W, Klimont Z, Qiu X, Zhang S, Hu M, Lin W, Smith KR, Zhu T, 2016. Air pollutant emissions from Chinese households: A major and underappreciated ambient pollution source. Proc. Natl. Acad. Sci 113, 7756–7761. 10.1073/pnas.1604537113. 27354524 PMC4948343

[R94] Lopez-Aparicio S, Grythe H, 2020. Evaluating the effectiveness of a stove exchange programme on PM2.5 emission reduction. Atmos. Environ 231, 117529 10.1016/j.atmosenv.2020.117529.

[R95] Lu X, Zhang S, Xing J, Wang Y, Chen W, Ding D, Wu Y, Wang S, Duan L, Hao J, 2020. Progress of Air Pollution Control in China and Its Challenges and Opportunities in the Ecological Civilization Era. Engineering 6, 1423–1431. 10.1016/j.eng.2020.03.014.

[R96] Lutz W, Striessnig E, Lutz W, Striessnig E, 2016. Demographic aspects of climate change mitigation and adaptation Demographic aspects of climate change mitigation and adaptation 4728. 10.1080/00324728.2014.969929.

[R97] Ma Q, Cai S, Wang S, Zhao B, Martin RV, Brauer M, Cohen A, Jiang J, Zhou W, Hao J, Frostad J, Forouzanfar MH, Burnett RT, 2017. Impacts of coal burning on ambient PM_2.5_ pollution in China. Atmospheric Chem. Phys 17, 4477–4491. 10.5194/acp-17-4477-2017.

[R98] Magalhaes S, Baumgartner J, Weichenthal S, 2018. Impacts of exposure to black carbon, elemental carbon, and ultrafine particles from indoor and outdoor sources on blood pressure in adults: A review of epidemiological evidence. Env. Res 161, 345–353. 10.1016/j.envres.2017.11.030. 29195183

[R99] Makoni M, 2020. Air pollution in Africa. Lancet Respir. Med 8, e60–e61. 10.1016/S2213-2600(20)30275-7. 32649923 PMC7340393

[R100] Mani S, Jain A, Tripathi S, Gould CF, 2020. The drivers of sustained use of liquified petroleum gas in India. Nat. Energy 5, 450–457. 10.1038/s41560-020-0596-7. 32719732 PMC7384753

[R101] MAPS Working Group, G., 2018. Burden of Disease Attributable to Major Air Pollution Sources in India.

[R102] Marais EA, Wiedinmyer C, 2016. Air Quality Impact of Diffuse and Inefficient Combustion Emissions in Africa (DICE-Africa). Environ. Sci. Technol 50, 10739–10745. 10.1021/acs.est.6b02602. 27611340

[R103] McDuffie EE, Smith SJ, O’Rourke P, Tibrewal K, Venkataraman C, Marais EA, Zheng B, Crippa M, Brauer M, Martin RV, 2020. A global anthropogenic emission inventory of atmospheric pollutants from sector- and fuel-specific sources (1970–2017): an application of the Community Emissions Data System (CEDS). Earth Syst. Sci. Data 12, 3413–3442. 10.5194/essd-12-3413-2020.

[R104] McDuffie EE, Martin RV, Spadaro JV, Burnett R, Smith SJ, O’Rourke P, Hammer MS, van Donkelaar A, Bindle L, Shah V, Jaeglé L, Luo G, Yu F, Adeniran JA, Lin J, Brauer M, 2021. Source sector and fuel contributions to ambient PM2.5 and attributable mortality across multiple spatial scales. Nat. Commun 12, 3594. 10.1038/s41467-021-23853-y. 34127654 PMC8203641

[R105] Mendez-Espinosa JF, Belalcazar LC, Morales Betancourt R, 2019. Regional air quality impact of northern South America biomass burning emissions. Atmos. Environ 203, 131–140. 10.1016/j.atmosenv.2019.01.042.

[R106] Meng W, Zhong Q, Chen Y, Shen H, Yun X, Smith KR, Li B, Liu J, Wang X, Ma J, Cheng H, Zeng EY, Guan D, Russell AG, Tao S, 2019. Energy and air pollution benefits of household fuel policies in northern China. Proc. Natl. Acad. Sci 116, 16773–16780. 10.1073/pnas.1904182116. 31383761 PMC6708357

[R107] Ministry of Petroleum and Natural Gas Government of India, 2016. Pradhan Mantri Ujjwala Yojana - Scheme Guidelines.

[R108] Ministry of Power Governmnet of India, 2014. Guidelines: Deendayal Upadhyaya Gram Jyoti Yojana (DDUGJY), Www.Rggvy.Gov.in.

[R109] Mircea M, Calori G, Pirovano G, Belis C, 2020. European guide on air pollution source apportionment for particulate matter with source oriented models and their combined use with receptor models. Publications Office of the European Union, LU.

[R110] Molina LT, Gallardo L, Andrade M, Baumgardner D, Borbor-Córdova M, Bórquez R, Casassa G, Cereceda-Balic F, Dawidowski L, Garreaud R, Huneeus N, Lambert F, McCarty JL, Phee JM, Mena-Carrasco M, Raga GB, Schmitt C, Schwarz JP, 2015. Pollution and its Impacts on the South American Cryosphere. Earths Future 3, 345–369. 10.1002/2015EF000311.

[R111] Multiconsult, 2020. Final Report : Study on the Potential of Increased Use of LPG for Cooking in Developing Countries.

[R112] Murray CJL, Aravkin AY, Zheng P, Abbafati C, Abbas KM, Abbasi-Kangevari M, Abd-Allah F, Abdelalim A, Abdollahi M, Abdollahpour I, Abegaz KH, Abolhassani H, Aboyans V, Abreu Z-J, Zhao JT, Zhao X-J-G, Zhao Y, Zhou M, Ziapour A, Zimsen SRM, Brauer M, Afshin A, Lim SS, 2020. Global burden of 87 risk factors in 204 countries and territories, 1990–2019: a systematic analysis for the Global Burden of Disease Study 2019. Lancet 396, 1223–1249. 10.1016/S0140-6736(20)30752-2. 33069327 PMC7566194

[R113] National Research Council, 2010. Global Sources of Local Pollution: An Assessment of Long-Range Transport of Key Air Pollutants to and from the United States. The National Academies Press, Washington, DC. 10.17226/12743.

[R114] Niranjan R, Thakur AK, 2017. The Toxicological Mechanisms of Environmental Soot (Black Carbon) and Carbon Black: Focus on Oxidative Stress and Inflammatory Pathways. Front Immunol 8. 10.3389/fimmu.2017.00763. PMC5492873 28713383

[R115] Okeahialam BN, 2016. The Cold Dusty Harmattan: A Season of Anguish for Cardiologists and Patients. Environ. Health Insights 10, 143–146. 10.4137/EHI.S38350. 27594787 PMC5004994

[R116] Orru H, Olstrup H, Kukkonen J, López-Aparicio S, Segersson D, Geels C, Tamm T, Riikonen K, Maragkidou A, Sigsgaard T, Brandt J, Grythe H, Forsberg B, 2022. Health impacts of PM2.5 originating from residential wood combustion in four nordic cities. BMC Public Health 22, 1286. 10.1186/s12889-022-13622-x. 35787793 PMC9252027

[R117] Park M, Joo HS, Lee K, Jang M, Kim SD, Kim I, Borlaza LJS, Lim H, Shin H, Chung KH, Choi Y-H, Park SG, Bae M-S, Lee J, Song H, Park K, 2018. Differential toxicities of fine particulate matters from various sources. Sci Rep 8, 1–11. 10.1038/s41598-018-35398-0. 30451941 PMC6242998

[R118] Pauchard A, Barbosa O, 2013. Regional Assessment of Latin America: Rapid Urban Development and Social Economic Inequity Threaten Biodiversity Hotspots, in: Elmqvist T, Fragkias M, Goodness J, Güneralp B, Marcotullio PJ, McDonald RI, Parnell S, Schewenius M, Sendstad M, Seto KC, Wilkinson C (Eds.), Urbanization, Biodiversity and Ecosystem Services: Challenges and Opportunities: A Global Assessment. Springer Netherlands, Dordrecht, pp. 589–608. 10.1007/978-94-007-7088-1_28.

[R119] Paudel D, Jeuland M, Lohani SP, 2021. Cooking-energy transition in Nepal: trend review. Clean Energy 5, 1–9. 10.1093/ce/zkaa022.

[R120] Pietrzak MB, Igliński B, Kujawski W, Iwański P, 2021. Energy Transition in Poland—Assessment of the Renewable Energy Sector. Energies 14, 2046. 10.3390/en14082046.

[R121] Pillarisetti A, Carter E, Rajkumar S, Young BN, Benka-Coker ML, Peel JL, Johnson M, Clark ML, 2019. Measuring personal exposure to fine particulate matter (PM2.5) among rural Honduran women: A field evaluation of the Ultrasonic Personal Aerosol Sampler (UPAS). Environ. Int 123, 50–53. 10.1016/j.envint.2018.11.014. 30496981 PMC6331229

[R122] Pillarisetti A, Ye W, Chowdhury S, 2022. Indoor Air Pollution and Health: Bridging Perspectives from Developing and Developed Countries. Annu. Rev. Environ. Resour 47, null. 10.1146/annurev-environ-012220-010602.

[R123] Pirovano G, Colombi C, Balzarini A, Riva GM, Gianelle V, Lonati G, 2015. PM2.5 source apportionment in Lombardy (Italy): Comparison of receptor and chemistry-transport modelling results. Atmos. Environ 106, 56–70. 10.1016/j.atmosenv.2015.01.073.

[R124] Pozzer A, Anenberg SC, Dey S, Haines A, Lelieveld J, Chowdhury S, 2023. Mortality Attributable to Ambient Air Pollution: A Review of Global Estimates. GeoHealth 7, e2022GH000711. 10.1029/2022GH000711. PMC9828848 36636746

[R125] Prank M, Sofiev M, Tsyro S, Hendriks C, Semeena V, Vazhappilly Francis X, Butler T, Denier van der Gon H, Friedrich R, Hendricks J, Kong X, Lawrence M, Righi M, Samaras Z, Sausen R, Kukkonen J, Sokhi R, 2016. Evaluation of the performance of four chemical transport models in predicting the aerosol chemical composition in Europe in 2005. Atmospheric Chem. Phys 16, 6041–6070. 10.5194/acp-16-6041-2016.

[R126] Pui DYH, Chen S-C, Zuo Z, 2014. PM2.5 in China: Measurements, sources, visibility and health effects, and mitigation. Particuology 13, 1–26. 10.1016/j.partic.2013.11.001.

[R127] Puxbaum H, Caseiro A, Sánchez-Ochoa A, Kasper-Giebl A, Claeys M, Gelencsér A, Legrand M, Preunkert S, Pio C, 2007. Levoglucosan levels at background sites in Europe for assessing the impact of biomass combustion on the European aerosol background. J. Geophys. Res. Atmospheres 112. 10.1029/2006JD008114.

[R128] Raunemaa T, Kulmala M, Saari H, Olin M, Kulmala MH, 1989. Indoor Air Aerosol Model: Transport Indoors and Deposition of Fine and Coarse Particles. Aerosol Sci. Technol 11, 11–25. 10.1080/02786828908959296.

[R129] Ravishankara AR, David LM, Pierce JR, Venkataraman C, 2020. Outdoor air pollution in India is not only an urban problem. Proc. Natl. Acad. Sci 117, 28640–28644. 10.1073/pnas.2007236117. 33139542 PMC7682420

[R130] Reddington CL, Conibear L, Knote C, Silver BJ, Li YJ, Chan CK, Arnold SR, Spracklen DV, 2019. Exploring the impacts of anthropogenic emission sectors on PM2.5 and human health in South and East Asia. Atmospheric Chem. Phys 19, 11887–11910. 10.5194/acp-19-11887-2019.

[R131] Rees N, Wickham A, Choi Y, 2019. Silent Suffocation in Africa Air Pollution is a Growing Menace, Affecting the Poorest Children the Most. UNICEF, NY.

[R132] Riahi K, Vuuren DPV, Kriegler E, Edmonds J, Neill BCO, Fujimori S, Bauer N, Calvin K, Dellink R, Fricko O, Lutz W, Popp A, Crespo J, Kc S, Leimbach M, Jiang L, Kram T, Rao S, Emmerling J, Ebi K, Hasegawa T, Havlik P, Humpenöder F, Aleluia L, Silva D, Smith S, Stehfest E, Bosetti V, Eom J, Gernaat D, Masui T, Rogelj J, Stre J, Drouet L, Krey V, Luderer G, Harmsen M, Takahashi K, Baumstark L, Doelman JC, Kainuma M, Klimont Z, Marangoni G, Lotze-campen H, Obersteiner M, Tabeau A, Tavoni M, 2017. The Shared Socioeconomic Pathways and their energy , land use , and greenhouse gas emissions implications: An overview. 42, 153–168. 10.1016/j.gloenvcha.2016.05.009.

[R133] Romieu I, Weitzenfeld H, Finkelman J, 1990. Urban air pollution in Latin America and the Caribbean: health perspectives. World Health Stat. Q. Rapp. Trimest. Stat. Sanit. Mond 43, 153–167. 2238696

[R134] Ross K, Chmiel JF, Ferkol T, 2012. The impact of the Clean Air Act. J. Pediatr 161, 781–786. 10.1016/j.jpeds.2012.06.064. 22920509 PMC4133758

[R135] Sadavarte P, Venkataraman C, 2014. Trends in multi-pollutant emissions from a technology-linked inventory for India: I. Industry and transport sectors. Atmos. Environ 99, 353–364. 10.1016/j.atmosenv.2014.09.081.

[R136] Saikawa E, Kim H, Zhong M, Avramov A, Zhao Y, Janssens-Maenhout G, Kurokawa J, Klimont Z, Wagner F, Naik V, Horowitz LW, Zhang Q, 2017. Comparison of emissions inventories of anthropogenic air pollutants and greenhouse gases in China. Atmospheric Chem. Phys 17, 6393–6421. 10.5194/acp-17-6393-2017.

[R137] Savolahti M, Lehtomäki H, Karvosenoja N, Paunu V-V, Korhonen A, Kukkonen J, Kupiainen K, Kangas L, Karppinen A, Hänninen O, 2019. Residential Wood Combustion in Finland: PM2.5 Emissions and Health Impacts with and without Abatement Measures. Int. J. Environ. Res. Public. Health 16, 2920. 10.3390/ijerph16162920. 31416284 PMC6719946

[R138] Schwela D, 2012. Review of Urban Air Quality in Sub-Saharan Africa Region : Air Quality Profile of SSA Countries. World Bank Group.

[R139] Shaddick G, Thomas ML, Green A, Brauer M, van Donkelaar A, Burnett R, Chang HH, Cohen A, Dingenen RV, Dora C, Gumy S, Liu Y, Martin R, Waller LA, West J, Zidek JV, Prüss-Ustün A, 2018. Data integration model for air quality: a hierarchical approach to the global estimation of exposures to ambient air pollution. J. R. Stat. Soc. Ser. C Appl. Stat 67, 231–253. 10.1111/rssc.12227.

[R140] Shaddick G, Thomas ML, Mudu P, Ruggeri G, Gumy S, 2020. Half the world’s population are exposed to increasing air pollution. Npj Clim. Atmospheric Sci 3, 1–5. 10.1038/s41612-020-0124-2.

[R141] Sharma S, Bawase M, Ghosh P, Saraf M, Goel A, Suresh R, dutta A, Jhajra ajeet, Kundu S, sharma ved, malik jai, rehman hafeez, Khandaskar H, Mulla S, Sharma R, Bansal A, Mane S, Reve S, Markad A, Shaikh AR, 2018. Source Apportionment of PM2.5 & PM10 in Delhi NCR. 10.13140/RG.2.2.19621.55520.

[R142] Shen H, Luo Z, Xiong R, Liu X, Zhang L, Li Y, Du W, Chen Y, Cheng H, Shen G, Tao S, 2021. A critical review of pollutant emission factors from fuel combustion in home stoves. Environ. Int 157, 106841 10.1016/j.envint.2021.106841. 34438232

[R143] Shen G, Ru M, Du W, Zhu X, Zhong Q, Chen Y, Shen H, Yun X, Meng W, Liu J, Cheng H, Hu J, Guan D, Tao S, 2019. Impacts of air pollutants from rural Chinese households under the rapid residential energy transition. Nat. Commun 10, 3405. 10.1038/s41467-019-11453-w. 31363099 PMC6667435

[R144] Silva RA, Adelman Z, Fry MM, West JJ, 2016. The Impact of Individual Anthropogenic Emissions Sectors on the Global Burden of Human Mortality due to Ambient Air Pollution. Env. Health Perspect 124, 1776–1784. 10.1289/EHP177. 27177206 PMC5089880

[R145] Silver B, Reddington CL, Arnold SR, Spracklen DV, 2018. Substantial changes in air pollution across China during 2015–2017. Environ. Res. Lett 13, 114012 10.1088/1748-9326/aae718.

[R146] Smith KR, 1993. Fuel combustion, air pollution exposure, and health: the situation in developing countries. Annu. Rev. Energy Environ 18, 529–566.

[R147] Smith KR, Pillarisetti A, 2017. Household air pollution from solid cookfuels and Its Effects on Health 133–152. 30212117

[R148] Smith KR, Bruce N, Balakrishnan K, Adair-Rohani H, Balmes J, Chafe Z, Dherani M, Hosgood HD, Mehta S, Pope D, Rehfuess E, 2014. Millions Dead: How Do We Know and What Does It Mean? Methods Used in the Comparative Risk Assessment of Household Air Pollution. Annu Rev Public Health 35, 185–206. 10.1146/annurev-publhealth-032013-182356. 24641558

[R149] Smith KR, 2018. Pradhan Mantri Ujjwala Yojana: Transformation of Subsidy to Social Investment in India, in: Ch 29 in Debroy B, Gangul A, Desai K (Eds) Making of New India: Transformation Under Modi Government, Dr. Syama Prasad Mookerjee Research Foundation and Wisdom Tree, New Delhi. pp. 401–410.

[R150] Snider G, Weagle CL, Murdymootoo KK, Ring A, Ritchie Y, Stone E, Walsh A, Akoshile C, Anh NX, Balasubramanian R, Brook J, Qonitan FD, Dong J, Griffith D, He K, Holben BN, Kahn R, Lagrosas N, Lestari P, Ma Z, Misra A, Norford LK, Quel EJ, Salam A, Schichtel B, Segev L, Tripathi S, Wang C, Yu C, Zhang Q, Zhang Y, Brauer M, Cohen A, Gibson MD, Liu Y, Martins JV, Rudich Y, Martin RV, 2016. Variation in global chemical composition of PM_2.5_: emerging results from SPARTAN. Atmospheric Chem. Phys 16, 9629–9653. 10.5194/acp-16-9629-2016.

[R151] Solazzo E, Crippa M, Guizzardi D, Muntean M, Choulga M, Janssens-Maenhout G, 2021. Uncertainties in the Emissions Database for Global Atmospheric Research (EDGAR) emission inventory of greenhouse gases. Atmospheric Chem. Phys 21, 5655–5683. 10.5194/acp-21-5655-2021.

[R152] Tagle M, Pillarisetti A, Hernandez MT, Troncoso K, Soares A, Torres R, Galeano A, Oyola P, Balmes J, Smith KR, 2019. Monitoring and modeling of household air quality related to use of different Cookfuels in Paraguay. Indoor Air 29, 252–262. 10.1111/ina.12513. 30339298 PMC6849814

[R153] Tao S, Ru MY, Du W, Zhu X, Zhong QR, Li BG, Shen GF, Pan XL, Meng WJ, Chen YL, Shen HZ, Lin N, Su S, Zhuo SJ, Huang TB, Xu Y, Yun X, Liu JF, Wang XL, Liu WX, Cheng HF, Zhu DQ, 2018. Quantifying the rural residential energy transition in China from 1992 to 2012 through a representative national survey. Nat. Energy 3, 567–573. 10.1038/s41560-018-0158-4.

[R154] Thunis P, Degraeuwe B, Pisoni E, Trombetti M, Peduzzi E, Belis CA, Wilson J, Clappier A, Vignati E, 2018. PM2.5 source allocation in European cities: A SHERPA modelling study. Atmos. Environ 187, 93–106. 10.1016/j.atmosenv.2018.05.062.

[R155] Thunis P, Clappier A, Tarrason L, Cuvelier C, Monteiro A, Pisoni E, Wesseling J, Belis CA, Pirovano G, Janssen S, Guerreiro C, Peduzzi E, 2019. Source apportionment to support air quality planning: Strengths and weaknesses of existing approaches. Environ. Int 130, 104825 10.1016/j.envint.2019.05.019. 31226558 PMC6686078

[R156] Timmermans R, Kranenburg R, Manders A, Hendriks C, Segers A, Dammers E, Zhang Q, Wang L, Liu Z, Zeng L, Denier van der Gon H, Schaap M, 2017. Source apportionment of PM2.5 across China using LOTOS-EUROS. Atmos. Environ 164, 370–386. 10.1016/j.atmosenv.2017.06.003.

[R157] Tong D, Cheng J, Liu Y, Yu S, Yan L, Hong C, Qin Y, Zhao H, Zheng Y, Geng G, Li M, Liu F, Zhang Y, Zheng B, Clarke L, Zhang Q, 2020. Dynamic projection of anthropogenic emissions in China: methodology and 2015–2050 emission pathways under a range of socio-economic, climate policy, and pollution control scenarios. Atmospheric Chem. Phys 20, 5729–5757. 10.5194/acp-20-5729-2020.

[R158] Upadhyay A, Dey S, Chowdhury S, Goyal P, 2018. Expected health benefits from mitigation of emissions from major anthropogenic PM2.5 sources in India: Statistics at state level. Environ. Pollut 242, 1817–1826. 10.1016/j.envpol.2018.07.085. 30078683

[R159] US EPA, 2013. Summary of the Clean Air Act [WWW Document]. URL https://www.epa.gov/laws-regulations/summary-clean-air-act (accessed 8.30.21).

[R160] US EPA, 2018. Proposed Amendments to the New Source Performance Standards for Residential Wood Heaters [WWW Document]. URL https://www.epa.gov/residential-wood-heaters/proposed-amendments-new-source-performance-standards-residential-wood (accessed 8.30.21).

[R161] US EPA, 2020. Final 2020 New Source Performance Standards for Residential Wood Heaters [WWW Document]. URL https://www.epa.gov/residential-wood-heaters/final-2020-new-source-performance-standards-residential-wood-heaters (accessed 8.30.21).

[R162] Venkataraman C, Brauer M, Tibrewal K, Sadavarte P, Ma Q, Cohen A, Chaliyakunnel S, Frostad J, Klimont Z, Martin RV, Millet DB, Philip S, Walker K, Wang S, 2018. Source influence on emission pathways and ambient PM2.5pollution over India (2015–2050). Atmospheric Chem. Phys 18, 8017–8039. 10.5194/acp-18-8017-2018. PMC7935015 33679902

[R163] Venkataraman C, Bhushan M, Dey S, Ganguly D, Gupta T, Habib G, Kesarkar A, Phuleria H, Raman RS, 2020. Indian Network Project on Carbonaceous Aerosol Emissions, Source Apportionment and Climate Impacts (COALESCE). Bull Am Meteorol Soc 101, E1052–E1068. 10.1175/BAMS-D-19-0030.1.

[R164] Weagle CL, Snider G, Li C, van Donkelaar A, Philip S, Bissonnette P, Burke J, Jackson J, Latimer R, Stone E, Abboud I, Akoshile C, Anh NX, Brook JR, Cohen A, Dong J, Gibson MD, Griffith D, He KB, Holben BN, Kahn R, Keller CA, Kim JS, Lagrosas N, Lestari P, Khian YL, Liu Y, Marais EA, Martins JV, Misra A, Muliane U, Pratiwi R, Quel EJ, Salam A, Segev L, Tripathi SN, Wang C, Zhang Q, Brauer M, Rudich Y, Martin RV, 2018. Global Sources of Fine Particulate Matter: Interpretation of PM2.5 Chemical Composition Observed by SPARTAN using a Global Chemical Transport Model. Environ. Sci. Technol 52, 11670–11681. 10.1021/acs.est.8b01658. 30215246

[R165] WHO Regional Office for Europe, 2013. Review of evidence on health aspects of air pollution – REVIHAAP First results. 27195369

[R166] Xie P, Liao H, 2022. The Impacts of Changes in Anthropogenic Emissions Over China on PM2.5 Concentrations in South Korea and Japan During 2013–2017. Front. Environ. Sci 10.

[R167] Yim SHL, Gu Y, Shapiro MA, Stephens B, 2019. Air quality and acid deposition impacts of local emissions and transboundary air pollution in Japan and South Korea. Atmospheric Chem. Phys 19, 13309–13323. 10.5194/acp-19-13309-2019.

[R168] Yun X, Shen G, Shen H, Meng W, Chen Y, Xu H, Ren Y, Zhong Q, Du W, Ma J, Cheng H, Wang X, Liu J, Wang X, Li B, Hu J, Wan Y, Tao S, 2020. Residential solid fuel emissions contribute significantly to air pollution and associated health impacts in China. Sci. Adv 6, eaba7621. 10.1126/sciadv.aba7621. 33115732 PMC7608780

[R169] Zhang Q, Zheng Y, Tong D, Shao M, Wang S, Zhang Y, Xu X, Wang J, He H, Liu W, Ding Y, Lei Y, Li J, Wang Z, Zhang X, Wang Y, Cheng J, Liu Y, Shi Q, Yan L, Geng G, Hong C, Li M, Liu F, Zheng B, Cao J, Ding A, Gao J, Fu Q, Huo J, Liu B, Liu Z, Yang F, He K, Hao J, 2019. Drivers of improved PM2.5 air quality in China from 2013 to 2017. Proc. Natl. Acad. Sci 116, 24463–24469. 10.1073/pnas.1907956116. 31740599 PMC6900509

[R170] Zhao B, Zheng H, Wang S, Smith KR, Lu X, Aunan K, Gu Y, Wang Y, Ding D, Xing J, Fu X, Yang X, Liou K-N, Hao J, 2018. Change in household fuels dominates the decrease in PM2.5 exposure and premature mortality in China in 2005–2015. Proc. Natl. Acad. Sci 115, 12401–12406. 10.1073/pnas.1812955115. 30455309 PMC6298076

[R171] Zheng B, Cheng J, Geng G, Wang X, Li M, Shi Q, Qi J, Lei Y, Zhang Q, He K, 2021. Mapping anthropogenic emissions in China at 1 km spatial resolution and its application in air quality modeling. Sci. Bull 66, 612–620. 10.1016/j.scib.2020.12.008. 36654431

