# Supplementary material for: A global review of the state of the evidence of household air pollution’s contribution to ambient fine particulate matter and their related health impacts
Source: Environ Int. Author manuscript; Available in PMC 2024 Mar 1. (PMC10378453; doi:10.1016/j.envint.2023.107835)
Supplement: SI [file NIHMS1911072-supplement-SI.docx]

**Supplementary Information**

A global review of the state of the evidence of household air pollution's contribution to ambient fine particulate matter and their related health impacts

Sourangsu Chowdhury^1*^, Ajay Pillarisetti^2*^, Alicia Oberholzer^3^, James Jetter^4^, John Mitchell^4^, Eva Cappuccilli^4^, Borgar Aamaas^1^, Kristin Aunan^1^, Andrea Pozzer^5^, Donee Alexander^3^

^1^ CICERO Center for International Climate Research, Oslo, Norway

^2^ University of California, Berkeley, Berkeley, USA

^3^ Clean Cooking Alliance, Washington D.C., USA

^4^ United States Environmental Protection Agency, Washington D.C. USA

^5^ Max Planck Institute for Chemistry, Mainz, Germany

*Draft for Environment International https://www.sciencedirect.com/journal/environment-international*

*corresponding authors- [sourangsu.chowdhury@cicero.oslo.no](mailto:sourangsu.chowdhury@cicero.oslo.no), [ajayp@berkeley.edu](mailto:ajayp@berkeley.edu)

**Supplementary Information Text**

**SI Text 1**

**1.1. Household air pollution (HAP)**

Burning biomass in simple stoves produces a vast range of products of incomplete combustion (PICs) . These include particulate matter (both fine and coarse), nitrogen oxides (NOx), CO, sulfur oxides (SOx), polycyclic aromatic hydrocarbons (PAHs) and other hydrocarbons, and other organic substances. Combustion of charcoal emits comparably lower levels of particulate matter and toxic organic gases, but higher levels of CO per unit fuel than wood and other types of biomass fuels (eg. crop residues and dung cakes). Coal combustion, in addition to the above-mentioned PICs, releases large amounts of sulfur oxides. In an indoor kitchen with little ventilation, cooking with solid fuels may result in extremely elevated PM2.5 concentrations [(Balakrishnan et al., 2013; Li et al., 2016; Naeher et al., 2000, 2007; Siddiqui et al., 2009)](https://www.zotero.org/google-docs/?p5iLlW), often reaching thousands of micrograms per cubic meter and causing much eye and throat irritation in addition to various respiratory symptoms [(Balmes, 2019; James et al., 2020; Naeher et al., 2007; Smith and Pillarisetti, 2017)](https://www.zotero.org/google-docs/?2m2k7J). Exposure to HAP can be estimated by monitoring indoor pollutant concentrations combined with time-activity information of the residents [(Mestl et al., 2007; Saini et al., 2020; Shupler et al., 2020)](https://www.zotero.org/google-docs/?lgslZz). However, measuring personal exposure directly using portable monitoring devices worn by participants might provide more accurate information [(Gaskins and Hart, 2020; Hu et al., 2019; Liao et al., 2020; Pillarisetti et al., 2019; Tagle et al., 2019)](https://www.zotero.org/google-docs/?5ndYU7).

Numerous studies have linked HAP to multiple diseases, including acute lower respiratory infection (ALRI), diseases from preterm birth and low birth weight among children younger than 5 years, chronic obstructive pulmonary diseases, lung cancer, cardiovascular diseases, and cataract among adults [(Balmes, 2019; Murray et al., 2020; Naeher et al., 2007; Smith et al., 2014; Smith and Pillarisetti, 2017)](https://www.zotero.org/google-docs/?LmgP09). The recent Global Burden of Disease study [(Murray et al., 2020)](https://www.zotero.org/google-docs/?j5Ukh2) associated 91 (67-118) million DALYs and 2.3 (1.6-3.1) million deaths with HAPhousehold air pollution exposure globally, of which ~66% occur in South Asia and sub-Saharan Africa, while ~1% occurs in mainland Europe. In high-income North America, this fraction is negligible.

**1.2. Ambient air pollution (AAP)**

Ambient air pollution (AAP) is air pollution in outdoor environments and is a mixture of noxious gasses like tropospheric ozone, NOx, SO2, and fine particulates like PM2.5. They are emitted from multiple sources, including transportation, industry, power generation, agricultural practices, waste burning, and emissions from household solid fuel use. WHO redefined air quality guidelines in 2021 [(WHO 2021)](https://www.zotero.org/google-docs/?W48yEU); the latest update to the guidelines recommended upper threshold values for exposures to specific pollutants. Many countries have defined higher threshold levels or do not manage air quality at all. Over the last decade, studies have found health impacts associated with exposures below current guideline values [(Achakulwisut et al., 2019; Chen and Hoek, 2020; Chowdhury et al., 2021, 2020; Yu et al., 2020)](https://www.zotero.org/google-docs/?EamUyQ).

PM2.5 concentrations, which are an important marker of AAP, consist of a mixture of black carbon, primary and secondary organic aerosols, secondary inorganic aerosols, mineral dust, and sea salt [(Snider et al., 2016)](https://www.zotero.org/google-docs/?x8VpFM). Direct releases of PM into the air are called primary emissions; they may be of natural or anthropogenic (like black carbon from transportation, industries or HAP) origin. Secondary particles are chemically formed within the atmosphere from emissions of precursor gases such as SO_2_ and NO_2_. Organic gases emitted from solvent use, industries, transportation, and HAP also oxidize to form secondary organic aerosols [(Platt et al., 2012; Tsimpidi et al., 2018)](https://www.zotero.org/google-docs/?S4qdxK).

Knowledge of the global distribution of ambient PM2.5 has improved over the last decade, with many countries expanding their regulatory networks of ground-based monitors [(Martin et al., 2019; Snider et al., 2016)](https://www.zotero.org/google-docs/?gNliad). Open data initiatives, such as OpenAQ (openaq.org), have increased data accessibility and contributed to improving awareness of local air quality. Despite recent growth in PM2.5 monitoring, most regions are insufficiently monitored, limiting air quality management [(Martin et al., 2019)](https://www.zotero.org/google-docs/?en7ZNu). Given the paucity of ground-based PM2.5 monitoring, alternative monitoring resources like data-informed global atmospheric models and satellite retrievals are widely used for exposure assessments [(Anenberg et al., 2010a; Apte et al., 2015; Boys et al., 2014; Chowdhury et al., 2019a; Hammer et al., 2020; Lelieveld et al., 2015; Van Donkelaar et al., 2016)](https://www.zotero.org/google-docs/?gpoJP9). More sophisticated methods combine information from satellite remote sensing data, chemical transport models ([Brasseur, 1997; Brasseur and Jacob, 2017)](https://www.zotero.org/google-docs/?kjvnTH), and ground-based monitors to provide PM2.5 levels with high confidence [(Hammer et al., 2020; Shaddick et al., 2018; Van Donkelaar et al., 2016)](https://www.zotero.org/google-docs/?pXewkP).

The physical response to PM2.5 exposure includes deposition in the lungs; the resulting inflammation particularly impacts people with pre-existing illness [(Dockery and Pope III, 1994; Münzel et al., 2018; Pope and Dockery, 2006; Wang et al., 2020; Xing et al., 2016)](https://www.zotero.org/google-docs/?mX3dwu). Studies also find significant relationships between short term changes in PM2.5 exposure with disease aberration and premature death [(Orellano et al., 2020; Tasmin et al., 2019; Wyatt et al., 2020; X. Zheng et al., 2021)](https://www.zotero.org/google-docs/?q3pWHK). Inflammation of the lower respiratory tract releases messenger substances that carry inflammatory responses throughout the body, reducing the elasticity of blood vessels and contributing to blood clotting, atherosclerosis, and diseases that lead to heart attacks and strokes [(Münzel et al., 2018)](https://www.zotero.org/google-docs/?1CMo19). Inflammatory responses have also been linked to type 2 diabetes and neurological disorders [(Hahad et al., 2021; Lao et al., 2019; Pearson et al., 2010; Shi et al., 2020)](https://www.zotero.org/google-docs/?d4rfZA). In addition, ambient air pollution is also associated with adverse birth outcomes and multiple neonatal diseases [(Murray et al., 2020)](https://www.zotero.org/google-docs/?KuXJsY).

**SI Text 2**

**2.1. Emission Inventories**. Formulating an emission inventory [(Crippa et al., 2018; McDuffie et al., 2020; Trombetti et al., 2018)](https://www.zotero.org/google-docs/?0uO0Sk) with reliable information on air pollution sources is a critical part of the bottom-up approach. Building an emission inventory demands extensive efforts and often takes years. Inventorying an emission change - as from a new policy – likely cannot occur concomitant with the policy. The selection of an emissions inventory also plays a major role in determining how accurately models simulate ambient pollutant concentrations. Use of inventories which fail to incorporate fine details (e.g. type and amount of solid fuel used in each household by region and end use, for instance wood use in water heating stoves among rural households in a state of India) results in uncertainty. There is also significant heterogeneity among existing emission inventories on how different air pollution sources are categorized and grouped. Potential uncertainties in emission factors, unexpected sources (such as trash burning), inclusion of evolving emission standards, and knowledge of task-specific fuel type, among others, makes formulating an accurate emission inventory a daunting task.

For a specific region of interest, it is expected that emission inventories designed locally [(Sadavarte and Venkataraman, 2014; Tong et al., 2020; Venkataraman et al., 2020; B. Zheng et al., 2021)](https://www.zotero.org/google-docs/?Kr6eFY) incorporate finer detail than global emission inventories. For example, recent Indian inventories [(Sadavarte and Venkataraman, 2014; Venkataraman et al., 2020)](https://www.zotero.org/google-docs/?FS9UCx) take into account space and water heating behaviors observed in parts of India where it has been assumed non-existent in other inventories (e.g.,. Emissions Database for Global Atmospheric Research or EDGAR). A recent study [(Saikawa et al., 2017)](https://www.zotero.org/google-docs/?JuKDiD) found that large disagreements exist among the five inventories used for China at disaggregated levels. These disagreements lead to large differences of 67 µg m−3 for PM10, and similarly large differences for O3 and CO. The study further found that for the residential sector, estimates from local emission inventories were always higher than those from the global inventory.

Different emission inventories also have distinct sector classification. For example, EDGAR and the Community Emissions Data System (CEDS) classify household solid fuel use under the umbrella of the ‘residential and commercial sector,’ which includes emissions from households and the commercial sector. These emission databases assume that such activities consume similar fuels for similar purposes and use an Intergovernmental Panel on Climate Change code (https://www.ipcc-nggip.iges.or.jp/public/gl/invs1.html) for grouping the sectors. However, these inclusions may add ~5% more emissions on top of those actually from households, more so in regions where diesel generator use is prevalent. Ideally, disaggregated emission data (i.e. household cooking and heating, diesel generators etc.) should be made available, though such is not the case currently. In contrast, emissions inventories which include only household activities, like cooking, water heating, space heating, and lighting are expected to provide a more accurate picture of the contribution of HAP to AAP [(Chowdhury et al., 2019c)](https://www.zotero.org/google-docs/?G3SiBb). A detailed inter-emission inventory comparison may help better understand how inclusion of these additional sources (besides the conventional household sources) impact estimates of the contribution of household emissions to ambient air pollution.

**2.2. Model types and configurations.** Atmospheric chemistry models incorporate different physical and chemical processes necessary to predict time-varying PM2.5 concentrations. Careful choices should be made about (a) spatial and temporal resolution; (b) initial and boundary conditions for chemistry and aerosols if required (as in regional air pollution models); (c) photo-chemical reactions included in the chemical mechanism, both for the gas and the aerosols phase; (d) deposition processes; and (e) reproduction of observed meteorology.

Coarsely resolved global models (with grid boxes representing large areas), underestimate variability and concentrations of pollutants in the most polluted regions of the globe, which are also regions with prevalent household solid fuel use [(Chowdhury et al., 2022; Lelieveld et al., 2015; McDuffie et al., 2021; Silva et al., 2016)](https://www.zotero.org/google-docs/?4oFZbC). Regional models, meanwhile, are often better-suited to local conditions not captured by global models and thus provide more accurate estimates of exposures and sources [(Feser et al., 2011)](https://www.zotero.org/google-docs/?lLJNFX). A regional model inter-comparison study for Europe [(Prank et al., 2016)](https://www.zotero.org/google-docs/?cwGjyE) found notable differences between predictions of seasonal variations of particulate matter attributable to different emission inventories and aerosol processes. Conversely, global models capture long-range aerosol transport across regions better than regional models by virtue of their broad geographic extent.

Meteorology plays a fundamental role in modulating the life-cycle of simulated aerosols (e.g., wet and dry deposition, transport). Hence particular care must be given to accurately reproduce meteorology, as different inputs change AAP estimates. For example, using meteorological parameters from two different years may provide two distinct outputs. Similarly, using meteorological inputs for the same year but from different data sources may provide different outputs [(Arciszewska and McClatchey, 2001; Lewellen and Sykes, 1989; Pérez et al., 2020)](https://www.zotero.org/google-docs/?v7JkLE).

Models capable of performing simulations at finer resolutions in higher detail are computationally intense. Global models generally used to simulate at coarse resolutions are typically incapable of identifying local pollution hotspots [(Butt et al., 2016; Silva et al., 2016)](https://www.zotero.org/google-docs/?wD4WwC). However, some studies [(Chowdhury et al., 2022; Lelieveld et al., 2013)](https://www.zotero.org/google-docs/?cI2AC7) indicate that the choice of model resolution may not necessarily be the leading cause of uncertainty, especially in Europe and North America. Large model intercomparison initiatives are needed to detect the sensitivity of model inputs as in [Prank et al., (2016)](https://www.zotero.org/google-docs/?5ZkneD). An alternative approach is generation of cumulative ensemble models leveraging the variability in estimated model outputs (Meehl et al., 2000).

**2.3. Health impact assessment.** Estimating excess deaths attributable to PM2.5 exposure requires four major inputs: (1) the distribution of population-weighted exposures; (2) specification of a level of exposure below which no increased risk of mortality is assumed to exist (the counterfactual concentration); (3) estimates of the relative risk, obtained through exposure-response functions (ERFs); (4) estimates of baseline mortality rates and detailed demographic information [(Burnett and Cohen, 2020; Chowdhury and Dey, 2016; Murray et al., 2020)](https://www.zotero.org/google-docs/?klzmev). Variations in each of these parameters impacts overall burden estimates.

To date, there are four varieties of exposure response models proposed for assessing the population mortality burden due to long term PM2.5 exposure: log linear (LL), integrated exposure-response (IER), Global Exposure Mortality Model (GEMM), and the meta-regression—Bayesian, regularized, trimmed (MR-BRT) models. Prior to 2010, LL models [(Anenberg et al., 2010b)](https://www.zotero.org/google-docs/?XSucHp) were used for health impact assessment until the advent of the IER [(Burnett et al., 2014)](https://www.zotero.org/google-docs/?h59i5T). The IER has evolved by incorporating novel data from cohort studies, by changing model fitting methods, and by inclusion of additional health points deemed of sufficient evidence to be causally associated with air pollution exposure [(Cohen et al., 2017; Stanaway et al., 2018)](https://www.zotero.org/google-docs/?NZ9e0l). The IERs use information from cohort studies spanning a broad range of exposures, from AAP, to secondhand smoke, to HAP and active tobacco smoking [(Burnett et al., 2014)](https://www.zotero.org/google-docs/?6u2ixO). The IERs in their original formulation allowed estimation of excess deaths for chronic obstructive pulmonary disease, ischemic heart disease, stroke, lung cancer, and acute lower respiratory infection.

Since the IERs, [(Burnett et al., 2014)](https://www.zotero.org/google-docs/?1kNYCu) combined information from 41 cohort studies on ambient air pollution has been used to build a shape-constrained exposure response function for estimating premature mortality from all non-communicable diseases and lower respiratory infections(the GEMM). The latest global burden of disease study [(Murray et al., 2020)](https://www.zotero.org/google-docs/?iqK2Vj) introduced MR-BRT splines, which, like the IER, integrates across source categories, but excludes active tobacco smoking. The MR-BRT functions include multiple recent studies in high exposure settings [(Hystad et al., 2019; Murray et al., 2020; Yusuf et al., 2020)](https://www.zotero.org/google-docs/?5ey06r) and include risk functions for excess mortality from low-birth weight and short gestation among neonates (population with age 0-27 days).

It should be noted that use of a different ERF can result in a substantial change in burden attributed to ambient PM2.5 caused by HAP emissions. For example, Lelieveld and colleagues [(Lelieveld et al., 2015)](https://www.zotero.org/google-docs/?5BSM7Y) used IERs to find that globally ~1 million premature deaths from AAP exposure may be averted if HAP is completely mitigated; however, using the same model with GEMM ERFs found that ~3 million premature deaths may be averted by mitigating solid fuel use [(Lelieveld et al., 2019)](https://www.zotero.org/google-docs/?Ne0Of7). Given the variability in burden estimates induced by changes in ERFs, an ensemble approach that incorporates suitable features of existing ERFs, while addressing limitations in each, as discussed in [Burnett et al., (2022)](https://www.zotero.org/google-docs/?Cg8M0m) is recommended.

**Supplementary Figures**

**
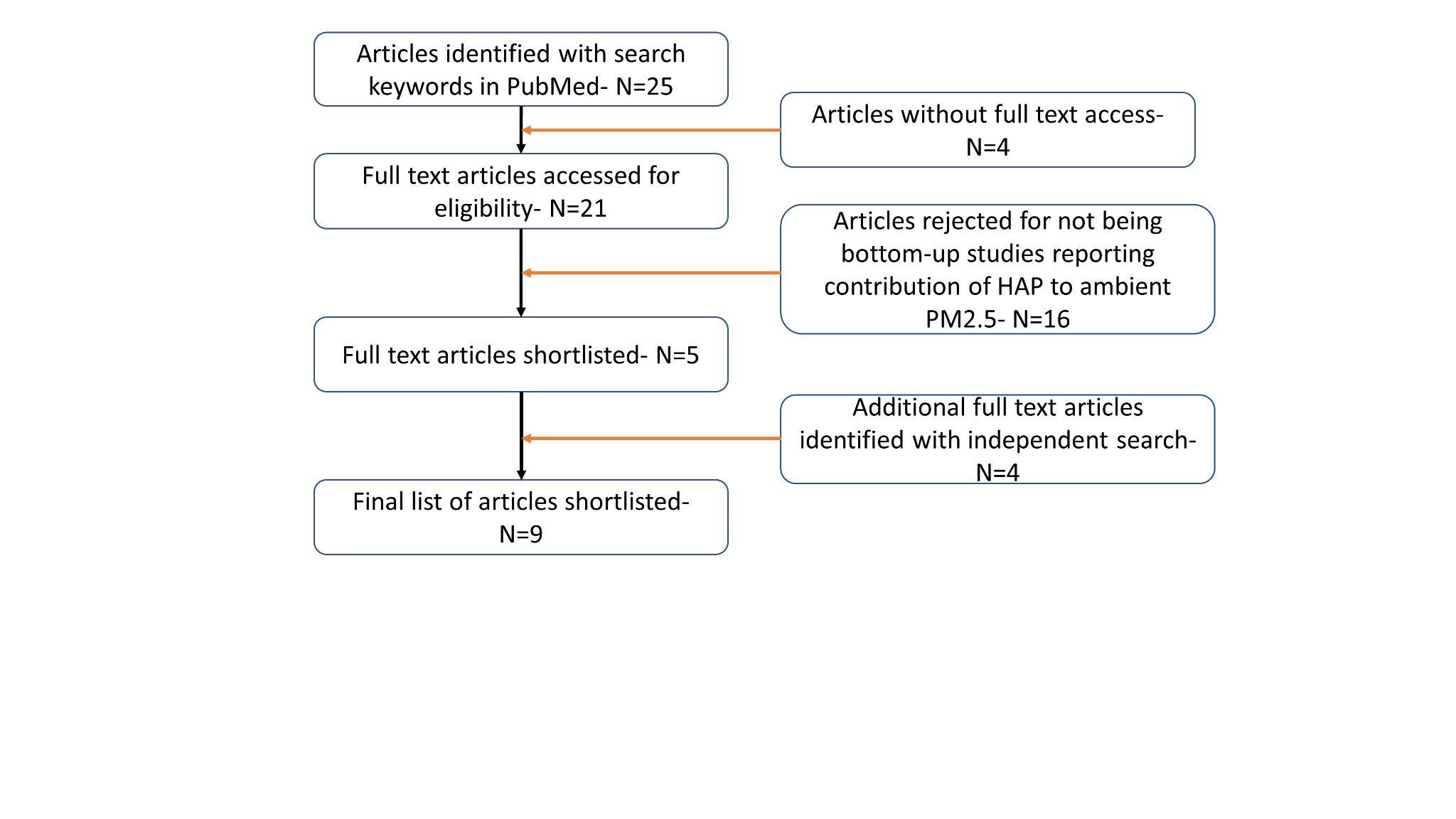
**

Fig S1. Flowchart outlining the criterion adopted to shortlist global studies for this review.

**Supplementary Tables**

Table S1- Information of the studies on contribution of HAP to ambient PM2.5 in East Asia reviewed here

| **Study name** | **Study year** | **Study Focus** | **Model used** | **Horizontal resolution** | **Secondary particle formation** | **Emission Inventory** | **Anthropogenic Emission Sources** | **Residential Emission** | **% of ambient PM2.5 from HAP** | **Associated premature mortality (in million, 95% CI)** | **ERF used** |
| --- | --- | --- | --- | --- | --- | --- | --- | --- | --- | --- | --- |
| Liu2016 [(Liu et al., 2016)](https://www.zotero.org/google-docs/?dgHV3G) | 2010 | Beijing, Tianjin and Hebei (BTH) | WRF-Chem | 36km x 36km | Yes | MEIC | power, industry, transportation,residential, and agriculture | Residential and other sectors | 22 | NA | NA |
| GBDMAPS2016 [(GBD MAPS working group, 2016)](https://www.zotero.org/google-docs/?oTnGgS) | 2013 | China | GEOS-Chem | 0.5° × 0.67° | Yes | MICS Asia III | Power plant coal,Industrial coal,Domestic coal,Noncoal industrial, Domestic biomass burning,Open burning, traffic, solvent use | Domestic Biomass and coal use for heating and cooking | 19 | 0.17 | IER |
| Timmermans2017 [(Timmermans et al., 2017)](https://www.zotero.org/google-docs/?LuaYhy) | 2013 | Beijing, Shanghai | LOTOS-EUROS | 21km x 15km | No | EDGAR v2.4 | Energy, residential combustion, industry, transport, agriculture | Space heating, cooking, emission from local and commercial energy use from small combustion sources, diesel generator sets | 33.2 (Beijing), 21.4(Shanghai) | NA | NA |
| Aunan2018 [(Aunan et al., 2018)](https://www.zotero.org/google-docs/?YLEMfH) | 2010-2013 | China | GEOS-Chem | 0.5° × 0.67 | Yes | MICS-ASIA | power, industry, transportation,residential, and agriculture | Residential and other sectors | 62-74% to IPWE | 1.15 | IER |
| Zhao2018 [(Zhao et al., 2018)](https://www.zotero.org/google-docs/?6aWARg) | 2005, 2015 | China | CMAQ+IPWE | 36- × 36-km | Yes | MICS-ASIA | power, industry, transportation,residential, and agriculture | Residential and other sectors | 61 to IPWE in 2005, ~30 to IPWE in 2015 | 0.91 in 2005, 0.64 in 2015 | IER |
| Shen2019 [(Shen et al., 2019)](https://www.zotero.org/google-docs/?1Trb5P) | 1992, 2012 | China | WRF-Chem | 50km x 50km | Yes | PKU emission database | Energy production, industry, transportation, residential & commercial, agriculture and deforestation | Residential and other sectors | 33 in 1992,10 in 2012 | 0.57in 1992;  0.21 in 2010 | GEMM |
| Reddington2019 [(Reddington et al., 2019)](https://www.zotero.org/google-docs/?bQGI4a) | 2014 | China, South and SouthEast Asia | WRF-Chem | 50km x 50km | Yes | EDGAR-HTAPv2.2 | Agricultural, power generation, industrial non power, residential energy use and land transport. | Small scale supplemental engines for residential, commercial, agricultural, solid waste and wastewater treatment plants, Cooking, space heating water heating, lighting | 38 | 0.18 | IER |
| Yun2020[(Yun et al., 2020)](https://www.zotero.org/google-docs/?wJvaHr) | 2014 | China | WRF-Chem | 50km x 50km | Yes | PKU emission database | Energy production, industry, transportation, residential & commercial, agriculture and deforestation | Residential and other sectors | 68% (AAP+HAP exposure) | 0.77 (0.43-1.17) | IER |
| Chafe2014 [(Chafe et al., 2014)](https://www.zotero.org/google-docs/?CtEAyF) | 2010 | Global/East Asia | TM5-FASST | TM5-FASST regions | Yes | GAINS | industry, land transport, residential and commercial energy, power generation, biomass burning, agriculture | Cooking | 23 | 0.13 | IER |
| Lelieveld2015 [(Lelieveld et al., 2015)](https://www.zotero.org/google-docs/?jfBKVG) | 2010 | Global/China | ECHAM5/MESSy | 1.1º x 1.1º | Yes | EDGAR,2010 | industry, land transport, residential and commercial energy, power generation, biomass burning, agriculture | Space heating, cooking, emission from local and commercial energy use from small combustion sources, diesel generator sets | 32 | 0.43 | IER |
| Butt2016 [(Butt et al., 2016)](https://www.zotero.org/google-docs/?Z03jYJ) | 2000 | Global/China | GLOMAP | 2.8º x 2.8º | Yes | Various sources | Energy sources and distribution, industry, land transport, maritime transport, residential and commercial, agricultural waste burning | Space heating, cooking, emission from local and commercial energy use from small combustion sources | 13 | 0.1 | log-linear |
| Silva2016 [(Silva et al., 2016)](https://www.zotero.org/google-docs/?iPYuCz) | 2005 | Global/East Asia | MOZART-4 | 0.5º x 0.67º | Yes | RCP,2005 | Residential and commercial, energy, industry, land transport, shipping and aviation | Space heating, cooking, emission from local and commercial energy use from small combustion sources | 21 | 0.23 | IER |
| Karagulian2016 [(Karagulian et al., 2017)](https://www.zotero.org/google-docs/?hOySz3) | 2010 | Global/China | TM5-FASST | 1°x1° | Yes | EDGAR-HTAP, 2010 | Agricultural, power generation, industrial non power, residential energy use and land transport. | Small scale supplemental engines for residential, commercial, agricultural, solid waste and wastewater treatment plants, Cooking, space heating water heating, lighting | 26.7 | NA | NA |
| Crippa2019 [(Crippa et al., 2019)](https://www.zotero.org/google-docs/?3Ujc44) | 2010 | Global/China | TM5-FASST | 1°x1° | Yes | EDGAR-HTAP, 2010 | Agricultural, power generation, industrial non power, residential energy use and land transport. | Small scale supplemental engines for residential, commercial, agricultural, solid waste and wastewater treatment plants, Cooking, space heating water heating, lighting | 23.1 | 0.15 | IER |
| McDuffie2021 [(McDuffie et al., 2021)](https://www.zotero.org/google-docs/?nXbNbo) | 2019 | Global/East Asia | GEOSChem, satellite | 2° × 2.5° globally, 0.5° × 0.625° over North America, Europe and Asia. | Yes | CEDS,2017 | Agricultural soils, agricultural waste burning, residential, industry,power generation, ships, transportation | residential heating and cooking (coal, biofuel and others) | 13 | 0.18 | MR-BRT |
| Chowdhury2022 [(Chowdhury et al., 2022)](https://www.zotero.org/google-docs/?4NG39U) | 2015 | ECHAM5/MESSy | ERA5 | 1.1º x 1.1º | Yes | CEDS,2014 | Agricultural soils, agricultural waste burning, residential, industry,power generation, ships, transportation | residential heating and cooking,commercial and institutional combustion,combustion from agriculture, forestry, and fishing | 20.8 (33)^^[[1]](#footnote-1)^^ | 0.32(0.51) | MR-BRT |

Table S2- Information of the studies on contribution of HAP to ambient PM2.5 in South Asia reviewed here

| **Study name** | **Study year** | **Study Focus** | **Model used** | **Horizontal resolution** | **Secondary particle formation** | **Emission Inventory** | **Anthropogenic Emission Sources** | **Residential Emission** | **% of ambient PM2.5 from HAP** | **Associated premature mortality (in million, 95% CI)** | **ERF used** |
| --- | --- | --- | --- | --- | --- | --- | --- | --- | --- | --- | --- |
| GBDMAPS2018 [(MAPS Working Group, 2018)](https://www.zotero.org/google-docs/?qnrHPM) | 2015 | India | GEOS-Chem | 0.5º x 0.67º | Yes | SMOG | Residential biomass, total coal, industrial coal, powerplant coal, open burning, transportation, brick production, distributed diesel, anthropogenic dust | Cooking, space heating water heating, lighting | 23.9 | 0.27(0.23-0.3) | IER |
| Guo2017 [(Guo et al., 2017)](https://www.zotero.org/google-docs/?dU31KD) | 2015 | Northern India | CMAQ | 12km x 12km | Yes | EDGAR 4.3 | industry, land transport, residential and commercial energy, power generation, biomass burning, agriculture | Space heating, cooking, emission from local and commercial energy use from small combustion sources, diesel generator sets | ~40% | NA | NA |
| Conibear2018 [(Conibear et al., 2018)](https://www.zotero.org/google-docs/?znq3Xn) | 2014 | India | WRF-Chem | 30km x 30km | Yes | EDGAR-HTAP | Agricultural, biomass burning, dust, power generation, industrial non power, residential energy use and land transport. | Small scale supplemental engines for residential, commercial, agricultural, solid waste and wastewater treatment plants, Cooking, space heating water heating, lighting | 29.5 | 0.25(0.16-0.34) | IER |
| Sharma2018 [(Sharma et al., 2018)](https://www.zotero.org/google-docs/?FOx4eN) | 2016 | Delhi/India | CMAQ | 4km x 4km | Yes | Various sources | Residential, Open agricultural residue burning, Transport – tailpipe, Construction, Industries (including bricks), Power plants- stacks and fly ash ponds,Road dust, Diesel generators, Refuse burning, Crematoria, Restaurants-hotels,Airport, Landfills, Waste incinerators, Solvents, and Ammonia emissions | Space heating, cooking, emission, lighting | 10 | NA | NA |
| Upadhyay2018 [(Upadhyay et al., 2018)](https://www.zotero.org/google-docs/?9aENNo) | 2010 | India | WRF-Chem | 10km x 10km | Yes | EDGAR-HTAP | Agricultural, biomass burning, dust, power generation, industrial non power, residential energy use and land transport. | Small scale supplemental engines for residential, commercial, agricultural, solid waste and wastewater treatment plants, Cooking, space heating water heating, lighting | 70 | 0.37(0.17-0.57) | IER |
| Chowdhury2019 [(Chowdhury et al., 2019b)](https://www.zotero.org/google-docs/?uDpsl1) | 2015 | India | WRF-CAMx | 0.25º x 0.25º | Yes | Various sources | Residential (cooking, heating, and lighting), all industries (including power, bricks), all transport (road, rail, air, and water), natural and resuspended dust, open fires, seasalt and lightning, open waste burning, and some miscellaneous sources | Cooking, space heating, water heating, lighting | 29.6 | 0.26(0.15-0.37) | IER |
| Chafe2014 [(Chafe et al., 2014)](https://www.zotero.org/google-docs/?NjTFXq) | 2010 | Global/South Asia | TM5-FASST | TM5-FASST regions | Yes | GAINS | industry, land transport, residential and commercial energy, power generation, biomass burning, agriculture | Cooking | 26 | 0.2 | IER |
| Lelieveld2015 [(Lelieveld et al., 2015)](https://www.zotero.org/google-docs/?Wgr14T) | 2010 | Global/India | ECHAM5/MESSy | 1.1º x 1.1º | Yes | EDGAR,2010 | industry, land transport, residential and commercial energy, power generation, biomass burning, agriculture | Space heating, cooking, emission from local and commercial energy use from small combustion sources, diesel generator sets | 50 | 0.32 | IER |
| Butt2016 [(Butt et al., 2016)](https://www.zotero.org/google-docs/?akuRDA) | 2000 | Global/South Asia | GLOMAP | 2.8º x 2.8º | Yes | Various sources | Energy sources and distribution, industry, land transport, maritime transport, residential and commercial, agricultural waste burning | Space heating, cooking, emission from local and commercial energy use from small combustion sources | 22 | 0.07 | log-linear |
| Silva2016 [(Silva et al., 2016)](https://www.zotero.org/google-docs/?kG63lP) | 2005 | Global/India | MOZART-4 | 0.5º x 0.67º | Yes | RCP,2005 | Residential and commercial, energy, industry, land transport, shipping and aviation | Space heating, cooking, emission from local and commercial energy use from small combustion sources | 42.8 | 0.19 | IER |
| Karagulian2016 [(Karagulian et al., 2017)](https://www.zotero.org/google-docs/?pa47Cv) | 2010 | Global/China | TM5-FASST | 1°x1° | Yes | EDGAR-HTAP, 2010 | Agricultural, power generation, industrial non power, residential energy use and land transport. | Small scale supplemental engines for residential, commercial, agricultural, solid waste and wastewater treatment plants, Cooking, space heating water heating, lighting | 42 | NA | NA |
| Crippa2019 [(Crippa et al., 2019)](https://www.zotero.org/google-docs/?PI8ZkJ) | 2010 | Global/India | TM5-FASST | 1°x1° | Yes | EDGAR-HTAP, 2010 | Agricultural, power generation, industrial non power, residential energy use and land transport. | Small scale supplemental engines for residential, commercial, agricultural, solid waste and wastewater treatment plants, Cooking, space heating water heating, lighting | 45.2 | 0.27 | IER |
| McDuffie2021 [(McDuffie et al., 2021)](https://www.zotero.org/google-docs/?b6rNjZ) | 2017 | Global/South Asia | GEOSChem, satellite | 2° × 2.5° globally, 0.5° × 0.625° over North America, Europe and Asia. | Yes | CEDS,2017 | Agricultural soils, agricultural waste burning, residential, industry,power generation, ships, transportation | residential heating and cooking (coal, biofuel and others) | 18 | 0.19 | MR-BRT |
| Chowdhury2022 [(Chowdhury et al., 2022)](https://www.zotero.org/google-docs/?i7TheV) | 2015 | ECHAM5/MESSy | ERA5 | 1.1º x 1.1º | Yes | CEDS,2014 | Agricultural soils, agricultural waste burning, residential, industry,power generation, ships, transportation | residential heating and cooking,commercial and institutional combustion,combustion from agriculture, forestry, and fishing | 24.3 (41.8) | 0.28(0.49) | MR-BRT |

TableS3- Information of the studies on contribution of HAP to ambient PM2.5 in Europe reviewed here

| **Study name** | **Study year** | **Study Focus** | **Model used** | **Horizontal resolution** | **Secondary particle formation** | **Emission Inventory** | **Anthropogenic Emission Sources** | **Residential Emission** | **% of ambient PM2.5 from HAP** | **Associated premature mortality (in million, 95% CI)** | **ERF used** |
| --- | --- | --- | --- | --- | --- | --- | --- | --- | --- | --- | --- |
| Hendriks2013[(Hendriks et al., 2013)](https://www.zotero.org/google-docs/?rJA5yy) | 2007-2009 | Europe/Netherlands | LOTOS-EUROS | 7km x 7km | Yes | TNO-MACC | road transport, other transport, agri-culture, power generation,residential combustion and industry. | Domestic heating and other activities | ~5% | NA | NA |
| Pirovono2015 [(Pirovano et al., 2015)](https://www.zotero.org/google-docs/?kO6Coy) | 2005 | Europe/Italy/Lombardy | CAMx | 5km x 5km | Yes | Various sources | Power plant, industry, waste treatment, residential, transportation, agriculture | Domestic heating and other activities | 6.6% in summer,  50.8% in winter | NA | NA |
| Karamchandani 2017 [(Karamchandani et al., 2017)](https://www.zotero.org/google-docs/?lw9nVf) | 2010 | Europe/16 cities | CAMx | 23km x 23km | Yes | TNO-MACC_II | Energy, residential, industry, solvent, Extraction and distribution of fossil fuels, Solvent and other product use, Road transport, Non-road transport, Waste treatment, agriculture | Domestic heating and other activities | <5 -11% in summer,  11-47% in winter | NA | NA |
| Thunis2018[(Thunis et al., 2018)](https://www.zotero.org/google-docs/?8WW7c5) | 2010 | Europe/150 cities | SHERPA-CHIMERE | 7km x 7km | Yes | GAINS | Power plant, industry, waste treatment, residential, transportation, agriculture | combustion infire-places, medium and single-house boilers, cooking and heating stovesin commercial, institutional and residential activities | 13% in 150 cities | NA | NA |
| Kukkonen2020/Orru2022 [(Kukkonen et al., 2020)](https://www.zotero.org/google-docs/?dwaRd9) | 2013 | Europe/ 4 cities in Scandinavia | Different models for 4 cities [(Kukkonen et al., 2020)](https://www.zotero.org/google-docs/?95pUIh) | Different resolution for different cities. | Varies across models | Emission inventories constructed for cities. [(Kukkonen et al., 2020)](https://www.zotero.org/google-docs/?t48lzI) | [(Kukkonen et al., 2020)](https://www.zotero.org/google-docs/?CAYNee) | [(Kukkonen et al., 2020)](https://www.zotero.org/google-docs/?H1JHSu) | 0 % to 15 %, from 0 % to 20 %, from 8 % to 22 % and from 0 % to 60 % in Helsinki, Copenhagen, Umeå and Oslo | 19 premature deaths in Umeå (95% CI: 8–29), 85 in the Helsinki Metropolitan Area (95% CI: 35–129), 78 in Copenhagen (95% CI: 33–118), and 232 premature deaths in Oslo (95% CI: 97–346) [(Orru et al., 2022)](https://www.zotero.org/google-docs/?BuHYAg) | Log-linear [(Hvidtfeldt et al., 2019; Turner et al., 2015)](https://www.zotero.org/google-docs/?TUwmKf) |
| Chafe2014 [(Chafe et al., 2014)](https://www.zotero.org/google-docs/?BY7ae9) | 2010 | Global/Europe | TM5-FASST | TM5-FASST regions | Yes | GAINS | industry, land transport, residential and commercial energy, power generation, biomass burning, agriculture | Cooking | 0 | 0 | IER |
| Lelieveld2015 [(Lelieveld et al., 2015)](https://www.zotero.org/google-docs/?keg7jD) | 2010 | Global/India | ECHAM5/MESSy | 1.1º x 1.1º | Yes | EDGAR,2010 | industry, land transport, residential and commercial energy, power generation, biomass burning, agriculture | Space heating, cooking, emission from local and commercial energy use from small combustion sources, diesel generator sets | 5-17% | 0.05 | IER |
| Butt2016 [(Butt et al., 2016)](https://www.zotero.org/google-docs/?eJgqPC) | 2000 | Global/South Asia | GLOMAP | 2.8º x 2.8º | Yes | Various sources | Energy sources and distribution, industry, land transport, maritime transport, residential and commercial, agricultural waste burning | Space heating, cooking, emission from local and commercial energy use from small combustion sources | 15-40% in Eastern Europe, <10% in rest of Europe | 0.06 | log-linear |
| Silva2016 [(Silva et al., 2016)](https://www.zotero.org/google-docs/?g94RbJ) | 2005 | Global/India | MOZART-4 | 0.5º x 0.67º | Yes | RCP,2005 | Residential and commercial, energy, industry, land transport, shipping and aviation | Space heating, cooking, emission from local and commercial energy use from small combustion sources | 11.1 | 0.06(0.04-0.08) | IER |
| Karagulian2016 [(Karagulian et al., 2017)](https://www.zotero.org/google-docs/?pNiyoR) | 2010 | Global/China | TM5-FASST | 1°x1° | Yes | EDGAR-HTAP, 2010 | Agricultural, power generation, industrial non power, residential energy use and land transport. | Small scale supplemental engines for residential, commercial, agricultural, solid waste and wastewater treatment plants, Cooking, space heating water heating, lighting | 25% in Central Europe, 12% in Western Europe and East Europe | NA | NA |
| Crippa2019 [(Crippa et al., 2019)](https://www.zotero.org/google-docs/?iZkxSw) | 2010 | Global/India | TM5-FASST | 1°x1° | Yes | EDGAR-HTAP, 2010 | Agricultural, power generation, industrial non power, residential energy use and land transport. | Small scale supplemental engines for residential, commercial, agricultural, solid waste and wastewater treatment plants, Cooking, space heating water heating, lighting | 19.7 | 0.05(0.03-0.07) | IER |
| McDuffie2021 [(McDuffie et al., 2021)](https://www.zotero.org/google-docs/?pVmVE9) | 2017 | Global/South Asia | GEOSChem, satellite | 2° × 2.5° globally, 0.5° × 0.625° over North America, Europe and Asia. | Yes | CEDS,2017 | Agricultural soils, agricultural waste burning, residential, industry,power generation, ships, transportation | residential heating and cooking (coal, biofuel and others) | ~20% in Central Europe, 10-12% in Western and Eastern Europe | 0.04 | MR-BRT |
| Chowdhury2022 [(Chowdhury et al., 2022)](https://www.zotero.org/google-docs/?dYjmQr) | 2015 | ECHAM5/MESSy | ERA5 | 1.1º x 1.1º | Yes | CEDS,2014 | Agricultural soils, agricultural waste burning, residential, industry,power generation, ships, transportation | residential heating and cooking,commercial and institutional combustion,combustion from agriculture, forestry, and fishing | 17.8(23.6)% in Eastern Europe, 16(19)% in Northern Europe, 19(26)% in Western Europe | 0.04(0.05) in Eastern Europe, 0.003(0.005) in Northern Europe, 0.01(0.015) in Western Europe | MR-BRT |

Table S4- Information of the studies on contribution of HAP to ambient PM2.5 in Africa reviewed here

| **Study name** | **Study year** | **Study Focus** | **Model used** | **Horizontal resolution** | **Secondary particle formation** | **Emission Inventory** | **Anthropogenic Emission Sources** | **Residential Emission** | **% of ambient PM2.5 from HAP** | **Associated premature mortality (in million, 95% CI)** | **ERF used** |
| --- | --- | --- | --- | --- | --- | --- | --- | --- | --- | --- | --- |
| Chafe2014 [(Chafe et al., 2014)](https://www.zotero.org/google-docs/?kxgxpb) | 2010 | Global/Africa | TM5-FASST | TM5-FASST regions | Yes | GAINS | industry, land transport, residential and commercial energy, power generation, biomass burning, agriculture | Cooking | 37%, 13%, 10% and 9.8% in Southern,Eastern, Western and Central sub-Saharan Africa | 0.0014,0.0035,0.0078 and 0.0016 in Southern,Eastern, Western and Central sub-Saharan Africa | IER |
| Lelieveld2015 [(Lelieveld et al., 2015)](https://www.zotero.org/google-docs/?EEhgwR) | 2010 | Global/Africa | ECHAM5/MESSy | 1.1º x 1.1º | Yes | EDGAR,2010 | industry, land transport, residential and commercial energy, power generation, biomass burning, agriculture | Space heating, cooking, emission from local and commercial energy use from small combustion sources, diesel generator sets | ~10% | 0.023 | IER |
| Butt2016 [(Butt et al., 2016)](https://www.zotero.org/google-docs/?llYybt) | 2000 | Global/ sub-Saharan Africa | GLOMAP | 2.8º x 2.8º | Yes | Various sources | Energy sources and distribution, industry, land transport, maritime transport, residential and commercial, agricultural waste burning | Space heating, cooking, emission from local and commercial energy use from small combustion sources | ~5% | 0.005 | log-linear |
| Silva2016 [(Silva et al., 2016)](https://www.zotero.org/google-docs/?wAZRu1) | 2005 | Global/Africa | MOZART-4 | 0.5º x 0.67º | Yes | RCP,2005 | Residential and commercial, energy, industry, land transport, shipping and aviation | Space heating, cooking, emission from local and commercial energy use from small combustion sources | 43% | 0.012(0.002-0.023) | IER |
| Karagulian2017 [(Karagulian et al., 2017)](https://www.zotero.org/google-docs/?HOiaZF) | 2010 | Global/Africa | TM5-FASST | 1°x1° | Yes | EDGAR-HTAP, 2010 | Agricultural, power generation, industrial non power, residential energy use and land transport. | Small scale supplemental engines for residential, commercial, agricultural, solid waste and wastewater treatment plants, Cooking, space heating water heating, lighting | 15% | NA | NA |
| Crippa2019 [(Crippa et al., 2019)](https://www.zotero.org/google-docs/?q3VXGS) | 2010 | Global/Africa | TM5-FASST | 1°x1° | Yes | EDGAR-HTAP, 2010 | Agricultural, power generation, industrial non power, residential energy use and land transport. | Small scale supplemental engines for residential, commercial, agricultural, solid waste and wastewater treatment plants, Cooking, space heating water heating, lighting | 38% | 0.028(0.012-0.06) | IER |
| McDuffie2021 [(McDuffie et al., 2021)](https://www.zotero.org/google-docs/?JpT60C) | 2017 | Global/Africa | GEOSChem, satellite | 2° × 2.5° globally, 0.5° × 0.625° over North America, Europe and Asia. | Yes | CEDS,2017 | Agricultural soils, agricultural waste burning, residential, industry,power generation, ships, transportation | residential heating and cooking (coal, biofuel and others) | 20%, 25%, 10% and 10% in Southern,Eastern, Western and Central sub-Saharan Africa | 0.006,0.008,0.01 and 0.002 in Southern,Eastern, Western and Central sub-Saharan Africa | MR-BRT |
| Chowdhury2022[(Chowdhury et al., 2022)](https://www.zotero.org/google-docs/?NgGExP) | 2015 | ECHAM5/MESSy | ERA5 | 1.1º x 1.1º | Yes | CEDS,2014 | Agricultural soils, agricultural waste burning, residential, industry,power generation, ships, transportation | residential heating and cooking,commercial and institutional combustion,combustion from agriculture, forestry, and fishing | 25.7(39.8), 10.8(15), 4.3(6.1), 27.8(41), 22.8(39) in East Africa, Central Africa, North Africa, Southern Africa and West Africa respectively | 0.02(0.03), 0.005(008), 0.006(0.007), 0.004(0.005) and 0.4(0.67) in East Africa, Central Africa, North Africa, Southern Africa and West Africa | MR-BRT |

Table S5- Information of the studies on contribution of HAP to ambient PM2.5 in Latin America reviewed here

| **Study name** | **Study year** | **Study Focus** | **Model used** | **Horizontal resolution** | **Secondary particle formation** | **Emission Inventory** | **Anthropogenic Emission Sources** | **Residential Emission** | **% of ambient PM2.5 from HAP** | **Associated premature mortality (in million, 95% CI)** | **ERF used** |
| --- | --- | --- | --- | --- | --- | --- | --- | --- | --- | --- | --- |
| Chafe2014 [(Chafe et al., 2014)](https://www.zotero.org/google-docs/?0hd2g0) | 2010 | Global/Latin America | TM5-FASST | TM5-FASST regions | Yes | GAINS | industry, land transport, residential and commercial energy, power generation, biomass burning, agriculture | Cooking | 15% in Southern Latin America, 7.9% in Tropical Latin America, 5.7% in Andean Latin America, 5.3% in Central Latin America, 5.3% in Caribbeans | 500 in Southern Latin America, 540 in Tropical Latin America, 160 in Andean Latin America and 1400 in Central Latin America, 380in Caribbeans | IER |
| Lelieveld2015 [(Lelieveld et al., 2015)](https://www.zotero.org/google-docs/?CUbc5B) | 2010 | Global/Latin America | ECHAM5/MESSy | 1.1º x 1.1º | Yes | EDGAR,2010 | industry, land transport, residential and commercial energy, power generation, biomass burning, agriculture | Space heating, cooking, emission from local and commercial energy use from small combustion sources, diesel generator sets | <5% | 0.003 | IER |
| Butt2016 [(Butt et al., 2016)](https://www.zotero.org/google-docs/?utIMSy) | 2000 | Global/Latin America | GLOMAP | 2.8º x 2.8º | Yes | Various sources | Energy sources and distribution, industry, land transport, maritime transport, residential and commercial, agricultural waste burning | Space heating, cooking, emission from local and commercial energy use from small combustion sources | <5% | 0.002 | log-linear |
| Silva2016 [(Silva et al., 2016)](https://www.zotero.org/google-docs/?IfNIxP) | 2005 | Global/South America | MOZART-4 | 0.5º x 0.67º | Yes | RCP,2005 | Residential and commercial, energy, industry, land transport, shipping and aviation | Space heating, cooking, emission from local and commercial energy use from small combustion sources | 5.9% | 0.006(0.001-0.02 | IER |
| Karagulian201[(Karagulian et al., 2017)](https://www.zotero.org/google-docs/?QDW7Lu) | 2010 | Global/South America | TM5-FASST | 1°x1° | Yes | EDGAR-HTAP, 2010 | Agricultural, power generation, industrial non power, residential energy use and land transport. | Small scale supplemental engines for residential, commercial, agricultural, solid waste and wastewater treatment plants, Cooking, space heating water heating, lighting | <5% | NA | NA |
| Crippa2019 [(Crippa et al., 2019)](https://www.zotero.org/google-docs/?fEAubD) | 2010 | Global/South America | TM5-FASST | 1°x1° | Yes | EDGAR-HTAP, 2010 | Agricultural, power generation, industrial non power, residential energy use and land transport. | Small scale supplemental engines for residential, commercial, agricultural, solid waste and wastewater treatment plants, Cooking, space heating water heating, lighting | 18.9 | 0.005(0.001-0.005) | IER |
| McDuffie2021 [(McDuffie et al., 2021)](https://www.zotero.org/google-docs/?gqHxON) | 2017 | Global/Latin America | GEOSChem, satellite | 2° × 2.5° globally, 0.5° × 0.625° over North America, Europe and Asia. | Yes | CEDS,2017 | Agricultural soils, agricultural waste burning, residential, industry,power generation, ships, transportation | residential heating and cooking (coal, biofuel and others) | 15.5% in Southern Latin America, 7% in Tropical Latin America, 8.5% in Andean Latin America, 15% in Central Latin America, 20.6% in Caribbeans | 0.003 in Southern Latin America, 0.003 in Tropical Latin America, 0.001 in Andean Latin America, 0.01 in Central Latin America, and 0.003 in Caribbeans | MR-BRT |
| Chowdhury2022 [(Chowdhury et al., 2022)](https://www.zotero.org/google-docs/?wfrnC0) | 2015 | ECHAM5/MESSy | ERA5 | 1.1º x 1.1º | Yes | CEDS,2014 | Agricultural soils, agricultural waste burning, residential, industry,power generation, ships, transportation | residential heating and cooking,commercial and institutional combustion,combustion from agriculture, forestry, and fishing | 7.36(11.43)% | 0.004(0.008) | MR-BRT |

Table S6- Information of the studies on contribution of HAP to ambient PM2.5 in North America reviewed here

| **Study name** | **Study year** | **Study Focus** | **Model used** | **Horizontal resolution** | **Secondary particle formation** | **Emission Inventory** | **Anthropogenic Emission Sources** | **Residential Emission** | **% of ambient PM2.5 from HAP** | **Associated premature mortality (in million, 95% CI)** | **ERF used** |
| --- | --- | --- | --- | --- | --- | --- | --- | --- | --- | --- | --- |
| Chafe2014 [(Chafe et al., 2014)](https://www.zotero.org/google-docs/?6eZDtt) | 2010 | Global/North America | TM5-FASST | TM5-FASST regions | Yes | GAINS | industry, land transport, residential and commercial energy, power generation, biomass burning, agriculture | Cooking | 0 | 0 | IER |
| Lelieveld2015 [(Lelieveld et al., 2015)](https://www.zotero.org/google-docs/?TjV7bk) | 2010 | Global/North America | ECHAM5/MESSy | 1.1º x 1.1º | Yes | EDGAR,2010 | industry, land transport, residential and commercial energy, power generation, biomass burning, agriculture | Space heating, cooking, emission from local and commercial energy use from small combustion sources, diesel generator sets | 6% | 0.003 | IER |
| Butt2016 [(Butt et al., 2016)](https://www.zotero.org/google-docs/?ZKLBME) | 2000 | Global/North America | GLOMAP | 2.8º x 2.8º | Yes | Various sources | Energy sources and distribution, industry, land transport, maritime transport, residential and commercial, agricultural waste burning | Space heating, cooking, emission from local and commercial energy use from small combustion sources | <5% | 0.002 | log-linear |
| Silva2016 [(Silva et al., 2016)](https://www.zotero.org/google-docs/?AIIa0b) | 2005 | Global/North America | MOZART-4 | 0.5º x 0.67º | Yes | RCP,2005 | Residential and commercial, energy, industry, land transport, shipping and aviation | Space heating, cooking, emission from local and commercial energy use from small combustion sources | 10.5 | 0.01(0.001-0.02 | IER |
| Karagulian2017 [(Karagulian et al., 2017)](https://www.zotero.org/google-docs/?Kv8TTA) | 2010 | Global/North America | TM5-FASST | 1°x1° | Yes | EDGAR-HTAP, 2010 | Agricultural, power generation, industrial non power, residential energy use and land transport. | Small scale supplemental engines for residential, commercial, agricultural, solid waste and wastewater treatment plants, Cooking, space heating water heating, lighting | <5% | NA | NA |
| Crippa2019 [(Crippa et al., 2019)](https://www.zotero.org/google-docs/?FpF8cC) | 2010 | Global/North America | TM5-FASST | 1°x1° | Yes | EDGAR-HTAP, 2010 | Agricultural, power generation, industrial non power, residential energy use and land transport. | Small scale supplemental engines for residential, commercial, agricultural, solid waste and wastewater treatment plants, Cooking, space heating water heating, lighting | 15.5 | 0.015 | IER |
| McDuffie2021 [(McDuffie et al., 2021)](https://www.zotero.org/google-docs/?L5dCxC) | 2017 | Global/North America | GEOSChem, satellite | 2° × 2.5° globally, 0.5° × 0.625° over North America, Europe and Asia. | Yes | CEDS,2017 | Agricultural soils, agricultural waste burning, residential, industry,power generation, ships, transportation | residential heating and cooking (coal, biofuel and others) | 7.8% | 0.003 | MR-BRT |
| Chowdhury2022 [(Chowdhury et al., 2022)](https://www.zotero.org/google-docs/?vlQE0j) | 2015 | ECHAM5/MESSy | ERA5 | 1.1º x 1.1º | Yes | CEDS,2014 | Agricultural soils, agricultural waste burning, residential, industry,power generation, ships, transportation | residential heating and cooking,commercial and institutional combustion,combustion from agriculture, forestry, and fishing | 13(19)% in USA and Canada | 0.01(0.014) | MR-BRT |

**References**

[Achakulwisut, P., Brauer, M., Hystad, P., Anenberg, S.C., 2019. Global, national, and urban burdens of paediatric asthma incidence attributable to ambient NO\textlesssub\textgreater2\textless/sub\textgreater pollution: estimates from global datasets. Lancet Planet. Health 3, e166–e178. https://doi.org/10.1016/S2542-5196(19)30046-4](https://www.zotero.org/google-docs/?IgQrmR)

[Anenberg, S.C., Horowitz, L.W., Tong, D.Q., West, J.J., 2010a. An estimate of the global burden of anthropogenic ozone and fine particulate matter on premature human mortality using atmospheric modeling. Environ. Health Perspect. 118, 1189–1195. https://doi.org/10.1289/ehp.0901220](https://www.zotero.org/google-docs/?IgQrmR)

[Anenberg, S.C., Horowitz, L.W., Tong, D.Q., West, J.J., 2010b. An estimate of the global burden of anthropogenic ozone and fine particulate matter on premature human mortality using atmospheric modeling. Environ. Health Perspect. 118, 1189–1195. https://doi.org/10.1289/ehp.0901220](https://www.zotero.org/google-docs/?IgQrmR)

[Apte, J.S., Marshall, J.D., Cohen, A.J., Brauer, M., 2015. Addressing Global Mortality from Ambient PM2.5. Environ. Sci. Technol. 49, 8057–8066. https://doi.org/10.1021/acs.est.5b01236](https://www.zotero.org/google-docs/?IgQrmR)

[Arciszewska, C., McClatchey, J., 2001. The importance of meteorological data for modelling air pollution using ADMS-Urban. Meteorol. Appl. 8, 345–350. https://doi.org/10.1017/S1350482701003103](https://www.zotero.org/google-docs/?IgQrmR)

[Aunan, K., Ma, Q., Lund, M.T., Wang, S., 2018. Population-weighted exposure to PM2.5 pollution in China: An integrated approach. Environ. Int. 120, 111–120. https://doi.org/10.1016/j.envint.2018.07.042](https://www.zotero.org/google-docs/?IgQrmR)

[Balakrishnan, K., Ghosh, S., Ganguli, B., Sambandam, S., Bruce, N., Barnes, D.F., Smith, K.R., 2013. State and national household concentrations of PM2.5 from solid cookfuel use: results from measurements and modeling in India for estimation of the global burden of disease. Environ. Health Glob. Access Sci. Source 12, 77. https://doi.org/10.1186/1476-069X-12-77](https://www.zotero.org/google-docs/?IgQrmR)

[Balmes, J.R., 2019. Household air pollution from domestic combustion of solid fuels and health. J. Allergy Clin. Immunol. 143, 1979–1987. https://doi.org/10.1016/j.jaci.2019.04.016](https://www.zotero.org/google-docs/?IgQrmR)

[Boys, B.L., Martin, R.V., Van Donkelaar, A., MacDonell, R.J., Hsu, N.C., Cooper, M.J., Yantosca, R.M., Lu, Z., Streets, D.G., Zhang, Q., Wang, S.W., 2014. Fifteen-year global time series of satellite-derived fine particulate matter. Environ. Sci. Technol. 48, 11109–11118. https://doi.org/10.1021/es502113p](https://www.zotero.org/google-docs/?IgQrmR)

[Brasseur, G.P., 1997. Formulation of a Chemical Transport Model, in: Brasseur, G.P. (Ed.), The Stratosphere and Its Role in the Climate System, Nato ASI Series. Springer, Berlin, Heidelberg, pp. 313–324. https://doi.org/10.1007/978-3-662-03327-2_18](https://www.zotero.org/google-docs/?IgQrmR)

[Brasseur, G.P., Jacob, D.J., 2017. Modeling of Atmospheric Chemistry. Cambridge University Press, Cambridge. https://doi.org/10.1017/9781316544754](https://www.zotero.org/google-docs/?IgQrmR)

[Burnett, R., Cohen, A., 2020. Relative Risk Functions for Estimating Excess Mortality Attributable to Outdoor PM2.5 Air Pollution: Evolution and State-of-the-Art. Atmosphere 11, 589. https://doi.org/10.3390/atmos11060589](https://www.zotero.org/google-docs/?IgQrmR)

[Burnett, R.T., Popeiii, C.A., Ezzati, M., Olives, C., Lim, S.S., Mehta, S., Shin, H.H., Singh, G., Hubbell, B., Brauer, M., Anderson, H.R., Smith, K.R., Balmes, J.R., Bruce, N.G., Kan, H., Laden, F., Prıfmmode\ddotu\elseü\fiss-Ustıfmmode\ddotu\elseü\fin, A., Turner, M.C., Gapstur, S.M., Diver, W.R., Cohen, A., 2014. An Integrated Risk Function for Estimating the Global Burden of Disease Attributable to Ambient Fine Particulate Matter Exposure. Env. Health Perspect.](https://www.zotero.org/google-docs/?IgQrmR)

[Burnett, R.T., Spadaro, J.V., Garcia, G.R., Pope, C.A., 2022. Designing health impact functions to assess marginal changes in outdoor fine particulate matter. Environ. Res. 204, 112245. https://doi.org/10.1016/j.envres.2021.112245](https://www.zotero.org/google-docs/?IgQrmR)

[Butt, E.W., Rap, A., Schmidt, A., Scott, C.E., Pringle, K.J., Reddington, C.L., Richards, N.A.D., Woodhouse, M.T., Ramirez-Villegas, J., Yang, H., Vakkari, V., Stone, E.A., Rupakheti, M., Praveen, P.S., Van Zyl, P.G., Beukes, J.P., Josipovic, M., Mitchell, E.J.S., Sallu, S.M., Forster, P.M., Spracklen, D.V., 2016. The impact of residential combustion emissions on atmospheric aerosol, human health, and climate. Atmospheric Chem. Phys. 16, 873–905. https://doi.org/10.5194/acp-16-873-2016](https://www.zotero.org/google-docs/?IgQrmR)

[Chafe, Z. a, Brauer, M., Klimont, Z., Van Dingenen, R., Mehta, S., Rao, S., Riahi, K., Dentener, F., Smith, K.R., 2014. Household cooking with solid fuels contributes to ambient PM2.5 air pollution and the burden of disease. Environ. Health Perspect. 122, 1314–20. https://doi.org/10.1289/ehp.1206340](https://www.zotero.org/google-docs/?IgQrmR)

[Chen, J., Hoek, G., 2020. Long-term exposure to PM and all-cause and cause-specific mortality: A systematic review and meta-analysis. Environ. Int. 143, 105974. https://doi.org/10.1016/j.envint.2020.105974](https://www.zotero.org/google-docs/?IgQrmR)

[Chowdhury, S., Dey, S., 2016. Cause-speci fi c premature death from ambient PM 2 . 5 exposure in India : Estimate adjusted for baseline mortality. Environ. Int. 91, 283–290. https://doi.org/10.1016/j.envint.2016.03.004](https://www.zotero.org/google-docs/?IgQrmR)

[Chowdhury, S., Dey, S., Di, L., Smith, K.R., Pillarisetti, A., Lyapustin, A., 2019a. Tracking ambient PM 2 . 5 build-up in Delhi national capital region during the dry season over 15 years using a high-resolution ( 1 km ) satellite aerosol dataset. Atmos. Environ. 204, 142–150. https://doi.org/10.1016/j.atmosenv.2019.02.029](https://www.zotero.org/google-docs/?IgQrmR)

[Chowdhury, S., Dey, S., Guttikunda, S., Pillarisetti, A., Smith, K.R., Di Girolamo, L., 2019b. Indian annual ambient air quality standard is achievable by completely mitigating emissions from household sources. Proc Natl Acad Sci USA 116, 10711–10716. https://doi.org/10.1073/pnas.1900888116](https://www.zotero.org/google-docs/?IgQrmR)

[Chowdhury, S., Dey, S., Guttikunda, S., Pillarisetti, A., Smith, K.R., Girolamo, L.D., 2019c. Indian annual ambient air quality standard is achievable by completely mitigating emissions from household sources. Proc. Natl. Acad. Sci. U. S. A. 166, 10711–10716. https://doi.org/10.1073/pnas.1900888116](https://www.zotero.org/google-docs/?IgQrmR)

[Chowdhury, S., Haines, A., Klingmüller, K., Kumar, V., Pozzer, A., Venkataraman, C., Witt, C., Lelieveld, J., 2021. Global and national assessment of the incidence of asthma in children and adolescents from major sources of ambient NO2. Environ. Res. Lett. 16, 035020. https://doi.org/10.1088/1748-9326/abe909](https://www.zotero.org/google-docs/?IgQrmR)

[Chowdhury, S., Pozzer, A., Dey, S., Klingmueller, K., Lelieveld, J., 2020. Changing risk factors that contribute to premature mortality from ambient air pollution between 2000 and 2015. Env. Res Lett 15, 074010. https://doi.org/10.1088/1748-9326/ab8334](https://www.zotero.org/google-docs/?IgQrmR)

[Chowdhury, S., Pozzer, A., Haines, A., Klingmüller, K., Münzel, T., Paasonen, P., Sharma, A., Venkataraman, C., Lelieveld, J., 2022. Global health burden of ambient PM2.5 and the contribution of anthropogenic black carbon and organic aerosols. Environ. Int. 159, 107020. https://doi.org/10.1016/j.envint.2021.107020](https://www.zotero.org/google-docs/?IgQrmR)

[Cohen, A.J., Brauer, M., Burnett, R., Anderson, H.R., Frostad, J., Estep, K., Balakrishnan, K., Brunekreef, B., Dandona, L., Dandona, R., Feigin, V., Freedman, G., Hubbell, B., Jobling, A., Kan, H., Knibbs, L., Liu, Y., Martin, R., Morawska, L., Pope, C.A., Shin, H., Straif, K., Shaddick, G., Thomas, M., van Dingenen, R., van Donkelaar, A., Vos, T., Murray, C.J.L., Forouzanfar, M.H., 2017. Estimates and 25-year trends of the global burden of disease attributable to ambient air pollution: an analysis of data from the Global Burden of Diseases Study 2015. Lancet 389, 1907–1918. https://doi.org/10.1016/S0140-6736(17)30505-6](https://www.zotero.org/google-docs/?IgQrmR)

[Conibear, L., Butt, E.W., Knote, C., Arnold, S.R., Spracklen, D.V., 2018. Residential energy use emissions dominate health impacts from exposure to ambient particulate matter in India. Nat. Commun. 9, 1–9. https://doi.org/10.1038/s41467-018-02986-7](https://www.zotero.org/google-docs/?IgQrmR)

[Crippa, M., Guizzardi, D., Muntean, M., Schaaf, E., Dentener, F., van Aardenne, J.A., Monni, S., Doering, U., Olivier, J.G.J., Pagliari, V., Janssens-Maenhout, G., 2018. Gridded emissions of air pollutants for the period 1970–2012 within EDGAR v4.3.2. Earth Syst Sci Data 10, 1987–2013. https://doi.org/10.5194/essd-10-1987-2018](https://www.zotero.org/google-docs/?IgQrmR)

[Crippa, M., Janssens-Maenhout, G., Guizzardi, D., Van Dingenen, R., Dentener, F., 2019. Contribution and uncertainty of sectorial and regional emissions to regional and global PM_2.5_ health impacts. Atmospheric Chem. Phys. 19, 5165–5186. https://doi.org/10.5194/acp-19-5165-2019](https://www.zotero.org/google-docs/?IgQrmR)

[Dockery, D.W., Pope III, C.A., 1994. Acute respiratory effects of particulate air pollution, Annual Review of Public Health. https://doi.org/10.1146/annurev.pu.15.050194.000543](https://www.zotero.org/google-docs/?IgQrmR)

[Feser, F., Rockel, B., Storch, H. von, Winterfeldt, J., Zahn, M., 2011. Regional Climate Models Add Value to Global Model Data: A Review and Selected Examples. Bull. Am. Meteorol. Soc. 92, 1181–1192. https://doi.org/10.1175/2011BAMS3061.1](https://www.zotero.org/google-docs/?IgQrmR)

[Gaskins, A.J., Hart, J.E., 2020. The use of personal and indoor air pollution monitors in reproductive epidemiology studies. Paediatr. Perinat. Epidemiol. 34, 513–521. https://doi.org/10.1111/ppe.12599](https://www.zotero.org/google-docs/?IgQrmR)

[GBD MAPS working group, 2016. Burden of Disease Attributable to Coal-Burning and Other Air Pollution Sources in China.](https://www.zotero.org/google-docs/?IgQrmR)

[Guo, H., Kota, S.H., Sahu, S.K., Hu, J., Ying, Q., Gao, A., Zhang, H., 2017. Source apportionment of PM2.5 in North India using source-oriented air quality models. Environ. Pollut. 231, 426–436. https://doi.org/10.1016/j.envpol.2017.08.016](https://www.zotero.org/google-docs/?IgQrmR)

[Hahad, O., Frenis, K., Kuntic, M., Daiber, A., Münzel, T., 2021. Accelerated Aging and Age-Related Diseases (CVD and Neurological) Due to Air Pollution and Traffic Noise Exposure. Int. J. Mol. Sci. 22, 2419. https://doi.org/10.3390/ijms22052419](https://www.zotero.org/google-docs/?IgQrmR)

[Hammer, M.S., van Donkelaar, A., Li, C., Lyapustin, A., Sayer, A.M., Hsu, N.C., Levy, R.C., Garay, M.J., Kalashnikova, O.V., Kahn, R.A., Brauer, M., Apte, J.S., Henze, D.K., Zhang, L., Zhang, Q., Ford, B., Pierce, J.R., Martin, R.V., 2020. Global Estimates and Long-Term Trends of Fine Particulate Matter Concentrations (1998–2018). Env. Sci Technol 54, 7879–7890. https://doi.org/10.1021/acs.est.0c01764](https://www.zotero.org/google-docs/?IgQrmR)

[Hendriks, C., Kranenburg, R., Kuenen, J., van Gijlswijk, R., Wichink Kruit, R., Segers, A., Denier van der Gon, H., Schaap, M., 2013. The origin of ambient particulate matter concentrations in the Netherlands. Atmos. Environ. 69, 289–303. https://doi.org/10.1016/j.atmosenv.2012.12.017](https://www.zotero.org/google-docs/?IgQrmR)

[Hu, R., Wang, S., Aunan, K., Zhao, M., Chen, L., Liu, Z., Hansen, M.H., 2019. Personal exposure to PM2.5 in Chinese rural households in the Yangtze River Delta. Indoor Air 29, 403–412. https://doi.org/10.1111/ina.12537](https://www.zotero.org/google-docs/?IgQrmR)

[Hvidtfeldt, U.A., Sørensen, M., Geels, C., Ketzel, M., Khan, J., Tjønneland, A., Overvad, K., Brandt, J., Raaschou-Nielsen, O., 2019. Long-term residential exposure to PM2.5, PM10, black carbon, NO2, and ozone and mortality in a Danish cohort. Environ. Int. 123, 265–272. https://doi.org/10.1016/j.envint.2018.12.010](https://www.zotero.org/google-docs/?IgQrmR)

[Hystad, P., Duong, M., Brauer, M., Larkin, A., Arku, R., Kurmi, O.P., Fan, W.Q., Avezum, A., Azam, I., Chifamba, J., Dans, A., Plessis, J.L. du, Gupta, R., Kumar, R., Lanas, F., Liu, Z., Lu, Y., Lopez-Jaramillo, P., Mony, P., Mohan, V., Mohan, D., Nair, S., Puoane, T., Rahman, O., Lap, A.T., Wang, Y., Wei, L., Yeates, K., Rangarajan, S., Teo, K., Yusuf, S., null, null, 2019. Health Effects of Household Solid Fuel Use: Findings from 11 Countries within the Prospective Urban and Rural Epidemiology Study. Environ. Health Perspect. 127, 057003. https://doi.org/10.1289/EHP3915](https://www.zotero.org/google-docs/?IgQrmR)

[James, B.S., Shetty, R.S., Kamath, A., Shetty, A., 2020. Household cooking fuel use and its health effects among rural women in southern India—A cross-sectional study. PLOS ONE 15, e0231757. https://doi.org/10.1371/journal.pone.0231757](https://www.zotero.org/google-docs/?IgQrmR)

[Karagulian, F., Van Dingenen, R., Belis, C., Janssens-Maenhout, G., Crippa, M., Guizzardi, D., Dentener, F., 2017. Attribution of anthropogenic PM₂.₅ to emission sources. European Commission. Joint Research Centre. Publications Office of the European Union, LU.](https://www.zotero.org/google-docs/?IgQrmR)

[Karamchandani, P., Long, Y., Pirovano, G., Balzarini, A., Yarwood, G., 2017. Source-sector contributions to European ozone and fine PM in 2010 using AQMEII modeling data. Atmospheric Chem. Phys. 17, 5643–5664. https://doi.org/10.5194/acp-17-5643-2017](https://www.zotero.org/google-docs/?IgQrmR)

[Kukkonen, J., López-Aparicio, S., Segersson, D., Geels, C., Kangas, L., Kauhaniemi, M., Maragkidou, A., Jensen, A., Assmuth, T., Karppinen, A., Sofiev, M., Hellén, H., Riikonen, K., Nikmo, J., Kousa, A., Niemi, J.V., Karvosenoja, N., Santos, G.S., Sundvor, I., Im, U., Christensen, J.H., Nielsen, O.-K., Plejdrup, M.S., Nøjgaard, J.K., Omstedt, G., Andersson, C., Forsberg, B., Brandt, J., 2020. The influence of residential wood combustion on the concentrations of PM_2.5_ in four Nordic cities. Atmospheric Chem. Phys. 20, 4333–4365. https://doi.org/10.5194/acp-20-4333-2020](https://www.zotero.org/google-docs/?IgQrmR)

[Lao, X.Q., Guo, C., Chang, L.-Y., Bo, Y., Zhang, Z., Chuang, Y.C., Jiang, W.K., Lin, C., Tam, T., Lau, A.K.H., Lin, C.-Y., Chan, T.-C., 2019. Long-term exposure to ambient fine particulate matter (PM2.5) and incident type 2 diabetes: a longitudinal cohort study. Diabetologia 62, 759–769. https://doi.org/10.1007/s00125-019-4825-1](https://www.zotero.org/google-docs/?IgQrmR)

[Lelieveld, J., Barlas, C., Giannadaki, D., Pozzer, A., 2013. Model calculated global, regional and megacity premature mortality due to air pollution. Atmos Chem Phys 13, 7023–7037. https://doi.org/10.5194/acp-13-7023-2013](https://www.zotero.org/google-docs/?IgQrmR)

[Lelieveld, J., Evans, J.S., Fnais, M., Giannadaki, D., Pozzer, A., 2015. The contribution of outdoor air pollution sources to premature mortality on a global scale. Nature 525, 367–371. https://doi.org/10.1038/nature15371](https://www.zotero.org/google-docs/?IgQrmR)

[Lelieveld, J., Klingmueller, K., Pozzer, A., Burnett, R.T., Haines, A., Ramanathan, V., 2019. Effects of fossil fuel and total anthropogenic emission removal on public health and climate. Proc Natl Acad Sci USA 116, 7192–7197. https://doi.org/10.1073/pnas.1819989116](https://www.zotero.org/google-docs/?IgQrmR)

[Lewellen, W.S., Sykes, R.I., 1989. Meteorological Data Needs for Modeling Air Quality Uncertainties. J. Atmospheric Ocean. Technol. 6, 759–768. https://doi.org/10.1175/1520-0426(1989)006<0759:MDNFMA>2.0.CO;2](https://www.zotero.org/google-docs/?IgQrmR)

[Li, T., Cao, S., Fan, D., Zhang, Yaqun, Wang, B., Zhao, X., Leaderer, B.P., Shen, G., Zhang, Yawei, Duan, X., 2016. Household concentrations and personal exposure of PM2.5 among urban residents using different cooking fuels. Sci. Total Environ. 0, 6–12. https://doi.org/10.1016/j.scitotenv.2016.01.038](https://www.zotero.org/google-docs/?IgQrmR)

[Liao, J., McCracken, J.P., Piedrahita, R., Thompson, L., Mollinedo, E., Canuz, E., De Léon, O., Díaz-Artiga, A., Johnson, M., Clark, M., Pillarisetti, A., Kearns, K., Naeher, L., Steenland, K., Checkley, W., Peel, J., Clasen, T.F., HAPIN investigators, 2020. The use of bluetooth low energy Beacon systems to estimate indirect personal exposure to household air pollution. J. Expo. Sci. Environ. Epidemiol. 30, 990–1000. https://doi.org/10.1038/s41370-019-0172-z](https://www.zotero.org/google-docs/?IgQrmR)

[Liu, J., Mauzerall, D.L., Chen, Q., Zhang, Q., Song, Y., Peng, W., Klimont, Z., Qiu, X., Zhang, S., Hu, M., Lin, W., Smith, K.R., Zhu, T., 2016. Air pollutant emissions from Chinese households: A major and underappreciated ambient pollution source. Proc. Natl. Acad. Sci. 113, 7756–7761. https://doi.org/10.1073/pnas.1604537113](https://www.zotero.org/google-docs/?IgQrmR)

[MAPS Working Group, G., 2018. Burden of Disease Attributable to Major Air Pollution Sources in India.](https://www.zotero.org/google-docs/?IgQrmR)

[Martin, R.V., Brauer, M., van Donkelaar, A., Shaddick, G., Narain, U., Dey, S., 2019. No one knows which city has the highest concentration of fine particulate matter. Atmospheric Environ. X 3, 100040. https://doi.org/10.1016/j.aeaoa.2019.100040](https://www.zotero.org/google-docs/?IgQrmR)

[McDuffie, E.E., Martin, R.V., Spadaro, J.V., Burnett, R., Smith, S.J., O’Rourke, P., Hammer, M.S., van Donkelaar, A., Bindle, L., Shah, V., Jaeglé, L., Luo, G., Yu, F., Adeniran, J.A., Lin, J., Brauer, M., 2021. Source sector and fuel contributions to ambient PM2.5 and attributable mortality across multiple spatial scales. Nat. Commun. 12, 3594. https://doi.org/10.1038/s41467-021-23853-y](https://www.zotero.org/google-docs/?IgQrmR)

[McDuffie, E.E., Smith, S.J., O’Rourke, P., Tibrewal, K., Venkataraman, C., Marais, E.A., Zheng, B., Crippa, M., Brauer, M., Martin, R.V., 2020. A global anthropogenic emission inventory of atmospheric pollutants from sector- and fuel-specific sources (1970–2017): an application of the Community Emissions Data System (CEDS). Earth Syst. Sci. Data 12, 3413–3442. https://doi.org/10.5194/essd-12-3413-2020](https://www.zotero.org/google-docs/?IgQrmR)

[Mestl, H.E.S., Aunan, K., Seip, H.M., Wang, S., Zhao, Y., Zhang, D., 2007. Urban and rural exposure to indoor air pollution from domestic biomass and coal burning across China. Sci. Total Environ. 377, 12–26. https://doi.org/10.1016/j.scitotenv.2007.01.087](https://www.zotero.org/google-docs/?IgQrmR)

[Münzel, T., Gori, T., Al-Kindi, S., Deanfield, J., Lelieveld, J., Daiber, A., Rajagopalan, S., 2018. Effects of gaseous and solid constituents of air pollution on endothelial function. Eur. Heart J. 39, 3543–3550. https://doi.org/10.1093/eurheartj/ehy481](https://www.zotero.org/google-docs/?IgQrmR)

[Murray, C.J.L., Aravkin, A.Y., Zheng, P., Abbafati, C., Abbas, K.M., Abbasi-Kangevari, M., Abd-Allah, F., Abdelalim, A., Abdollahi, M., Abdollahpour, I., Abegaz, K.H., Abolhassani, H., Aboyans, V., Abreu, Z.-J., Zhao, J.T., Zhao, X.-J.G., Zhao, Y., Zhou, M., Ziapour, A., Zimsen, S.R.M., Brauer, M., Afshin, A., Lim, S.S., 2020. Global burden of 87 risk factors in 204 countries and territories, 1990–2019: a systematic analysis for the Global Burden of Disease Study 2019. Lancet 396, 1223–1249. https://doi.org/10.1016/S0140-6736(20)30752-2](https://www.zotero.org/google-docs/?IgQrmR)

[Naeher, L., Smith, K., Leaderer, B., Mage, D., Grajeda, R., 2000. Indoor and outdoor PM2.5 and CO in high- and low-density Guatemalan villages. J. Expo. Anal. Environ. Epidemiol. 10, 544–51. https://doi.org/10.1038/sj.jea.7500113](https://www.zotero.org/google-docs/?IgQrmR)

[Naeher, L.P., Brauer, M., Lipsett, M., Zelikoff, J.T., Simpson, C.D., Koenig, J.Q., Smith, K.R., 2007. Woodsmoke health effects: A review. Inhal. Toxicol. 19, 67–106. https://doi.org/10.1080/08958370600985875](https://www.zotero.org/google-docs/?IgQrmR)

[Orellano, P., Reynoso, J., Quaranta, N., Bardach, A., Ciapponi, A., 2020. Short-term exposure to particulate matter (PM10 and PM2.5), nitrogen dioxide (NO2), and ozone (O3) and all-cause and cause-specific mortality: Systematic review and meta-analysis. Environ. Int. 142, 105876. https://doi.org/10.1016/j.envint.2020.105876](https://www.zotero.org/google-docs/?IgQrmR)

[Orru, H., Olstrup, H., Kukkonen, J., López-Aparicio, S., Segersson, D., Geels, C., Tamm, T., Riikonen, K., Maragkidou, A., Sigsgaard, T., Brandt, J., Grythe, H., Forsberg, B., 2022. Health impacts of PM2.5 originating from residential wood combustion in four nordic cities. BMC Public Health 22, 1286. https://doi.org/10.1186/s12889-022-13622-x](https://www.zotero.org/google-docs/?IgQrmR)

[Pearson, J.F., Bachireddy, C., Shyamprasad, S., Goldfine, A.B., Brownstein, J.S., 2010. Association Between Fine Particulate Matter and Diabetes Prevalence in the U.S. Diabetes Care 33, 2196–2201. https://doi.org/10.2337/dc10-0698](https://www.zotero.org/google-docs/?IgQrmR)

[Pérez, I.A., García, M.Á., Sánchez, M.L., Pardo, N., Fernández-Duque, B., 2020. Key Points in Air Pollution Meteorology. Int. J. Environ. Res. Public. Health 17, 8349. https://doi.org/10.3390/ijerph17228349](https://www.zotero.org/google-docs/?IgQrmR)

[Pillarisetti, A., Carter, E., Rajkumar, S., Young, B.N., Benka-Coker, M.L., Peel, J.L., Johnson, M., Clark, M.L., 2019. Measuring personal exposure to fine particulate matter (PM2.5) among rural Honduran women: A field evaluation of the Ultrasonic Personal Aerosol Sampler (UPAS). Environ. Int. 123, 50–53. https://doi.org/10.1016/j.envint.2018.11.014](https://www.zotero.org/google-docs/?IgQrmR)

[Pirovano, G., Colombi, C., Balzarini, A., Riva, G.M., Gianelle, V., Lonati, G., 2015. PM2.5 source apportionment in Lombardy (Italy): Comparison of receptor and chemistry-transport modelling results. Atmos. Environ. 106, 56–70. https://doi.org/10.1016/j.atmosenv.2015.01.073](https://www.zotero.org/google-docs/?IgQrmR)

[Platt, S.M., Haddad, I.E., Zardini, A.A., Clairotte, M., Astorga, C., Wolf, R., 2012. Secondary organic aerosol formation from gasoline vehicle emissions in a new mobile environmental reaction chamber 28343–28383. https://doi.org/10.5194/acpd-12-28343-2012](https://www.zotero.org/google-docs/?IgQrmR)

[Pope, C.A., Dockery, D.W., 2006. Health Effects of Fine Particulate Air Pollution: Lines that Connect. J. Air Waste Manag. Assoc. 56, 709–742. https://doi.org/10.1080/10473289.2006.10464485](https://www.zotero.org/google-docs/?IgQrmR)

[Prank, M., Sofiev, M., Tsyro, S., Hendriks, C., Semeena, V., Vazhappilly Francis, X., Butler, T., Denier van der Gon, H., Friedrich, R., Hendricks, J., Kong, X., Lawrence, M., Righi, M., Samaras, Z., Sausen, R., Kukkonen, J., Sokhi, R., 2016. Evaluation of the performance of four chemical transport models in predicting the aerosol chemical composition in Europe in 2005. Atmospheric Chem. Phys. 16, 6041–6070. https://doi.org/10.5194/acp-16-6041-2016](https://www.zotero.org/google-docs/?IgQrmR)

[Reddington, C.L., Conibear, L., Knote, C., Silver, B.J., Li, Y.J., Chan, C.K., Arnold, S.R., Spracklen, D.V., 2019. Exploring the impacts of anthropogenic emission sectors on PM_2.5_ and human health in South and East Asia. Atmospheric Chem. Phys. 19, 11887–11910. https://doi.org/10.5194/acp-19-11887-2019](https://www.zotero.org/google-docs/?IgQrmR)

[Sadavarte, P., Venkataraman, C., 2014. Trends in multi-pollutant emissions from a technology-linked inventory for India: I. Industry and transport sectors. Atmos. Environ. 99, 353–364. https://doi.org/10.1016/j.atmosenv.2014.09.081](https://www.zotero.org/google-docs/?IgQrmR)

[Saikawa, E., Kim, H., Zhong, M., Avramov, A., Zhao, Y., Janssens-Maenhout, G., Kurokawa, J., Klimont, Z., Wagner, F., Naik, V., Horowitz, L.W., Zhang, Q., 2017. Comparison of emissions inventories of anthropogenic air pollutants and greenhouse gases in China. Atmospheric Chem. Phys. 17, 6393–6421. https://doi.org/10.5194/acp-17-6393-2017](https://www.zotero.org/google-docs/?IgQrmR)

[Saini, J., Dutta, M., Marques, G., 2020. A comprehensive review on indoor air quality monitoring systems for enhanced public health. Sustain. Environ. Res. 30, 6. https://doi.org/10.1186/s42834-020-0047-y](https://www.zotero.org/google-docs/?IgQrmR)

[Shaddick, G., Thomas, M.L., Green, A., Brauer, M., van Donkelaar, A., Burnett, R., Chang, H.H., Cohen, A., Dingenen, R.V., Dora, C., Gumy, S., Liu, Y., Martin, R., Waller, L.A., West, J., Zidek, J.V., Prüss-Ustün, A., 2018. Data integration model for air quality: a hierarchical approach to the global estimation of exposures to ambient air pollution. J. R. Stat. Soc. Ser. C Appl. Stat. 67, 231–253. https://doi.org/10.1111/rssc.12227](https://www.zotero.org/google-docs/?IgQrmR)

[Sharma, S., Bawase, M., Ghosh, P., Saraf, M., Goel, A., Suresh, R., dutta, A., Jhajra, ajeet, Kundu, S., sharma, ved, malik, jai, rehman, hafeez, Khandaskar, H., Mulla, S., Sharma, R., Bansal, A., Mane, S., Reve, S., Markad, A., Shaikh, A.R., 2018. Source Apportionment of PM2.5 & PM10 in Delhi NCR. https://doi.org/10.13140/RG.2.2.19621.55520](https://www.zotero.org/google-docs/?IgQrmR)

[Shen, G., Ru, M., Du, W., Zhu, X., Zhong, Q., Chen, Y., Shen, H., Yun, X., Meng, W., Liu, J., Cheng, H., Hu, J., Guan, D., Tao, S., 2019. Impacts of air pollutants from rural Chinese households under the rapid residential energy transition. Nat. Commun. 10, 3405. https://doi.org/10.1038/s41467-019-11453-w](https://www.zotero.org/google-docs/?IgQrmR)

[Shi, L., Wu, X., Yazdi, M.D., Braun, D., Awad, Y.A., Wei, Y., Liu, P., Di, Q., Wang, Y., Schwartz, J., Dominici, F., Kioumourtzoglou, M.-A., Zanobetti, A., 2020. Long-term effects of PM2$\cdot$5 on neurological disorders in the American Medicare population: a longitudinal cohort study. Lancet Planet. Health 4, e557–e565. https://doi.org/10.1016/S2542-5196(20)30227-8](https://www.zotero.org/google-docs/?IgQrmR)

[Shupler, M., Hystad, P., Birch, A., Miller-Lionberg, D., Jeronimo, M., Arku, R.E., Chu, Y.L., Mushtaha, M., Heenan, L., Rangarajan, S., Seron, P., Lanas, F., Cazor, F., Lopez-Jaramillo, P., Camacho, P.A., Perez, M., Yeates, K., West, N., Ncube, T., Ncube, B., Chifamba, J., Yusuf, R., Khan, A., Hu, B., Liu, X., Wei, L., Tse, L.A., Mohan, D., Kumar, P., Gupta, R., Mohan, I., Jayachitra, K.G., Mony, P.K., Rammohan, K., Nair, S., Lakshmi, P.V.M., Sagar, V., Khawaja, R., Iqbal, R., Kazmi, K., Yusuf, S., Brauer, M., 2020. Household and personal air pollution exposure measurements from 120 communities in eight countries: results from the PURE-AIR study. Lancet Planet. Health 4, e451–e462. https://doi.org/10.1016/S2542-5196(20)30197-2](https://www.zotero.org/google-docs/?IgQrmR)

[Siddiqui, A.R., Lee, K., Bennett, D., Yang, X., Brown, K.H., Bhutta, Z.A., Gold, E.B., 2009. Indoor carbon monoxide and PM2.5 concentrations by cooking  fuels in Pakistan. Indoor Air 19, 75–82. https://doi.org/10.1111/j.1600-0668.2008.00563.x](https://www.zotero.org/google-docs/?IgQrmR)

[Silva, R.A., Adelman, Z., Fry, M.M., West, J.J., 2016. The Impact of Individual Anthropogenic Emissions Sectors on the Global Burden of Human Mortality due to Ambient Air Pollution. Env. Health Perspect 124, 1776–1784. https://doi.org/10.1289/EHP177](https://www.zotero.org/google-docs/?IgQrmR)

[Smith, K.R., Bruce, N., Balakrishnan, K., Adair-Rohani, H., Balmes, J., Chafe, Z., Dherani, M., Hosgood, H.D., Mehta, S., Pope, D., Rehfuess, E., 2014. Millions Dead: How Do We Know and What Does It Mean? Methods Used in the Comparative Risk Assessment of Household Air Pollution. Annu Rev Public Health 35, 185–206. https://doi.org/10.1146/annurev-publhealth-032013-182356](https://www.zotero.org/google-docs/?IgQrmR)

[Smith, K.R., Pillarisetti, A., 2017. Household air pollution from solid cookfuels and Its Effects on Health 133–152.](https://www.zotero.org/google-docs/?IgQrmR)

[Snider, G., Weagle, C.L., Murdymootoo, K.K., Ring, A., Ritchie, Y., Stone, E., Walsh, A., Akoshile, C., Anh, N.X., Balasubramanian, R., Brook, J., Qonitan, F.D., Dong, J., Griffith, D., He, K., Holben, B.N., Kahn, R., Lagrosas, N., Lestari, P., Ma, Z., Misra, A., Norford, L.K., Quel, E.J., Salam, A., Schichtel, B., Segev, L., Tripathi, S., Wang, C., Yu, C., Zhang, Q., Zhang, Y., Brauer, M., Cohen, A., Gibson, M.D., Liu, Y., Martins, J.V., Rudich, Y., Martin, R.V., 2016. Variation in global chemical composition of PM_2.5_: emerging results from SPARTAN. Atmospheric Chem. Phys. 16, 9629–9653. https://doi.org/10.5194/acp-16-9629-2016](https://www.zotero.org/google-docs/?IgQrmR)

[Stanaway, J.D., Afshin, A., Gakidou, E., Lim, S.S., Abate, D., Abate, K.H., Abbafati, C., Abbasi, N., Abbastabar, H., Abd-Allah, F., Abdela, J., Abdelalim, A., Abdollahpour, I., Abdulkader, R.S., Abebe, M., Abebe, Z., Abera, S.F., Abil, O.Z., Abraha, H.N., Abrham, A.R., Abu-Raddad, L.J., Abu-Rmeileh, N.M.E., Accrombessi, M.M.K., Acharya, D., Acharya, P., Adamu, A.A., Adane, A.A., Adebayo, O.M., Adedoyin, R.A., Adekanmbi, V., Ademi, Z., Adetokunboh, O.O., Adib, M.G., Admasie, A., Adsuar, J.C., Afanvi, K.A., Afarideh, M., Agarwal, G., Aggarwal, A., Aghayan, S.A., Agrawal, A., Agrawal, S., Ahmadi, A., Ahmadi, M., Ahmadieh, H., Ahmed, M.B., Aichour, A.N., Aichour, I., Aichour, M.T.E., Akbari, M.E., Akinyemiju, T., Akseer, N., Al-Aly, Z., Al-Eyadhy, A., Al-Mekhlafi, H.M., Alahdab, F., Alam, K., Alam, S., Alam, T., Alashi, A., Alavian, S.M., Alene, K.A., Ali, K., Ali, S.M., Alijanzadeh, M., Alizadeh-Navaei, R., Aljunid, S.M., Alkerwi, N.T., Tsadik, A.G., Car, L.T., Tuzcu, E.M., Tymeson, H.D., Tyrovolas, S., Ukwaja, K.N., Ullah, I., Updike, R.L., Usman, M.S., Uthman, O.A., Vaduganathan, M., Vaezi, A., Valdez, P.R., Van Donkelaar, A., Varavikova, E., Varughese, S., Vasankari, T.J., Venkateswaran, V., Venketasubramanian, N., Villafaina, S., Violante, F.S., Vladimirov, S.K., Vlassov, V., Vollset, S.E., Vos, T., Vosoughi, K., Vu, G.T., Vujcic, I.S., Wagnew, F.S., Waheed, Y., Waller, S.G., Walson, J.L., Wang, Yafeng, Wang, Yanping, Wang, Y.-P., Weiderpass, E., Weintraub, R.G., Weldegebreal, F., Werdecker, A., Werkneh, A.A., West, J.J., Westerman, R., Whiteford, H.A., Widecka, J., Wijeratne, T., Winkler, A.S., Wiyeh, A.B., Wiysonge, C.S., Wolfe, C.D.A., Wong, T.Y., Wu, S., Xavier, D., Xu, G., Yadgir, S., Yadollahpour, A., Jabbari, S.H.Y., Yamada, T., Yan, L.L., Yano, Y., Yaseri, M., Yasin, Y.J., Yeshaneh, A., Yimer, E.M., Yip, P., Yisma, E., Yonemoto, N., Yoon, S.-J., Yotebieng, M., Younis, M.Z., Yousefifard, M., Yu, C., Zaidi, Z., Zaman, S.B., Zamani, M., Zavala-Arciniega, L., Zhang, A.L., Zhang, H., Zhang, K., Zhou, M., Zimsen, S.R.M., Zodpey, S., Murray, C.J.L., 2018. Global, regional, and national comparative risk assessment of 84 behavioural, environmental and occupational, and metabolic risks or clusters of risks for 195 countries and territories, 1990–2017: a systematic analysis for the Global Burden of Disease Study 2017. Lancet 392, 1923–1994. https://doi.org/10.1016/S0140-6736(18)32225-6](https://www.zotero.org/google-docs/?IgQrmR)

[Tagle, M., Pillarisetti, A., Hernandez, M.T., Troncoso, K., Soares, A., Torres, R., Galeano, A., Oyola, P., Balmes, J., Smith, K.R., 2019. Monitoring and modeling of household air quality related to use of different Cookfuels in Paraguay. Indoor Air 29, 252–262. https://doi.org/10.1111/ina.12513](https://www.zotero.org/google-docs/?IgQrmR)

[Tasmin, S., Ng, C.F.S., Stickley, A., Md, N., Saroar, G., Yasumoto, S., Watanabe, C., 2019. Effects of Short-term Exposure to Ambient Particulate Matter on the Lung Function of School Children in Dhaka, Bangladesh. Epidemiol. Camb. Mass 30 Suppl 1, S15–S23. https://doi.org/10.1097/EDE.0000000000001012](https://www.zotero.org/google-docs/?IgQrmR)

[Thunis, P., Degraeuwe, B., Pisoni, E., Trombetti, M., Peduzzi, E., Belis, C.A., Wilson, J., Clappier, A., Vignati, E., 2018. PM2.5 source allocation in European cities: A SHERPA modelling study. Atmos. Environ. 187, 93–106. https://doi.org/10.1016/j.atmosenv.2018.05.062](https://www.zotero.org/google-docs/?IgQrmR)

[Timmermans, R., Kranenburg, R., Manders, A., Hendriks, C., Segers, A., Dammers, E., Zhang, Q., Wang, L., Liu, Z., Zeng, L., Denier van der Gon, H., Schaap, M., 2017. Source apportionment of PM2.5 across China using LOTOS-EUROS. Atmos. Environ. 164, 370–386. https://doi.org/10.1016/j.atmosenv.2017.06.003](https://www.zotero.org/google-docs/?IgQrmR)

[Tong, D., Cheng, J., Liu, Y., Yu, S., Yan, L., Hong, C., Qin, Y., Zhao, H., Zheng, Y., Geng, G., Li, M., Liu, F., Zhang, Y., Zheng, B., Clarke, L., Zhang, Q., 2020. Dynamic projection of anthropogenic emissions in China: methodology and 2015–2050 emission pathways under a range of socio-economic, climate policy, and pollution control scenarios. Atmospheric Chem. Phys. 20, 5729–5757. https://doi.org/10.5194/acp-20-5729-2020](https://www.zotero.org/google-docs/?IgQrmR)

[Trombetti, M., Thunis, P., Bessagnet, B., Clappier, A., Couvidat, F., Guevara, M., Kuenen, J., López-Aparicio, S., 2018. Spatial inter-comparison of Top-down emission inventories in European urban areas. Atmos. Environ. 173, 142–156. https://doi.org/10.1016/j.atmosenv.2017.10.032](https://www.zotero.org/google-docs/?IgQrmR)

[Tsimpidi, A.P., Karydis, V.A., Pozzer, A., Pandis, S.N., Lelieveld, J., 2018. ORACLE 2-D (v2.0): an efficient module to compute the volatility and oxygen content of organic aerosol with a global chemistry–climate model. Geosci Model Dev 11, 3369–3389. https://doi.org/10.5194/gmd-11-3369-2018](https://www.zotero.org/google-docs/?IgQrmR)

[Turner, M.C., Jerrett, M., Pope, C.A., Krewski, D., Gapstur, S.M., Diver, W.R., Beckerman, B.S., Marshall, J.D., Su, J., Crouse, D.L., Burnett, R.T., 2015. Long-Term Ozone Exposure and Mortality in a Large Prospective Study. Am. J. Respir. Crit. Care Med. 193, 1134–1142. https://doi.org/10.1164/rccm.201508-1633OC](https://www.zotero.org/google-docs/?IgQrmR)

[Upadhyay, A., Dey, S., Chowdhury, S., Goyal, P., 2018. Expected health benefits from mitigation of emissions from major anthropogenic PM2.5 sources in India: Statistics at state level. Environ. Pollut. 242, 1817–1826. https://doi.org/10.1016/j.envpol.2018.07.085](https://www.zotero.org/google-docs/?IgQrmR)

[Van Donkelaar, A., Martin, R.V., Brauer, M., Hsu, N.C., Kahn, R.A., Levy, R.C., Lyapustin, A., Sayer, A.M., Winker, D.M., 2016. Global Estimates of Fine Particulate Matter using a Combined Geophysical-Statistical Method with Information from Satellites, Models, and Monitors. Environ. Sci. Technol. 50, 3762–3772. https://doi.org/10.1021/acs.est.5b05833](https://www.zotero.org/google-docs/?IgQrmR)

[Venkataraman, C., Bhushan, M., Dey, S., Ganguly, D., Gupta, T., Habib, G., Kesarkar, A., Phuleria, H., Raman, R.S., 2020. Indian Network Project on Carbonaceous Aerosol Emissions, Source Apportionment and Climate Impacts (COALESCE). Bull Am Meteorol Soc 101, E1052–E1068. https://doi.org/10.1175/BAMS-D-19-0030.1](https://www.zotero.org/google-docs/?IgQrmR)

[Wang, B., Eum, K.-D., Kazemiparkouhi, F., Li, C., Manjourides, J., Pavlu, V., Suh, H., 2020. The impact of long-term PM2.5 exposure on specific causes of death: exposure-response curves and effect modification among 53 million U.S. Medicare beneficiaries. Environ. Health 19, 20. https://doi.org/10.1186/s12940-020-00575-0](https://www.zotero.org/google-docs/?IgQrmR)

[WHO 2021, n.d. WHO global air quality guidelines: particulate matter (‎PM2.5 and PM10)‎, ozone, nitrogen dioxide, sulfur dioxide and carbon monoxide.](https://www.zotero.org/google-docs/?IgQrmR)

[Wyatt, L.H., Xi, Y., Kshirsagar, A., Di, Q., Ward-Caviness, C., Wade, T.J., Cascio, W.E., Rappold, A.G., 2020. Association of short-term exposure to ambient PM2.5 with hospital admissions and 30-day readmissions in end-stage renal disease patients: population-based retrospective cohort study. BMJ Open 10, e041177. https://doi.org/10.1136/bmjopen-2020-041177](https://www.zotero.org/google-docs/?IgQrmR)

[Xing, Y.-F., Xu, Y.-H., Shi, M.-H., Lian, Y.-X., 2016. The impact of PM2.5 on the human respiratory system. J. Thorac. Dis. 8, E69–E74. https://doi.org/10.3978/j.issn.2072-1439.2016.01.19](https://www.zotero.org/google-docs/?IgQrmR)

[Yu, W., Guo, Y., Shi, L., Li, S., 2020. The association between long-term exposure to low-level PM2.5 and mortality in the state of Queensland, Australia: A modelling study with the difference-in-differences approach. PLOS Med. 17, e1003141. https://doi.org/10.1371/journal.pmed.1003141](https://www.zotero.org/google-docs/?IgQrmR)

[Yun, X., Shen, G., Shen, H., Meng, W., Chen, Y., Xu, H., Ren, Y., Zhong, Q., Du, W., Ma, J., Cheng, H., Wang, Xilong, Liu, J., Wang, Xuejun, Li, B., Hu, J., Wan, Y., Tao, S., 2020. Residential solid fuel emissions contribute significantly to air pollution and associated health impacts in China. Sci. Adv. 6, eaba7621. https://doi.org/10.1126/sciadv.aba7621](https://www.zotero.org/google-docs/?IgQrmR)

[Yusuf, S., Joseph, P., Rangarajan, S., Islam, S., Mente, A., Hystad, P., Brauer, M., Kutty, V.R., Gupta, R., Wielgosz, A., AlHabib, K.F., Dans, A., Lopez-Jaramillo, P., Avezum, A., Lanas, F., Oguz, A., Kruger, I.M., Diaz, R., Yusoff, K., Mony, P., Chifamba, J., Yeates, K., Kelishadi, R., Yusufali, A., Khatib, R., Rahman, O., Zatonska, K., Iqbal, R., Wei, L., Bo, H., Rosengren, A., Kaur, M., Mohan, V., Lear, S.A., Teo, K.K., Leong, D., O’Donnell, M., McKee, M., Dagenais, G., 2020. Modifiable risk factors, cardiovascular disease, and mortality in 155\hphantom,722 individuals from 21 high-income, middle-income, and low-income countries (PURE): a prospective cohort study. Lancet 395, 795–808. https://doi.org/10.1016/S0140-6736(19)32008-2](https://www.zotero.org/google-docs/?IgQrmR)

[Zhao, B., Zheng, H., Wang, S., Smith, K.R., Lu, X., Aunan, K., Gu, Y., Wang, Y., Ding, D., Xing, J., Fu, X., Yang, X., Liou, K.-N., Hao, J., 2018. Change in household fuels dominates the decrease in PM2.5 exposure and premature mortality in China in 2005–2015. Proc. Natl. Acad. Sci. 115, 12401–12406. https://doi.org/10.1073/pnas.1812955115](https://www.zotero.org/google-docs/?IgQrmR)

[Zheng, B., Cheng, J., Geng, G., Wang, X., Li, M., Shi, Q., Qi, J., Lei, Y., Zhang, Q., He, K., 2021. Mapping anthropogenic emissions in China at 1 km spatial resolution and its application in air quality modeling. Sci. Bull. 66, 612–620. https://doi.org/10.1016/j.scib.2020.12.008](https://www.zotero.org/google-docs/?IgQrmR)

[Zheng, X., Orellano, P., Lin, H., Jiang, M., Guan, W., 2021. Short-term exposure to ozone, nitrogen dioxide, and sulphur dioxide and emergency department visits and hospital admissions due to asthma: A systematic review and meta-analysis. Environ. Int. 150, 106435. https://doi.org/10.1016/j.envint.2021.106435](https://www.zotero.org/google-docs/?IgQrmR)

1. The number in parenthesis indicates the contribution of HAP emissions to AAP considering the anthropogenic organic aerosols to be twice more toxic compared to the other aerosols [↑](#footnote-ref-1)
